# Natural-Cause Mortality and Long-Term Exposure to Particle Components: An Analysis of 19 European Cohorts within the Multi-Center ESCAPE Project

**DOI:** 10.1289/ehp.1408095

**Published:** 2015-02-24

**Authors:** Rob Beelen, Gerard Hoek, Ole Raaschou-Nielsen, Massimo Stafoggia, Zorana Jovanovic Andersen, Gudrun Weinmayr, Barbara Hoffmann, Kathrin Wolf, Evangelia Samoli, Paul H. Fischer, Mark J. Nieuwenhuijsen, Wei W. Xun, Klea Katsouyanni, Konstantina Dimakopoulou, Alessandro Marcon, Erkki Vartiainen, Timo Lanki, Tarja Yli-Tuomi, Bente Oftedal, Per E. Schwarze, Per Nafstad, Ulf De Faire, Nancy L. Pedersen, Claes-Göran Östenson, Laura Fratiglioni, Johanna Penell, Michal Korek, Göran Pershagen, Kirsten Thorup Eriksen, Kim Overvad, Mette Sørensen, Marloes Eeftens, Petra H. Peeters, Kees Meliefste, Meng Wang, H. Bas Bueno-de-Mesquita, Dorothea Sugiri, Ursula Krämer, Joachim Heinrich, Kees de Hoogh, Timothy Key, Annette Peters, Regina Hampel, Hans Concin, Gabriele Nagel, Andrea Jaensch, Alex Ineichen, Ming-Yi Tsai, Emmanuel Schaffner, Nicole M. Probst-Hensch, Christian Schindler, Martina S. Ragettli, Alice Vilier, Françoise Clavel-Chapelon, Christophe Declercq, Fulvio Ricceri, Carlotta Sacerdote, Claudia Galassi, Enrica Migliore, Andrea Ranzi, Giulia Cesaroni, Chiara Badaloni, Francesco Forastiere, Michail Katsoulis, Antonia Trichopoulou, Menno Keuken, Aleksandra Jedynska, Ingeborg M. Kooter, Jaakko Kukkonen, Ranjeet S. Sokhi, Paolo Vineis, Bert Brunekreef

**Affiliations:** 1 Institute for Risk Assessment Sciences, Utrecht University, Utrecht, the Netherlands; 2 Danish Cancer Society Research Center, Copenhagen, Denmark; 3 Department of Epidemiology, Lazio Regional Health Service, Rome, Italy; 4 Center for Epidemiology and Screening, Department of Public Health, University of Copenhagen, Copenhagen, Denmark; 5 Institute of Epidemiology and Medical Biometry, Ulm University, Ulm, Germany; 6 IUF–Leibniz Research Institute for Environmental Medicine, Düsseldorf, Germany, and; 7 Medical Faculty, University of Düsseldorf, Germany; 8 Institute of Epidemiology II, Helmholtz Zentrum München, German Research Center for Environmental Health, Neuherberg, Germany; 9 Department of Hygiene, Epidemiology and Medical Statistics, Medical School, University of Athens, Athens, Greece; 10 National Institute for Public Health and the Environment, Bilthoven, the Netherlands; 11 Centre for Research in Environmental Epidemiology (CREAL), Barcelona, Spain; 12 Consortium for Biomedical Research in Epidemiology and Public Health (CIBER en Epidemiología y Salud Pública-CIBERESP), Madrid, Spain; 13 MRC-HPA Centre for Environment and Health, Department of Epidemiology and Biostatistics, Imperial College London, London, United Kingdom; 14 University College London, CeLSIUS, London, United Kingdom; 15 Unit of Epidemiology and Medical Statistics, Department of Public Health and Community Medicine, University of Verona, Verona, Italy; 16 Department of Chronic Disease Prevention, National Institute for Health and Welfare, Helsinki, Finland; 17 Department of Environmental Health, National Institute for Health and Welfare, Kuopio, Finland; 18 Division of Environmental Medicine, Norwegian Institute of Public Health, Oslo, Norway; 19 Institute of Health and Society, University of Oslo, Oslo, Norway; 20 Institute of Environmental Medicine, Karolinska Institutet, Stockholm, Sweden; 21 Department of Medical Epidemiology and Biostatistics, Karolinska Institutet, Stockholm, Sweden; 22 Department of Molecular Medicine and Surgery, Karolinska Institutet, Stockholm, Sweden; 23 Aging Research Center, Department of Neurobiology, Care Sciences and Society, Karolinska Institutet, Stockholm, Sweden; 24 Section for Epidemiology, Department of Public Health, Aarhus University, Aarhus, Denmark; 25 Department of Cardiology, Aalborg University Hospital, Aalborg, Denmark; 26 Department of Epidemiology, Julius Center for Health Sciences and Primary Care, University Medical Center Utrecht, Utrecht, the Netherlands; 27 Department of Epidemiology and Biostatistics, School of Public Health, Faculty of Medicine, Imperial College, London, United Kingdom; 28 Department of Gastroenterology and Hepatology, University Medical Centre, Utrecht, the Netherlands; 29 Institute of Epidemiology I, Helmholtz Zentrum München, German Research Center of Environmental Health, Neuherberg, Germany; 30 Cancer Epidemiology Unit, Nuffield Department of Population Health, University of Oxford, Oxford, United Kingdom; 31 Agency for Preventive and Social Medicine, Bregenz, Austria; 32 Swiss Tropical and Public Health Institute, Basel, Switzerland; 33 University of Basel, Basel, Switzerland; 34 Department of Environmental and Occupational Health Sciences, University of Washington, Seattle, Washington, USA; 35 Inserm, Centre for research in Epidemiology and Population Health (CESP), Nutrition, Hormones and Women’s Health team, Villejuif, France; 36 University Paris Sud, Villejuif, France; 37 IGR, Villejuif, France; 38 French Institute for Public Health Surveillance (InVS), Saint-Maurice, France; 39 Human Genetics Foundation-HuGeF, Turin, Italy; 40 Unit of Cancer Epidemiology, AO Citta’ della Salute e della Scienza-University of Turin and Center for Cancer Prevention, Turin, Italy; 41 Environmental Health Reference Centre-Regional Agency for Environmental Prevention of Emilia-Romagna, Modena, Italy; 42 Hellenic Health Foundation, Athens, Greece; 43 TNO, Netherlands Organisation for Applied Scientific Research, Utrecht, the Netherlands; 44 Finnish Meteorological Institute, Helsinki, Finland; 45 Centre for Atmospheric and Instrumentation Research (CAIR), University of Hertfordshire, Hatfield, Hertfordshire, United Kingdom

## Abstract

**Background:**

Studies have shown associations between mortality and long-term exposure to particulate matter air pollution. Few cohort studies have estimated the effects of the elemental composition of particulate matter on mortality.

**Objectives:**

Our aim was to study the association between natural-cause mortality and long-term exposure to elemental components of particulate matter.

**Methods:**

Mortality and confounder data from 19 European cohort studies were used. Residential exposure to eight *a priori*–selected components of particulate matter (PM) was characterized following a strictly standardized protocol. Annual average concentrations of copper, iron, potassium, nickel, sulfur, silicon, vanadium, and zinc within PM size fractions ≤ 2.5 μm (PM_2.5_) and ≤ 10 μm (PM_10_) were estimated using land-use regression models. Cohort-specific statistical analyses of the associations between mortality and air pollution were conducted using Cox proportional hazards models using a common protocol followed by meta-analysis.

**Results:**

The total study population consisted of 291,816 participants, of whom 25,466 died from a natural cause during follow-up (average time of follow-up, 14.3 years). Hazard ratios were positive for almost all elements and statistically significant for PM_2.5_ sulfur (1.14; 95% CI: 1.06, 1.23 per 200 ng/m^3^). In a two-pollutant model, the association with PM_2.5_ sulfur was robust to adjustment for PM_2.5_ mass, whereas the association with PM_2.5_ mass was reduced.

**Conclusions:**

Long-term exposure to PM_2.5_ sulfur was associated with natural-cause mortality. This association was robust to adjustment for other pollutants and PM_2.5_.

**Citation:**

Beelen R, Hoek G, Raaschou-Nielsen O, Stafoggia M, Andersen ZJ, Weinmayr G, Hoffmann B, Wolf K, Samoli E, Fischer PH, Nieuwenhuijsen MJ, Xun WW, Katsouyanni K, Dimakopoulou K, Marcon A, Vartiainen E, Lanki T, Yli-Tuomi T, Oftedal B, Schwarze PE, Nafstad P, De Faire U, Pedersen NL, Östenson C-G, Fratiglioni L, Penell J, Korek M, Pershagen G, Eriksen KT, Overvad K, Sørensen M, Eeftens M, Peeters PH, Meliefste K, Wang M, Bueno-de-Mesquita HB, Sugiri D, Krämer U, Heinrich J, de Hoogh K, Key T, Peters A, Hampel R, Concin H, Nagel G, Jaensch A, Ineichen A, Tsai MY, Schaffner E, Probst-Hensch NM, Schindler C, Ragettli MS, Vilier A, Clavel-Chapelon F, Declercq C, Ricceri F, Sacerdote C, Galassi C, Migliore E, Ranzi A, Cesaroni G, Badaloni C, Forastiere F, Katsoulis M, Trichopoulou A, Keuken M, Jedynska A, Kooter IM, Kukkonen J, Sokhi RS, Vineis P, Brunekreef B. 2015. Natural-cause mortality and long-term exposure to particle components: an analysis of 19 European cohorts within the Multi-Center ESCAPE Project. Environ Health Perspect 123:525–533; http://dx.doi.org/10.1289/ehp.1408095

## Introduction

Studies have shown associations between long-term exposure to particulate matter air pollution and mortality, with exposure characterized as the mass concentration of particles ≤ 10 μm (PM_10_) or ≤ 2.5 μm (PM_2.5_) (Brook et al. 2010; Brunekreef and Holgate 2002). Although these studies have identified associations between exposure to particulate matter mass and mortality, there is still uncertainty as to which particle components are the most harmful. In addition, particulate matter effect estimates for long-term studies on mortality have differed among studies, and an explanation for this might be differences in the chemical composition of particulate matter (Hoek et al. 2013).

Particulate matter is a heterogeneous mixture varying spatially and temporally in chemical composition related to the sources from which it originates (Kelly and Fussell 2012; Stanek et al. 2011). Components for which associations with a range of health end points have been reported in epidemiological and/or toxicological studies include (transition) metals, elemental carbon, inorganic secondary aerosols (sulfate, nitrate), and organic components, but the evidence is not consistent (Kelly and Fussell 2012; Stanek et al. 2011).

Most studies that have assessed mortality in association with exposure to elemental components have been short-term exposure studies, and their results have varied considerably (Kelly and Fussell 2012; Stanek et al. 2011). Few studies have investigated mortality in relation to long-term exposure to particle components. A lack of spatially resolved elemental composition measurement data and exposure models for elemental composition partly explains this (De Hoogh et al. 2013). The U.S. Six Cities and American Cancer Society cohort studies have suggested an association between long-term exposure to sulfate and mortality (Dockery et al. 1993; Health Effects Institute 2000; Pope et al. 1995, 2002), but no other particle composition parameters have been evaluated in these studies. A cohort study, the California Teachers Study, found no statistically significant associations between all-cause mortality and long-term exposures to PM_2.5_ and several of its constituents, including elemental carbon, organic carbon (OC), sulfates, nitrates, iron, potassium, silicon, and zinc, although statistically significant associations were reported for more specific outcomes, especially ischemic heart disease mortality (Ostro et al. 2011).

In the framework of the multicenter ESCAPE (European Study of Cohorts for Air Pollution Effects) and TRANSPHORM (Transport related Air Pollution and Health impacts–Integrated Methodologies for Assessing Particulate Matter) projects, we added standardized exposure assessment for air pollution to mortality data from 19 ongoing cohort studies across Europe. Associations of particle mass (PM_2.5_, PM_10_, PM_coarse_, and PM_2.5_ absorbance) and nitrogen oxides (NO_2_ and NO_x_) with natural-cause mortality in the same cohorts have been reported previously (Beelen et al. 2014). We found a statistically significant elevated hazard ratio for PM_2.5_ of 1.07 [95% confidence interval (CI): 1.02, 1.13] per 5 μg/m^3^. In this paper we report associations with particle elemental composition in 19 European cohorts to assess whether specific components are associated with natural-cause mortality. A second aim was to assess whether the previously reported association with PM_2.5_ mass was explained by specific elements. Associations of particle composition and cardiovascular mortality have been published separately (Wang et al. 2014).

## Methods

As described earlier, the association between natural-cause mortality and particle components was analyzed in each cohort separately, following the analysis protocol of the ESCAPE study (Beelen et al. 2014). A common STATA script (StataCorp, College Station, TX, USA) was used which was explained in a training workshop for all local analysts. Cohort-specific results were sent to the coordinating institute [the Institute for Risk Assessment Sciences (IRAS), Utrecht University] for central evaluation. Cohort-specific effect estimates were combined by random-effects meta-analysis. Pooling of the cohort data was not possible due to data transfer and privacy issues.

**Figure 1:**
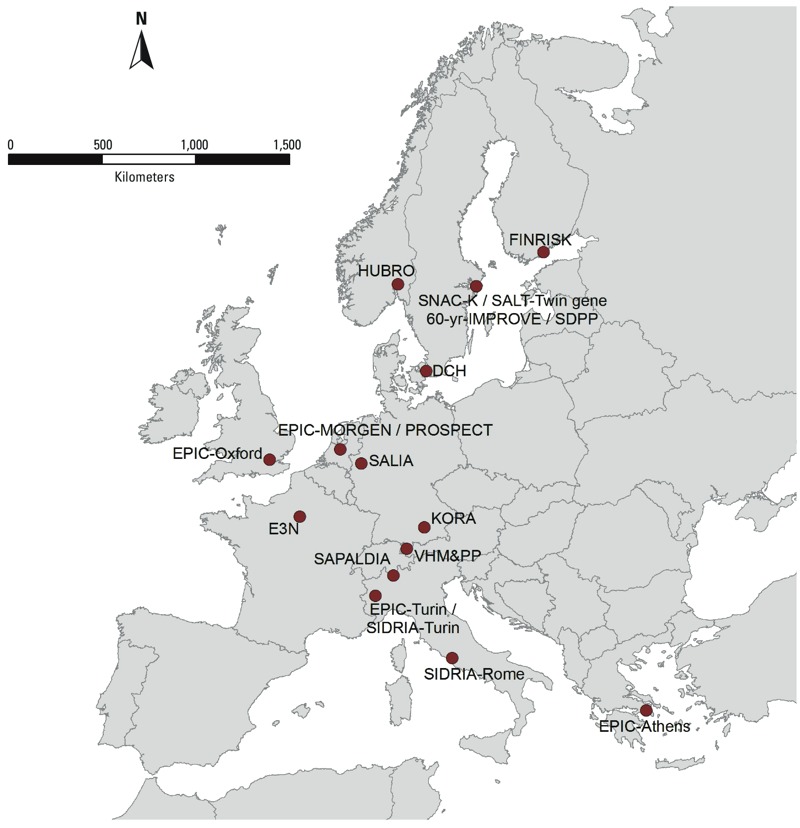
Cohort locations in which elements were measured.

*Study populations*. Nineteen cohorts from 12 countries across Europe were selected (Table 1 and Figure 1; see also Supplemental Material “Description of each cohort and study area”). The study areas of most cohorts consisted of a large city with surrounding smaller rural communities. Some cohorts included large regions of the country such as EPIC-MORGEN (European Prospective Investigation into Cancer and Nutrition– Monitoring Project on Risk Factors for Chronic Diseases) in the Netherlands, and the VHM&PP (Vorarlberg Health Monitoring & Promotion Programme) cohort in Austria. All included cohort studies were approved by the institutional medical ethics committees and undertaken in accordance with the Declaration of Helsinki. Each cohort study followed the rules for ethics and data protection set up in the country in which they were based. All participants gave consent according to national rules.

**Table 1:** Description of the included cohort studies.

Cohort^*a*^	*n* Total^*b*^	*n* NM^*c*^	Age (years) at baseline (mean ± SD)	Baseline period	Total follow-up time in person-years (mean follow-up)	Study area description
FINRISK, Finland	10,224	602	47.9 ± 13.2	1992; 1997; 2002; 2007	108,434 (10.6)	Greater Helsinki Area and Turku city and its rural surroundings
HUBRO, Norway	18,102	1,182	48.3 ± 15.2	2000–2001	173,798 (9.6)	City of Oslo
SNAC-K, Sweden	2,401	395	70.3 ± 8.1	2001–2004	15,568 (6.5)	City of Stockholm
SALT/Twin gene, Sweden	5,473	581	58.0 ± 9.9	1998–2002	47,767 (8.7)	Stockholm County
60-y/IMPROVE, Sweden	3,612	303	60.4 ± 0.1	1997–1999	40,612 (11.2)	Stockholm County
SDPP, Sweden	7,408	248	47.1 ± 5.0	1992–1998	102,831 (13.9)	Stockholm County
DCH, Denmark	35,458	3,770	56.7 ± 4.4	1993–1997	469,571 (13.2)	City of Copenhagen and surrounding areas
EPIC-MORGEN, Netherlands	16,446	795	43.9 ± 10.9	1993–1997	217,722 (13.2)	Cities of Amsterdam, Maastricht, and Doetinchem and surrounding rural areas
EPIC-PROSPECT, Netherlands	15,670	1,269	57.7 ± 6.0	1993–1997	202,809 (12.9)	City of Utrecht and surrounding rural areas
SALIA, Germany	4,352	618	54.5 ± 0.6	1985–1987; 1990–1994	81,093 (18.6)	Areas in the cities of Dortmund, Duisburg, Essen, Gelsenkirchen, and Herne situated in the Ruhr Area and the adjacent towns Borken and Dülmen
EPIC-Oxford, UK	8,598	443	45.0 ± 13.1	1993–2001	110,097 (12.6)	Urban and rural areas in a buffer of 10 km around London–Oxford area
KORA, Germany	8,399	673	49.5 ± 13.8	1994–1995; 1999–2001	88,592 (10.5)	City of Augsburg and two adjacent rural counties
VHM&PP, Austria	117,824	13,081	41.9 ± 14.9	1985–2005	2,039,328 (17.3)	State of Vorarlberg, excluding high mountain areas (> 600 m) and areas within 300 m of state border
SAPALDIA, Switzerland	1,250	65	42.0 ± 11.9	1991	20,294 (16.2)	City of Lugano
E3N, France	10,915	516	53.0 ± 6.8	1993–1996	147,021 (13.5)	City of Paris and surrounding rural areas
EPIC-Turin, Italy	7,261	302	50.4 ± 7.5	1993–1998	97,549 (13.4)	City of Turin
SIDRIA-Turin, Italy	5,054	129	44.2 ± 6.2	1999	55,667 (11.0)	City of Turin
SIDRIA-Rome, Italy	9,177	239	44.3 ± 6.0	1999	102,856 (11.2)	City of Rome
EPIC-Athens, Greece	4,192	255	49.4 ± 11.7	1994–1999	46,852 (11.2)	Greater Athens area
See Supplemental Material, “Description of each cohort and study area,” for full names of cohorts. ^***a***^Order of cohorts is north to south gradient. ^***b***^Total study population: number of observations with complete data for all model 3 (main model) covariates. ^***c***^Number of deaths from natural-cause mortality.						

*Mortality outcome definition*. In all cohorts, follow-up was based upon linkage to mortality registries. Natural-cause mortality was defined on the basis of the underlying cause of death recorded on death certificates as ICD-9 (*International Classification of Diseases, 9th Revision*) codes 001–779 and ICD-10 (*10th Revision*) codes A00–R99.

*Exposure assessment*. Particle composition concentrations at the baseline residential addresses of study participants were estimated by land use regression models following a standardized procedure described elsewhere (Beelen et al. 2013; De Hoogh et al. 2013; Eeftens et al. 2012a). Measurements of PM_2.5_ and PM_10_ were performed at 20 sites in each of the study areas. Within each study area, each of the 20 sites was measured during three 2-week periods (during summer, winter, and an intermediate season) within 1 year. The total measurement period over all study areas was between October 2008 and May 2011. PM filters were weighed before and after each measurement centrally at IRAS, Utrecht University, and were then sent to Cooper Environmental Services (Portland, OR, USA) to detect elements. All filters were analyzed for elemental composition using X-ray fluorescence (XRF) (De Hoogh et al. 2013). The three 2-week measurements were averaged, adjusting for temporal trends using data from a background monitoring site with continuous data (Cyrys et al. 2012; De Hoogh et al. 2013; Eeftens et al. 2012b).

In ESCAPE we *a priori* selected 8 of the 48 measured elements for further epidemiological evaluation based upon evidence of health effects (toxicity), representation of major anthropogenic sources, a high percentage of detected samples (> 75%), and good precision of measurements [coefficient of variation < 10% for all elements, except nickel (Ni) and vanadium (V) due to low concentration levels]. We selected copper (Cu), iron (Fe), and zinc (Zn) mainly for (non-tailpipe) traffic emissions; sulfur (S) for long-range transport; Ni and V for mixed oil burning/industry; silicon (Si) for crustal material; and potassium (K) for biomass burning (Viana et al. 2008). Elements may have multiple sources, so they do not necessarily represent single sources.

Predictor variables for nearby traffic intensity, population/household density, and land use were derived from geographic information systems (GIS), and were evaluated to explain spatial variation of annual average concentrations using land use regression modeling. If values of predictor variables for the cohort addresses were outside the range of values for the monitoring sites, values were truncated to the minimum and maximum values at the monitoring sites. Truncation was performed to prevent unrealistic predictions (e.g., related to too small distance to roads in GIS) and because we did not want to extrapolate the derived model beyond the range for which it was developed. Truncation has been shown to improve predictions at independent sites (Wang et al. 2012).

The results of the land use regression models were then used to estimate ambient particle composition concentration at the participants’ baseline addresses. A detailed description of the land use regression models for each of the eight elements is presented in Supplemental Material, Tables S1–S9.

*Statistical analyses*. Cohort-specific analyses. Cox proportional hazards models were used for the cohort specific analyses following the analysis protocol in the ESCAPE study (Beelen et al. 2014). Age was used as the time scale because of evidence of better adjustment for potential confounding by age (Thiébaut and Bénichou 2004). Censoring occurred at the time of death for non-natural causes, emigration, loss to follow-up for other reasons, or at end of follow-up, whichever came first. Air pollution exposure was analyzed as a linear time-invariant variable. Potential confounders were available from questionnaires at baseline. We specified three confounder models with increasing levels of adjustment *a priori*. Confounder models were selected based on previous cohort studies of air pollution and mortality and availability of data in a majority of the cohorts. The specific variables included as model covariates are listed for each cohort in Supplemental Material Tables S10–S28. Model 1 included only age (time axis), sex, and calendar time [year(s) of enrollment, continuous for baseline periods of ≤ 5 years]. Model 2 added the following individual-level variables (as available for the individual cohorts): smoking status (never/former/current), smoking intensity, smoking duration, environmental tobacco smoke, fruit intake, vegetables intake, alcohol consumption (linear and squared term), body mass index (BMI; linear and squared term), educational level (low, medium, high), occupational class (white/blue collar classification), employment status, and marital status. Model 3 added area-level socioeconomic status (SES) variables, including mean income, percentage of people with a low income, unemployment rate, and educational level or deprivation index, which were defined for most of the cohorts at the neighborhood or municipality level (see Supplemental Material, Tables S10–S28, for details).

Model 3 was selected as the main confounder model. Only subjects with complete information for model 3 variables were included in the analyses.

Two-pollutant models were conducted for each element by adjusting for particle mass (PM_2.5_, PM_10_, PM_coarse_), PM_2.5_ absorbance, NO_2_, NO_x_, and other elements in separate models. Because two pollutants may reflect the same source, two-pollutant models representing the independent effect of two pollutants may be difficult to interpret. Therefore, each two-pollutant model was restricted to data from studies for which the correlation between the two pollutants was ≤ 0.7.

In sensitivity analyses, we added prevalent hypertension and physical activity to model 3, and additionally adjusted for the classical cardiovascular risk factors prevalent diabetes and cholesterol level. Extended confounder models were used in sensitivity analyses because some potential effects of air pollution might be mediated (e.g., hypertension) or affected (e.g, physical activity) by these factors.

All cohort-specific analyses were done in STATA versions 10–12.

Meta-analysis. Meta-analyses of cohort-specific effect estimates were conducted using the DerSimonian–Laird method with random effects (DerSimonian and Laird 1986). To keep exposure contrasts broadly comparable among pollutants, we estimated hazard ratios (HRs) and 95% CIs for fixed increments corresponding to the mean difference between the 10th and 90th percentiles of measured pollutant concentrations across all study areas. Heterogeneity among cohorts was quantified by the *I*^2^ statistic and tested by the chi-square test from Cochran’s *Q*-statistic (Higgins and Thompson 2002).

We tested whether effect estimates differed for cohorts for which the land use regression model cross-validation explained variance was smaller or larger than 50% by computing the chi-square test of heterogeneity. In addition, we tested whether effect estimates differed by region of Europe (North: Sweden, Norway, Finland, Denmark; West and Middle: United Kingdom, the Netherlands, Germany, France, Austria, and Switzerland; South: Italy and Greece). We did not perform effect modification analyses for individual-level variables because this paper focuses on differences in effect estimates related to elemental composition. Only sex was an effect modifier for the association between PM_2.5_ and natural mortality in the same cohorts (Beelen et al. 2014).

All tests were two-sided and *p*-values of < 0.05 were deemed statistically significant.

All meta-analyses were conducted in STATA, version 12.1.

## Results

*Characteristics of the study population*. The total study population consisted of 291,816 participants contributing 4,168,461 person-years at risk (average time of follow-up, 14.3 years), of whom 25,466 died from a natural cause during follow-up (Table 1). Cohorts were recruited mostly in the 1990s. Cohorts differed in the number of participants, the mean baseline age, and the availability of specific covariate data (Table 2; see also Supplemental Material, Tables S10–S28). Age, sex, smoking status (current, former, or never smoker), and an area-level SES variable were available for all cohorts. Smoking intensity (average cigarettes/day) and duration (years of smoking) were available as continuous variables for all cohorts except the VHM&PP (Vorarlberg state) and E3N (Etude Epidémiologique auprès de femmes de la Mutuelle Générale de l’Education Nationale; Paris and surrounding rural areas) cohorts, for which only smoking status was available. VHM&PP had data on occupation and employment status, but not on education. On average, we had complete model 3 covariate information for > 90% of cohort participants.

**Table 2:** Population characteristics of the included cohort studies at baseline.

Cohort^*a*^	Percent women	Percent never smokers	Cigarettes/day^*b*^	Years of smoking^*b*^	BMI (kg/m^2^)^*b*^	Fruit intake^*c*^	Alcohol intake^*d*^	Percent married/living with partner	Percent low educational level	Percent employed/self-employed
FINRISK, Finland	54	45	3.8 ± 7.8	8.6 ± 12.2	26.4 ± 4.6	66	0.9 ± 1.3	70	31	69
HUBRO, Norway	56	46	6.8 ± 8.4	11.6 ± 14.4	25.7 ± 4.1	40	51	50	18	73
SNAC-K, Sweden	60	44	7.1 ± 9.5	9.8 ± 15.2	26.0 ± 4.1	NA	22	54	21	29
SALT/Twin gene, Sweden	56	39	8.5 ± 9.7	16.7 ± 17.3	28.6 ± 4.1	NA	NA	68	22	NA
60-y/IMPROVE, Sweden	53	41	8.0 ± 9.1	15.2 ± 16.4	26.8 ± 4.2	64	8.9 ± 9.7	72	28	51
SDPP, Sweden	62	37	8.5 ± 8.8	12.3 ± 12.4	25.6 ± 4.0	92	1.3 ± 1.9	84	26	92
DCH, Denmark	54	36	6.3 ± 10.4	18.7 ± 17.1	26.0 ± 4.1	183.2 ± 151.2	21.7 ± 22.8	69	30	80
EPIC-MORGEN, Netherlands	54	35	10.4 ± 11.1	14.3 ± 13.7	25.2 ± 4.0	171.9 ± 129.2	12.7 ± 18.0	68	12	NA
EPIC-PROSPECT, Netherlands	100	45	5.7 ± 7.4	15.2 ± 16.5	25.5 ± 4.1	231.6 ± 139.2	9.0 ± 12.4	77	22	NA
SALIA, Germany	100	75	2.6 ± 6.6	4.4 ± 10.5	NA	NA	NA	NA	29	NA
EPIC-Oxford, UK	75	60	5.5 ± 8.8	7.3 ± 11.5	24.3 ± 4.3	253.6 ± 216.5	10.0 ± 12.3	67	34	77
KORA, Germany	51	44	9.2 ± 13.3	12.0 ± 14.2	27.2 ± 4.6	60	16.3 ± 22.3	76	13	58
VHM&PP, Austria	56	70	NA	NA	24.8 ± 4.3	NA	NA	68	NA	69
SAPALDIA, Switzerland	56	45	11.1 ± 14.4	11.1 ± 13.0	23.8 ± 3.9	NA	NA	58	11	81
E3N, France	100	49	NA	NA	22.8 ± 3.3	236.2 ± 162.5	12.4 ± 15.4	NA	5	NA
EPIC-Turin, Italy	48	43	7.2 ± 8.2	17.6 ± 16.3	25.3 ± 3.8	318.2 ± 182.2	18.1 ± 20.3	86	44	NA
SIDRIA-Turin, Italy	52	38	9.3 ± 10.2	11.3 ± 10.6	NA	NA	NA	95	18	72
SIDRIA-Rome, Italy	53	35	10.1 ± 10.5	11.7 ± 10.4	NA	NA	NA	100	45	NA
EPIC-Athens, Greece	55	40	1.7 ± 15.0	10.8 ± 13.1	27.5 ± 4.5	402.6 ± 258.2	9.2 ± 14.5	78	24	67
NA, not available or available with large number of missings (e.g., BMI in SALIA and smoking variables in E3N). See Supplemental Material, “Description of each cohort and study area,” for full names of cohorts. A detailed description of each cohort can be found in Supplemental Material, Tables S10–S28. ^***a***^Order of cohorts is north to south gradient. ^***b***^Mean ± SD. ^***c***^Mean ± SD (g/day) or percentage reporting daily fruit consumption. For SDPP it is percentage daily/weekly fruit consumption. ^***d***^Mean ± SD (g/day) or percentage reporting daily alcohol consumption. For FINRISK it is number of glasses of alcoholic drink during last week. For SDPP it number of glasses of alcoholic drinks per day. For HUBRO it is the percentage reporting weekly alcohol consumption.										

**Figure 2:**
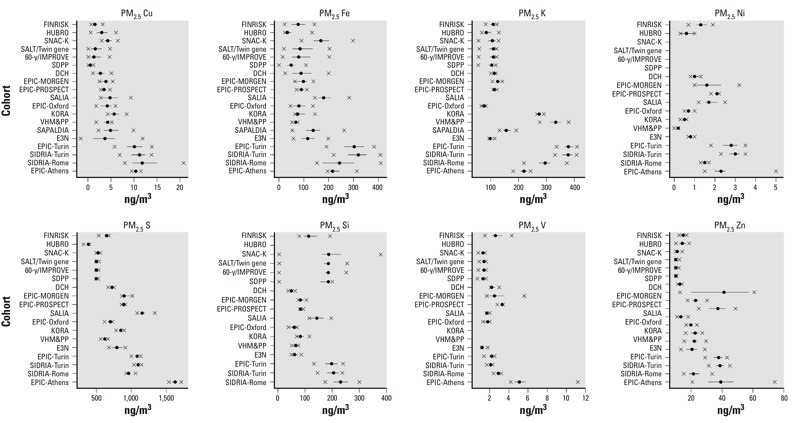
Estimated annual mean PM_2.5_ elemental composition concentrations (ng/μg^3^) at participant addresses in each cohort. The solid circle and bars shows the median and 25th and 75th percentiles of elemental composition concentrations; the x shows the 5th and 95th percentile values.

*Air pollution exposure*. Substantial variations of estimated annual mean concentrations at participant addresses were observed within and between the majority of cohorts and elements [Figure 2 (for PM_2.5_ elemental composition concentrations); see also Supplemental Material, Figure S1 (for PM_10_ elemental composition concentrations)]. The largest within-cohort contrasts were found for Cu, Fe, Si, and Zn, with the largest contrasts generally found in South European study areas. The main exception was Si, for which the largest within-area contrast was found in the North European study areas (see Supplemental Material, Figure S1). The smallest within-cohort contrasts were found for S. Higher concentrations of most elements were observed in southern study areas. Estimated annual mean S in PM_2.5_ concentrations, for example, show a steady increasing north–south gradient with averages from 635 ng/m^3^ for FINRISK, Finland, to 1,626 ng/m^3^ for EPIC-Athens, Greece. Correlations between elements and particle mass varied considerably among elements and cohorts; average correlations between elements and mass (in the same PM size fraction) were approximately 0.5, with a range from about 0.3 to about 0.7 (see Supplemental Material, Table S29), indicating that associations with individual elements could be estimated after adjusting for PM mass in most cohorts.

Good land use regression exposure models were developed for Cu, Fe, and Zn in both fractions (PM_10_ and PM_2.5_), as indicated by average cross-validation explained variances (*R*^2^) between 55% and 81%, although *R*^2^ values varied between areas (see Supplemental Material, Tables S1–S9). Traffic variables were the dominant predictors, reflecting non-tailpipe emissions (De Hoogh et al. 2013). In general, models for the other elements performed moderately well, with average cross-validation *R*^2^ values between about 50% and about 60%. However, for PM_2.5_ S the average cross-validation *R*^2^ was 30% (range, 2–67%; see Supplemental Material, Table S6), consistent with the relatively low spatial variation of S concentrations.

**Figure 3:**
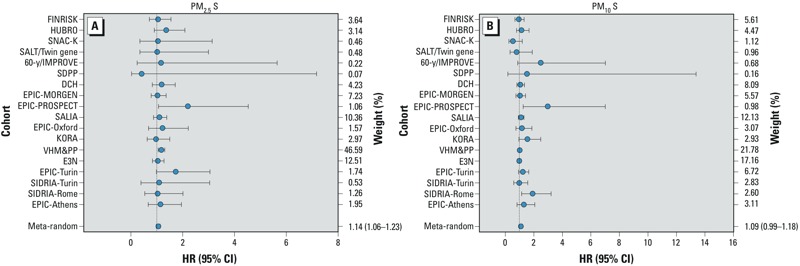
Adjusted hazard ratio (HR) between natural-cause mortality and (*A*) a 200-ng/m^3^ increment in PM_2.5_ S and (*B*) a 200-ng/m^3^ increment in PM_10_ S (using main model 3): results from cohort-specific analyses and from random-effects meta-analyses.

*Single-pollutant results*. Positive HRs were estimated for almost all exposures, with a statistically significant association for PM_2.5_ S (HR = 1.14; 95% CI: 1.06, 1.23 per 200 ng/m^3^) (Table 3, Figure 3; see also Supplemental Material, Figures S2–S15). Borderline statistically significant associations (*p* > 0.05 and ≤ 0.10) were found for PM_2.5_ Si (HR = 1.09; 95% CI: 0.99, 1.09 per 100 ng/m^3^), PM_10_ Ni (HR = 1.09; 95% CI: 1.00, 1.19 per 2 ng/m^3^), and PM_10_ K (HR = 1.03; 95% CI: 1.00, 1.06 per 100 ng/m^3^). The evidence for an association was smaller for Zn and V. Estimates did not support associations of mortality with the non-tailpipe traffic pollutants Cu and Fe. In general, HRs based on confounder model 1 (adjusted for calendar year and sex only) were the highest, whereas HRs moved closer to the null after adjustment for individual-level confounders (model 2). Sensitivity analyses showed that smoking variables especially were responsible for this decrease (Beelen et al. 2014). In contrast, additional adjustment for area-level SES variables (model 3) had relatively little influence on HRs (Table 3). Cohort-specific HRs for PM_2.5_ S were > 1 for all cohorts, except for SDPP (Stockholm Diabetes Prevention Program) and KORA (Cooperative Health Research in the Augsburg Region) (Figure 3). There was no statistical evidence of heterogeneity among the individual cohort effect estimates for PM_2.5_ S (*I*^2^ = 0, *p* = 0.94). Average correlation between PM_2.5_ S and PM_10_ S over the different cohorts was 0.56 with a range of 0.18–1.00 (data not shown). The HR for PM_10_ S was also positive (HR = 1.09; 95% CI: 0.99, 1.19 per 200 ng/m^3^), although not statistically significant (Figure 3).

**Table 3:** Association between natural-cause mortality and exposure to elemental composition of PM: results from random-effects meta-analyses [HR (95% CI)] using main confounder models 1, 2, and 3.^*a*^

Exposure	No. of cohorts	Model 1^*b*^	Model 2^*b*^	Model 3^*b*^	*p*-Value model 3	*I*^2^ (*p*-value)^*c*^
PM_2.5_ Cu	19	1.08 (1.00, 1.17)	1.00 (0.94, 1.06)	0.98 (0.92, 1.04)	0.54	16.4 (0.25)
PM_10_ Cu	19	1.07 (1.00, 1.15)	1.02 (0.95, 1.08)	1.01 (0.95, 1.07)	0.83	43.5 (0.02)
PM_2.5_ Fe	19	1.12 (1.05, 1.18)	1.04 (0.99, 1.10)	1.03 (0.98, 1.09)	0.20	10.1 (0.33)
PM_10_ Fe	19	1.08 (1.02, 1.15)	1.03 (0.97, 1.09)	1.02 (0.97, 1.08)	0.44	43.9 (0.02)
PM_2.5_ Zn	19	1.07 (1.00, 1.15)	1.04 (1.00, 1.08)	1.03 (0.99, 1.08)	0.17	21.4 (0.19)
PM_10_ Zn	19	1.09 (1.01, 1.17)	1.04 (1.00, 1.09)	1.04 (0.99, 1.09)	0.18	31.5 (0.09)
PM_2.5_ S	18^*d*^	1.29 (1.11, 1.50)	1.16 (1.08, 1.25)	1.14 (1.06, 1.23)	0.003	0.0 (0.94)
PM_10_ S	18^*d*^	1.23 (1.07, 1.42)	1.09 (1.00, 1.19)	1.09 (0.99, 1.19)	0.11	29.8 (0.11)
PM_2.5_ Ni	14^*e*^	1.12 (1.02, 1.22)	1.05 (0.97, 1.15)	1.05 (0.97, 1.13)	0.27	20.3 (0.23)
PM_10_ Ni	17^*f*^	1.22 (1.05, 1.41)	1.09 (1.00, 1.19)	1.09 (1.00, 1.19)	0.08	30.3 (0.12)
PM_2.5_ V	15^*g*^	1.22 (1.03, 1.44)	1.07 (0.95, 1.20)	1.07 (0.93, 1.23)	0.35	32.5 (0.11)
PM_10_ V	18^*d*^	1.07 (0.93, 1.24)	1.04 (0.96, 1.12)	1.03 (0.95, 1.12)	0.46	5.7 (0.39)
PM_2.5_ Si	16^*h*^	1.18 (1.03, 1.34)	1.10 (0.99, 1.21)	1.09 (0.99, 1.09)	0.10	31.6 (0.11)
PM_10_ Si	18^*d*^	1.13 (1.00, 1.28)	1.04 (0.97, 1.11)	1.03 (0.97, 1.11)	0.37	47.6 (0.01)
PM_2.5_ K	18^*i*^	1.06 (0.98, 1.14)	1.05 (0.99, 1.11)	1.07 (0.99, 1.15)	0.12	28.6 (0.13)
PM_10_ K	18^*j*^	1.05 (0.99, 1.12)	1.03 (1.00, 1.06)	1.03 (1.00, 1.06)	0.08	0.0 (0.74)
^***a***^HRs are presented for the following increments: 5 ng/m^3^ PM_2.5_ Cu, 20 ng/m^3^ PM_10_ Cu, 100 ng/m^3^ PM_2.5_ Fe, 500 ng/m^3^ PM_10_ Fe, 10 ng/m^3^ PM_2.5_ Zn, 20 ng/m^3^ PM_10_ Zn, 200 ng/m^3^ PM_2.5_ S, 200 ng/m^3^ PM_10_ S, 1 ng/m^3^ PM_2.5_ Ni, 2 ng/m^3^ PM_10_ Ni, 2 ng/m^3^ PM_2.5_ V, 3 ng/m^3^ PM_10_ V, 100 ng/m^3^ PM_2.5_ Si, 500 ng/m^3^ PM_10_ Si, 50 ng/m^3^ PM_2.5_ K, and 100 ng/m^3^ PM_10_ K. ^***b***^Model 1 was adjusted for sex and calendar time; model 2 was also adjusted for smoking status, smoking intensity, smoking duration, environmental tobacco smoke, fruit intake, vegetables intake, alcohol consumption, BMI, educational level, occupational class, employment status, marital status; and model 3 was further adjusted for area-level SES. ^***c***^*I*^2^ and Cochran’s *Q*-test for heterogeneity for model 3. ^***d***^No modeled air pollution estimates were available for SAPALDIA. ^***e***^No modeled air pollution estimates were available for SNAC-K, SALT/Twin gene, 60-y/IMPROVE, SDPP. ^***f***^No modeled air pollution estimates were available for HUBRO, SAPALDIA. ^***g***^No modeled air pollution estimates were available for HUBRO, KORA, VHM&PP, SAPALDIA. ^***h***^No modeled air pollution estimates were available for HUBRO, SAPALDIA, EPIC-Athens. ^***i***^No modeled air pollution estimates were available for SALIA. ^***j***^No modeled air pollution estimates were available for HUBRO.						

For the other elements there was more heterogeneity among individual cohort effect estimates, although for most elements heterogeneity was low (*I*^2^ < 25%) to moderate (*I*^2^ = 25–50%) and not statistically significant (Table 3; see also Supplemental Material, Figures S2–S15).

*Two-pollutant results*. Results from the two-pollutant models suggested that the associations of elements were generally robust to adjustment for other elements and pollutants (see Supplemental Material, Figures S16 and S17). We also investigated whether the previously reported association between natural-cause mortality and PM_2.5_ mass (Beelen et al. 2014) was robust to adjustment for PM_2.5_ S. The median correlation between PM_2.5_ and PM_2.5_ S over the cohorts was 0.53 (range, 0.26–0.86) (see Supplemental Material, Table S29). The combined effect estimate for PM_2.5_ S from the two-pollutant model adjusted for PM_2.5_ did not differ from the single-pollutant model estimate (Table 4). However, the HR for PM_2.5_ was closer to the null and statistically nonsignificant when adjusted for PM_2.5_ S (HR = 1.07; 95% CI: 1.02, 1.13 vs. HR = 1.02; 95% CI: 0.96, 1.09 per 5 μg/m^3^). In addition, Table 4 shows the two-pollutant model results for PM_2.5_ Si, PM_10_ K, and PM_10_ Ni because the single-pollutant associations for these elements were borderline statistically significant. After adjustment for PM_2.5_ S, associations with PM_10_ Ni (HR = 1.09; 95% CI: 0.98, 1.22 vs. HR = 1.06; 95% CI: 0.95, 1.18 per 2 ng/m^3^) were slightly reduced (Table 4).

**Table 4:** Results from random-effects meta-analyses from single-pollutant and two-pollutant models for association with natural-cause mortality (using main model 3) [HR (95% CI)].*^a^*

Exposure	Adjusted for	Single-pollutant	Two-pollutant
PM_2.5_ S^*b*^	PM_2.5_	1.15 (1.06, 1.24)	1.13 (1.03, 1.24)
PM_2.5_ S^*c*^	PM_10_ Ni	1.14 (1.04, 1.25)	1.14 (1.04, 1.25)
PM_2.5_ S^*d*^	PM_2.5_ Si	1.14 (1.05, 1.23)	1.13 (1.04, 1.22)
PM_2.5_ S^*e*^	PM_10_ K	1.16 (1.06, 1.27)	1.15 (1.05, 1.26)
PM_2.5_^*b*^	PM_2.5_ S	1.07 (1.02, 1.13)	1.02 (0.96, 1.09)
PM_10_ Ni^*c*^	PM_2.5_ S	1.09 (0.98, 1.22)	1.06 (0.95, 1.18)
PM_2.5_ Si^*d*^	PM_2.5_ S	1.09 (0.98, 1.21)	1.08 (0.97, 1.20)
PM_10_ K^*e*^	PM_2.5_ S	1.03 (0.99, 1.08)	1.02 (0.98, 1.06)
^***a***^Limited to studies for which correlation between two pollutants was < 0.7. HRs are presented for the following increments: 200 ng/m^3^ PM_2.5_ S, 5 μg/m^3^ PM_2.5_, 2 ng/m^3^ PM_10_ Ni, 100 ng/m^3^ PM_2.5_ Si, 100 ng/m^3^ PM_10_ K. ^***b***^FINRISK and SAPALDIA not included. ^***c***^HUBRO, SALIA, and SAPALDIA not included. ^***d***^HUBRO, SAPALDIA, and EPIC-Athens not included. ^***e***^FINRISK, HURBO, and SIDRIA-Rome not included.			

*Sensitivity analyses*. Additional adjustment for hypertension and physical activity, and for diabetes and cholesterol, had little effect on combined HRs compared with model 3 HRs (see Supplemental Material, Table S30).

Because the VHM&PP cohort had a weight of approximately 47% in the pooled PM_2.5_ S analyses (Figure 3), we conducted a sensitivity analyses without this cohort. Confidence intervals became slightly wider, but PM_2.5_ S HR remained similar after exclusion of the VHM&PP cohort (HR = 1.12; 95% CI: 1.01, 1.24 compared with HR = 1.14; 95% CI: 1.06, 1.23 before exclusion). Effect estimates for all elements were similar for the cohorts for which the land use regression model cross-validation explained variance was < 50% or > 50% (e.g., for PM_2.5_ S, HR = 1.12; 95% CI: 1.01, 1.25; *n* = 14 and HR = 1.16; 95% CI: 1.05, 1.28; *n* = 4, respectively) (*p* = 0.65). PM_2.5_ S effect estimates were also not statistically different between the cohorts in different regions: 1.17 (95% CI: 0.94, 1.45) for North (*n* = 7), 1.13 (95% CI: 1.04, 1.23) for West and Middle (*n* = 7), and 1.27 (95% CI: 0.92, 1.75) for South (*n* = 4) (*p* = 0.78). For the other elements also no significant differences were found between effect estimates based on validation *R*^2^ or region (data not shown).

## Discussion

Long-term exposure to PM_2.5_ S was positively associated with natural-cause mortality, with no indication of heterogeneity among individual cohort effect estimates.

The association between PM_2.5_ S and mortality was robust to adjustment for co-pollutants including PM_2.5_ mass. The PM_2.5_ mass effect estimate was reduced and became statistically nonsignificant when adjusted for PM_2.5_ S.

*Comparison of S mortality associations with previous studies*. Only a few studies have estimated associations of mortality with long-term exposures to particle components. Sulfate has received the most attention in epidemiological studies. Elemental sulfur is assumed to be present as a marker for sulfate. Several cohort studies suggested an association between long-term exposure to sulfate and mortality. An association between sulfate and mortality was reported in the Harvard Six Cities study (Dockery et al. 1993). The adjusted HR comparing the cities with the highest and lowest sulfate concentrations (a contrast of 8 μg/m^3^) was 1.26 (95% CI: 1.08, 1.47), corresponding to an HR of 1.03 (95% CI: 1.01, 1.05) per 1 μg/m^3^. Within the initial American Cancer Society (ACS) study, the adjusted HR of all-cause mortality for areas with the highest and lowest concentrations of sulfate (19.9 μg/m^3^ contrast) was 1.15 (95% CI: 1.09, 1.22) (Pope et al. 1995), resulting in an HR of 1.01 (95% CI: 1.00, 1.01) per 1 μg/m^3^. Pope et al. (2002) investigated additional years of follow-up in the ACS study and estimated an HR for sulfate and natural mortality of about 1.01 (95% CI: 1.00, 1.01) per 1 μg/m^3^ (Pope et al. 2002). A recent analysis of the ACS cohort reported that sulfate, elemental carbon, and ozone all had positive and statistically significant associations with all-cause mortality, but sulfate had the most robust association (HR = 1.01; 95% CI: 1.00, 1.01 per 1 μg/m^3^) (Smith et al. 2009). In the recent National Particle Component Toxicity (NPACT) initiative, a similar risk for the association between sulfur exposure and all-cause mortality (HR = 1.09 per 200 ng/m^3^) was estimated using ACS cohort data (Lippmann et al. 2013). Within the NPACT initiative also data from the Women’s Health Initiative–Observational Study (WHI-OS) cohort were used to study the association with cardiovascular mortality and (fatal and nonfatal) cardiovascular events (Vedal et al. 2013). Long-term exposure to air pollutant concentrations was estimated with a national exposure spatial model. No association was found with all cardiovascular deaths and sulfur (HR = 1.01, 95% CI: 0.92, 1.12 per 0.25 μg/m^3^), but the association with cardiovascular events was statistically significant (HR 1.09; 95% CI: 1.05, 1.14 per 0.25 μg/m^3^). A cohort study of approximately 45,000 active and former female public school professionals in the California Teachers Study investigated the association between mortality and long-term exposures to PM_2.5_ and several of its constituents, including elemental carbon, organic carbon, sulfates, nitrates, Fe, K, Si, and Zn (Ostro et al. 2011). Participants whose residential addresses were within 8 or 30 km of a monitor collecting PM_2.5_ constituent data were included in the analyses. No statistically significant associations between all-cause mortality and PM_2.5_ mass or any of its measured constituents were reported. The HR for sulfate was 1.06 (95% CI: 0.97, 1.16) for an interquartile range contrast of 2.2 μg/m^3^, corresponding to an HR of 1.03 per 1 μg/m^3^. However, the HR for sulfate and ischemic heart disease mortality was 1.48 (95% CI: 1.20, 1.82) for an interquartile range contrast of 2.2 μg/m^3^.

The estimated effect of PM_2.5_ S on natural-cause mortality in our study population (HR = 1.14 per 0.2 μg/m^3^ S) corresponds to an HR of 1.24 (95% CI: 1.10, 1.41) per 1 μg/m^3^ sulfate, assuming all S is present as sulfate (sulfate to S ratio of 3). Our effect estimate is thus much larger than the estimate from the U.S. cohort studies that investigated total mortality. A major difference between our study and these U.S. studies is that our study was based upon contrasts within study areas, whereas the U.S. studies focused on between-area contrasts. Sulfate is mostly formed in the atmosphere by oxidation of gaseous sulfur dioxide (SO_2_) emissions [U.S. Environmental Protection Agency (EPA) 2004]. Sulfate is concentrated in fine particles that can be transported over long distances, resulting in a high regional background with typically small spatial variation within metropolitan areas (U.S. EPA 2004). Most of our study areas comprised a major city and smaller surrounding communities, with some cohorts covering a larger area (e.g., the Vorarlberg region). Consistently, the exposure contrast in our study was much smaller than in the U.S. studies, both for the S measurements (De Hoogh et al. 2013) and cohort exposures. Measured urban background PM_2.5_ S concentrations were on average 9% higher than regional background concentrations. Concentrations at traffic sites were only 2% higher than at urban background sites. Predictor variables in the land use regression models for PM_2.5_ S included especially traffic at various scales, population or address density, and urban green space (see Supplemental Material, Tables S1–S9). Presumably because of the small measured within-study area contrasts, the average cross-validation *R*^2^ was 30% for PM_2.5_ S, with a range of 7–70%. Because land use regression models were developed for each study area separately, we could not exploit between-study area variations in PM_2.5_ S that would have improved the model performance. In the ESCAPE study, which focuses on within-area contrasts in pollution, these models reflect a combination of variation in primary sulfate emissions and secondary sulfate formation (De Hoogh et al. 2013). Depending on meteorological conditions, SO_2_ to sulfate conversion rates of 1–5% per hour have been estimated (U.S. EPA 2004), implying that some conversion already occurs at scales of 10–50 km (a typical wind speed is 10 km/hr). A study in Berlin, Germany, documented measurable sulfate formation within 50 km of the source (Lammel et al. 2005).

PM_2.5_ mass also was associated with mortality in the three U.S. studies (Dockery et al. 1993; Pope et al. 1995, 2002). However, sulfate concentrations were highly correlated with PM_2_*_._*_5_ mass concentrations in the U.S studies, and thus associations between mortality and sulfate may be difficult to distinguish from associations between mortality and PM_2.5_ mass. The median correlation between estimated PM_2.5_ and PM_2.5_ S over the 19 cohorts in our study was 0.53 (range, 0.26–0.86), which made it possible to estimate mutually adjusted associations with PM_2.5_ S and PM_2.5_ mass. The lower correlation in our study probably reflects the finer spatial resolution at which concentrations were estimated. The median correlation of measured within-area contrast in PM_2.5_ and S was very similar (0.6) to the median correlation within cohorts, suggesting that the moderate model *R*^2^ values for S did not artificially induce the low correlation.

Another study that reported evidence of effects of sulfur on mortality was an intervention study in Hong Kong that studied the effects of limiting the sulfur content of fuel oils used in both power plants and vehicles (Hedley et al. 2002). Initial findings indicated a decrease in sulfur dioxide that was associated with prompt and persistent reductions in mortality, suggesting that higher mortality before the limitation may have been related to sulfate and/or SO_2_. Subsequent analysis, however, revealed that the reduction in SO_2_ was highly correlated with reductions in both V and Ni derived from residual oil emissions (Hedley et al. 2006). In our study correlations between elements were smaller, suggesting that the association between PM_2.5_ S and mortality is not explained by exposure to other elements such as V and Ni. This is also supported by the robust HRs for PM_2.5_ S after adjustment for co-pollutants. However, we cannot rule out the possibility that the association with PM_2.5_ S may be attributable to other correlated PM components.

*Interpretation of S associations*. Toxicological studies have provided little support for a causal effect of sulfate, despite fairly consistent associations in epidemiological studies (Kelly and Fussell 2012). Sulfate may indirectly affect health, for example, by solubilizing metals and thereby increasing their bioavailability, and by catalyzing the formation of secondary organic PM (Kelly and Fussell 2012). We identified associations with small-scale spatial variations in S and we speculate that this may reflect an influence of primary combustion from S-containing fuels and serve as a marker of within-city air pollution differences, that is, between city centers and surrounding areas.

*Associations with other elements*. None of the other elements evaluated in our analysis were significantly associated with mortality, though HRs were positive for almost all elements. There was greater heterogeneity among individual cohort effect estimates for elements other than PM_2.5_ S, though for most elements the heterogeneity was not statistically significant. There was little evidence of associations with Cu and Fe, which were mainly selected as markers of (non-tailpipe) traffic emissions. Source apportionment studies conducted elsewhere have reported that Fe is associated mostly with road dust and brake abrasion, whereas Cu is associated with tire and brake abrasion (reviewed by Viana et al. 2008). Our land use regression models had the best fit for these elements because traffic predictors were available and traffic sites were overrepresented in the measurement campaign. Therefore, we believe that the lack of an association in our study is unlikely to be attributable to exposure measurement error. In our previous analysis of the same set of cohorts, we estimated nonsignificant positive HRs for NO_2_ (1.01; 95% CI: 0.99, 1.03 per 10 μg/m^3^), NO_x_ (1.02; 95% CI: 1.00, 1.04 per 20 μg/m^3^), and PM_2.5_ aborbance (1.02; 95% CI: 0.97, 1.07 per 10^–5^/m), pollutants affected by tailpipe emissions (Beelen et al. 2014).

In single-pollutant models we found borderline statistically significant positive associations between natural-cause mortality and Si in PM_2.5_, but not Si in PM_10_, despite substantially higher Si concentrations in the coarse fraction. Source apportionment studies suggest that Si is associated primarily with crustal material in resuspended soil and road dust (Viana et al. 2008). In our previous analyses we did not find an association between mortality and coarse particles (Beelen et al. 2014).

Source apportionment studies suggest that both V and Ni are linked to crude oil and derived mainly from shipping emissions, and that K is linked to biomass burning (Viana et al. 2008). In single-pollutant models we found borderline statistically significant associations for Ni and K in PM_10_. General industry and port land use were the only predictor variables available for Ni and V in our exposure models. A specific predictor variable for wood smoke was not available (De Hoogh et al. 2013). The lack of more specific predictors in the V, Ni, and K exposure models may have limited our ability to detect element-specific mortality associations for these PM components.

*Strengths and limitations*. Our study has several strengths: large sample size, broad European coverage, adjustment for a wide range of potential (individual) confounders, and multiple elements with a high percentage of detected samples (> 75%) and good precision of measurements in all 19 cohorts (coefficient of variation < 10% for all elements, except Ni and V due to low concentration levels). An advantage compared with previous long-term studies of elemental composition that compared between-city variation and ignored within-city variation is that we could estimate spatial contrasts at much smaller spatial scales using land use regression models that were developed in a standardized way for all 19 cohorts.

We used data from measurements in 2008–2011 to develop land use regression models that were applied to addresses at baseline, mostly in the mid-1990s. Emissions of S in Europe have been reduced following a series of control measures during the last two decades (Fowler et al. 2007). However, recent studies in the Netherlands; Rome, Italy; the United Kingdom; and Vancouver, Canada, have reported that the spatial contrast of nitrogen dioxide air pollution has been stable over ≥ 10 years (Cesaroni et al. 2012; Eeftens et al. 2011; Gulliver et al. 2013; Wang et al. 2013). In addition, spatial models for black smoke and sulfur dioxide in the United Kingdom provided reasonable predictions, even going back to the 1960s, with a correlation between 1962 and 1991 concentrations of 0.53 for black smoke and 0.26 for SO_2_ (Gulliver et al. 2011). However, we cannot rule out the possibility that spatial contrasts for specific components may have been less stable over time.

We did not account for residential mobility during follow-up in the current analyses. In our previous analysis of natural-cause mortality in association with particulate matter and NO_x_ in the same cohorts, HRs for participants who moved during follow-up did not differ significantly from HRs for the complete study population, though they were slightly higher (Beelen et al. 2014).

We investigated eight *a priori*–selected elements in both the PM_2.5_ and PM_10_ fractions, so there might be some spurious associations due to multiple comparisons. In addition, correlated elements may act as surrogates for elements that are the actual causes of increased mortality. Although for almost all elements HRs were positive, the association with PM_2.5_ S clearly was the strongest. In addition, the PM_2.5_ S mortality associations were robust to adjustment for other elements, as well as particle mass. In addition, cohort-specific PM_2.5_ S HRs were almost all > 1 (Figure 3), and there was no significant heterogeneity among cohort-specific PM_2.5_ S HRs (Table 3), indicating consistency among the cohort results. The strength of the association, its consistency among cohorts, and its robustness to adjustment decrease the likelihood that the association is a spurious finding.

Differences in the accuracy of exposure estimates could bias effect estimates and standard errors for individual elements. When the measurements of two elements are correlated, part of the association between mortality and the element with more measurement error could be shifted to the estimate of association with the element with less measurement error. Accuracy of exposure estimates may depend on both the precision of the measurements and the performance of the exposure models. The eight selected elements were detected in a large majority (> 75%) of the samples. Measurement precision was best for S, Cu, and Fe but poorer for Ni and V, especially in study areas with low concentration levels (De Hoogh et al. 2013).

## Conclusion

In conclusion, long-term exposure to PM_2.5_ S was associated with natural-cause mortality. This association was robust to adjustment by other pollutants, including particle mass.

***Editor’s Note:*** *The Advance Publication of this article contained the wrong version of **Figure 2**. The correct version is included in this article.* EHP *regrets the error.*

## Supplemental Material

(2.7 MB) PDF
