# Supplementary material for: Natural-Cause Mortality and Long-Term Exposure to Particle Components: An Analysis of 19 European Cohorts within the Multi-Center ESCAPE Project
Source: Environ Health Perspect. 2015 Feb 24;123(6):525–33. doi: 10.1289/ehp.1408095 (PMC4455583; doi:10.1289/ehp.1408095)
Supplement: (2.7 MB) PDF [file ehp.1408095.s001.acco.pdf]

Environ Health Perspect
doi: 10.1289/ehp.1408095

NOTE TO READERS: *EHP* strives to ensure that all journal content is accessible to all readers. However, some figures and Supplemental Material published in *EHP* articles may not conform to 508 standards due to the complexity of the information being presented. If you need assistance accessing journal content, please contact ehp508@niehs.nih.gov. Our staff will work with you to assess and meet your accessibility needs within 3 working days.

Supplemental Material

Natural Cause Mortality and Long-Term Exposure to Particle Components: An Analysis of 19 European Cohorts within the Multi-Center ESCAPE Project

Rob Beelen, Gerard Hoek, Ole Raaschou-Nielsen, Massimo Stafoggia, Zorana Jovanovic Andersen, Gudrun Weinmayr, Barbara Hoffmann, Kathrin Wolf, Evangelia Samoli, Paul H. Fischer, Mark J. Nieuwenhuijsen, Wei W. Xun, Klea Katsouyanni, Konstantina Dimakopoulou, Alessandro Marcon, Erkki Vartiainen, Timo Lanki, Tarja Yli-Tuomi, Bente Oftedal, Per E. Schwarze, Per Nafstad, Ulf De Faire, Nancy L. Pedersen, Claes-Göran Östenson, Laura Fratiglioni, Johanna Penell, Michal Korek, Göran Pershagen, Kirsten Thorup Eriksen, Kim Overvad, Mette Sørensen, Marloes Eeftens, Petra H. Peeters, Kees Meliefste, Meng Wang, H. Bas Bueno-de-Mesquita, Dorothea Sugiri, Ursula Krämer, Joachim Heinrich, Kees de Hoogh, Timothy Key, Annette Peters, Regina Hampel, Hans Concin, Gabriele Nagel, Andrea Jaensch, Alex Ineichen, Ming-Yi Tsai, Emmanuel Schaffner, Nicole M. Probst-Hensch, Christian Schindler, Martina S. Ragettli, Alice Vilier, Françoise Clavel-Chapelon, Christophe Declercq, Fulvio Ricceri, Carlotta Sacerdote, Claudia Galassi, Enrica Migliore, Andrea Ranzi, Giulia Cesaroni, Chiara Badaloni, Francesco Forastiere, Michail Katsoulis, Antonia Trichopoulou,

Menno Keuken, Aleksandra Jedynska, Ingeborg M. Kooter, Jaakko Kukkonen, Ranjeet S. Sokhi, Paolo Vineis, and Bert Brunekreef

Table of Contents

Description of each cohort and study area

The National FINRISK Study *(FINRISK)*, Finland

The population-based Oslo Health Study (HUBRO), Norway

SNAC-K, The Swedish National study of Aging and Care in Kungsholmen (SNAC-K), Sweden

Stockholm Screening Across the Lifespan Twin study (SALT) & Twin GENE (subcohort), Sweden

Stockholm 60 year olds & IMPROVE, Sweden

Stockholm SDPP, Stockholm diabetes preventive programme (SDPP), Sweden

Danish Diet Cancer and Health study (DCH), Denmark

Study on the influence of Air pollution on Lung function, Inflammation and Aging (SALIA), Germany

The Cooperative Health Research in the Region of Augsburg (KORA), Germany

The Vorarlberg Health Monitoring and Prevention Program (VHM&PP), Austria

Swiss Cohort Study on Air Pollution and Lung and Heart Diseases in Adults (SAPALDIA), Switzerland

Italian Studies on Respiratory Disorders in Childhood and Environment (SIDRIA)

European Prospective Investigation into Cancer and Nutrition (EPIC)

EPIC- Monitoring Project on Risk Factors and chronic diseases in the Netherlands (MORGEN), The Netherlands

EPIC-Prospect, the Netherlands

European Prospective Investigation into Cancer and Nutrition (EPIC) – Oxford, UK

EPIC – Turin

EPIC – Greece

Etude Epidémiologique auprès de femmes de la Mutuelle Générale de l’Education Nationale (E3N), France

LUR model results for all study areas

Table S1. Study areas of cohorts where LUR model estimates were not available.

Table S2. PM_{10} and $PM_{2.5}$ Cu model details: Model fit and included variables.

Table S3. PM_{10} and $PM_{2.5}$ Fe model details: Model fit and included variables.

Table S4. PM_{10} and $PM_{2.5}$ K model details: Model fit and included variables.

Table S5. PM_{10} and $PM_{2.5}$ Ni model details: Model fit and included variables.

Table S6. PM_{10} and $PM_{2.5}$ S model details: Model fit and included variables.

Table S7. PM_{10} and $PM_{2.5}$ Si model details: Model fit and included variables.

Table S8. PM_{10} and $PM_{2.5}$ V model details: Model fit and included variables.

Table S9. PM_{10} and $PM_{2.5}$ Zn model details: Model fit and included variables.

Table S10. Study population characteristics at baseline for FINRISK with complete confounder information in main model 3 (N = 10,224).

Table S11. Study population characteristics at baseline for HUBRO with complete confounder information in main model 3 (N = 18,102).

Table S12. Study population characteristics at baseline for SNAC-K with complete confounder information in main model 3 (N = 2401).

Table S13. Study population characteristics at baseline for SALT / Twin gene with complete confounder information in main model 3 (N = 5473).

Table S14. Study population characteristics at baseline for 60-yr/IMPROVE with complete confounder information in main model 3 (N = 3612).

Table S15. Study population characteristics at baseline for SDPP with complete confounder information in main model 3 (N = 7408).

Table S16. Study population characteristics at baseline for DCH with complete confounder information in main model 3 (N = 35,458).

Table S17. Study population characteristics at baseline for EPIC-MORGEN with complete confounder information in main model 3 (N = 16,446).

Table S18. Study population characteristics at baseline for EPIC-PROSPECT with complete confounder information in main model 3 (N = 15,670).

Table S19. Study population characteristics at baseline for SALIA with complete confounder information in main model 3 (N = 4352).

Table S20. Study population characteristics at baseline for EPIC-Oxford with complete confounder information in main model 3 (N = 8598).

Table S21. Study population characteristics at baseline for KORA with complete confounder information in main model 3 (N = 8399).

Table S22. Study population characteristics at baseline for VHM&PP with complete confounder information in main model 3 (N = 117,824).

Table S23. Study population characteristics at baseline for SAPALDIA with complete confounder information in main model 3 (N = 1250).

Table S24. Study population characteristics at baseline for E3N with complete confounder information in main model 3 (N = 10,915).

Table S25. Study population characteristics at baseline for EPIC-Turin with complete confounder information in main model 3 (N = 7261).

Table S26. Study population characteristics at baseline for SIDRIA-Turin with complete confounder information in main model 3 (N = 5054).

Table S27. Study population characteristics at baseline for SIDRIA-Rome with complete confounder information in main model 3 (N = 9177).

Table S28. Study population characteristics at baseline for EPIC-Athens with complete confounder information in main model 3 (N = 4192).

Figure S1. Description of estimated annual mean PM_{10} elemental composition concentrations ($ng/\mu g^3$) at participant addresses in each cohort. The solid circle and bars shows the median and 25%, 75% percentile of elemental composition concentrations; the x shows the 5% and 95% percentile values.

Table S29. Correlations between PM constituents and corresponding $PM_{2.5}$ or PM_{10} mass concentrations.

Figure S2. Adjusted association between natural cause mortality and exposure to $PM_{2.5}$ Cu (per 5 ng/m^3) (using main model 3): Results from cohort-specific analyses and from random-effects meta-analyses (grey boxes indicate weights for each cohort specific estimate).

Figure S3. Adjusted association between natural cause mortality and exposure to PM_{10} Cu (per 20 ng/m^3) (using main model 3): Results from cohort-specific analyses and from random-effects meta-analyses (grey boxes indicate weights for each cohort specific estimate).

Figure S4. Adjusted association between natural cause mortality and exposure to $PM_{2.5}$ Fe (per 100 ng/m^3) (using main model 3): Results from cohort-specific analyses and from random-effects meta-analyses (grey boxes indicate weights for each cohort specific estimate).

Figure S5. Adjusted association between natural cause mortality and exposure to PM_{10} Fe (per 500 ng/m^3) (using main model 3): Results from cohort-specific analyses and from random-effects meta-analyses (grey boxes indicate weights for each cohort specific estimate).

Figure S6. Adjusted association between natural cause mortality and exposure to $PM_{2.5}$ Zn (per 10 ng/m^3) (using main model 3): Results from cohort-specific analyses and from random-effects meta-analyses (grey boxes indicate weights for each cohort specific estimate).

Figure S7. Adjusted association between natural cause mortality and exposure to PM_{10} Zn (per 20 ng/m³) (using main model 3): Results from cohort-specific analyses and from random-effects meta-analyses (grey boxes indicate weights for each cohort specific estimate).

Figure S8. Adjusted association between natural cause mortality and exposure to $PM_{2.5}$ Ni (per 1 ng/m³) (using main model 3): Results from cohort-specific analyses and from random-effects meta-analyses (grey boxes indicate weights for each cohort specific estimate).

Figure S9. Adjusted association between natural cause mortality and exposure to PM_{10} Ni (per 2 ng/m³) (using main model 3): Results from cohort-specific analyses and from random-effects meta-analyses (grey boxes indicate weights for each cohort specific estimate).

Figure S10. Adjusted association between natural cause mortality and exposure to $PM_{2.5}$ V (per 2 ng/m³) (using main model 3): Results from cohort-specific analyses and from random-effects meta-analyses (grey boxes indicate weights for each cohort specific estimate).

Figure S11. Adjusted association between natural cause mortality and exposure to PM_{10} V (per 3 ng/m³) (using main model 3): Results from cohort-specific analyses and from random-effects meta-analyses (grey boxes indicate weights for each cohort specific estimate).

Figure S12. Adjusted association between natural cause mortality and exposure to $PM_{2.5}$ Si (per 100 ng/m³) (using main model 3): Results from cohort-specific analyses and from random-effects meta-analyses (grey boxes indicate weights for each cohort specific estimate).

Figure S13. Adjusted association between natural cause mortality and exposure to PM_{10} Si (per 500 ng/m³) (using main model 3): Results from cohort-specific analyses and from random-effects meta-analyses (grey boxes indicate weights for each cohort specific estimate).

Figure S14. Adjusted association between natural cause mortality and exposure to $PM_{2.5}$ K (per 50 ng/m³) (using main model 3): Results from cohort-specific analyses and from random-effects meta-analyses (grey boxes indicate weights for each cohort specific estimate).

Figure S15. Adjusted association between natural cause mortality and exposure to PM_{10} K (per 100 ng/m³) (using main model 3): Results from cohort-specific analyses and from random-effects meta-analyses (grey boxes indicate weights for each cohort specific estimate).

Figure S16. Two-pollutant model results. All estimates shown in an individual panel are HRs for the association between the element listed as the "Main effect" at the top of the panel and natural cause mortality. Estimates from single pollutant models for the same element vary because each single pollutant model is restricted to data from cohorts in which the correlation coefficient between the main effect element and the second pollutant is <0.7, such that the single pollutant HR for $PM_{2.5}$Cu differs between the HR that is paired with NO_2 and the HR paired with $PM_{2.5}$ because of differences in the data included in each single-pollutant model.

Figure S17. Two-pollutants model results. All estimates shown in an individual panel are HRs for the association between the element listed as the "Main effect" at the top of the panel and natural cause mortality. Estimates from single pollutant models for the same element vary

because each single pollutant model is restricted to data from cohorts in which the correlation coefficient between the main effect element and the second pollutant is <0.7, such that the single pollutant HR for PM_{10} Cu differs between the HR that is paired with NO_2 and the HR paired with $PM_{2.5}$ because of differences in the data included in each single-pollutant model.

Table S30. Association between natural cause mortality and exposure to particle composition: Results from random-effects meta-analyses (HRs and 95%-CIs) for the extended confounder models. Results for main model 3 and extended confounder models are based on same number of cohorts for each exposure measure.

References

Description of each cohort and study area

The National FINRISK Study *(FINRISK)*, Finland

FINRISK surveys have been conducted every five years since 1972 to monitor the risk factor trends of chronic diseases, including cardiovascular diseases, diabetes, cancer, asthma, and allergy. For each survey, a stratified random sample has been selected from the 25-64 (74 since 1997) year old inhabitants in different regions of Finland. The ESCAPE study used FINRISK data from four surveys (1992, 1997, 2002, and 2007) and two study regions (the cities of Helsinki and Vantaa, and Turku city with its nearby municipalities). The FINRISK study protocol has been described elsewhere (Vartiainen et al. 2010).

The surveys included a self-administered questionnaire (the questions focus mainly on socioeconomic factors, medical history, health behaviour, and psychosocial factors) and a clinical examination including measurements of height, weight and blood pressure and blood sampling. The participants have been annually followed up through 31 December, 2009 (up to 17 years) for fatal and nonfatal coronary and stroke events, and total mortality. The National Hospital Discharge Register and the National Causes of Death Register were used to identify these events. These registers cover every hospitalization in Finland and every death of permanent residents in Finland, yielding in practice 100% coverage of the follow-up events (Pajunen et al. 2005; Tolonen et al. 2007). In addition, we used the drug reimbursement records from the Social Insurance Institution of Finland to identify subjects who had developed diabetes or hypertension during the follow-up period.

The population-based Oslo Health Study (HUBRO), Norway

HUBRO was designed to identify health needs and the priorities of the health sector within Oslo, to monitor the developments and trends of diseases and their associated risks, to estimate the prevalence and later the incidence of chronic diseases, to investigate the social and geographical differences in health and associated risk factors and to initiate research to further investigate the aetiology of major health problems (Søgaard et al. 2004).

HUBRO was carried out in the city of Oslo from May 2000 to September 2001. All men and women born in the following years: 1924, 1925, 1940, 1941, 1954, 1955, 1960, 1969, and 1970, who resided in Oslo on December 31, 1999, were invited to participate. 58 178 subjects were invited and 22 699 individuals (39%) participated in the study. The questionnaires covered the following topics: health and chronic diseases, family history of disease, risk factors and lifestyles, social network, education, occupation, use of health services, and use of medicine. A physical exam was performed to obtain data on blood pressure, pulse recording, and collection of venous non-fasting blood samples. HUBRO was linked to the Norwegian Cause of Death Registry including deaths up to December 31, 2010, and was also linked to the Cancer Registry of Norway including cancers up to December 31, 2009.

SNAC-K, The Swedish National study of Aging and Care in Kungsholmen (SNAC-K), Sweden

SNAC-K is an ongoing longitudinal study aiming to investigate the ageing process and identify possible preventive strategies to improve health and care in elderly adults (Lagergren et al. 2004). The study population consists of randomly sampled individuals >=60 years old and in a central area of Stockholm (Kungsholmen) between March 2001 and June 2004. The sample was stratified for age and year of assessment giving sub-cohorts with 60, 66, 72, 78, 81, 84, 87, 90,

93, 96, and 99+ year olds. Information was collected through social interviews, assessment of physical functioning, clinical examination (incl. geriatric, neurological and physical assessments) as well as cognitive assessment. At baseline, information regarding events prior to the study period was gathered. The follow-up interval is six years for the younger age cohorts, and three years for the older age cohorts (81+). During the follow-up intervals, medical events of all subjects are registered through linkage with primary care registry and hospital discharge registry (available for all subjects in Sweden). In case of death, hospital and cause of death registries provide the clinical information, and informant interviews are carried out. The same protocol as for the baseline data collection is used during the follow-up, though only concerning the follow-up period. Website of study: http://www.aldrecentrum.se/snack/index.htm. Any outcomes based on the Swedish nationwide health registries (such as the myocardial infarction and stroke registries, the cause-of-death register and the national patient register) have been used.

Stockholm Screening Across the Lifespan Twin study (SALT) & Twin GENE (subcohort), Sweden

The SALT study was set-up to screen all twins born in Sweden before 1958 for the most common complex diseases with a focus on cardiovascular diseases (Lichtenstein et al. 2006; Magunusson et al. 2013). Twin Gene is a sub-study involving establishing a biobank with DNA and serum from SALT participants. SALT is a telephone interview, which took place between 1998-2002. For the purposes of this study, only twins living in Stockholm County are included in the analyses. Information concerning birth order and weight, zygosity, contact with twin partner and family constellation, diseases, use of medication, occupation, education, life style habits, gender- and age-specific (hormone replacement therapy) and memory problems (age > 65) was collected. In Twin Gene, twins born before 1958 were contacted 2004-2008, a total number of ~2500 participants was available. Health and medication data were collected from

questionnaires. Blood sampling material was mailed to study subjects, who contacted a local health care centre for blood sampling and a health check-up. Height, weight, circumference of waist and hip, and blood pressure was measured and blood was collected. Any outcomes based on the Swedish nationwide health registries (such as the myocardial infarction and stroke registries, the cause-of-death register and the national patient register) have been used.

Stockholm 60 year olds & IMPROVE, Sweden

The 60 year olds cohort is a study aiming to identify biological and socio-economic risk factors and predictors for cardiovascular diseases (Wändell et al. 2007). Recruitment took place between August 1997 and March 1999. A random sample of every third man and woman living in Stockholm County, who was born between 1 July 1937 and 30 June 1938, was invited to the 60 year olds study. In total ~4100 subjects were included. Height, weight, BMI, Waist/Hip ratio and resting ECD, blood pressure and fasting blood samples were taken during a physical examination, while a comprehensive questionnaire was completed, including information on socioeconomic, medical and life-style factors. The study was supplemented 2003 by the IMPROVE project (an ongoing multi cohort study into genetics and CVDs). In Stockholm, IMPROVE is a sub-cohort consisting of ~500 participants from the 60 year olds cohort with inclusion criteria of having at least three risk factors for the metabolic syndrome. For IMPROVE, three follow-ups were conducted, blood and urine were collected, socio-economic data, quantitative B-mode ultra sound examination of carotid arteries and replicate B-mode ultrasound was performed, and vascular events were recorded. Any outcomes based on the Swedish nationwide health registries (such as the myocardial infarction and stroke registries, the cause-of-death register and the national patient register) have been used.

Stockholm SDPP, Stockholm diabetes preventive programme (SDPP), Sweden

The Stockholm diabetes prevention programme, a population-based prospective study, aimed at investigating the aetiology of type 2 diabetes and developing prevention strategies for type 2 diabetes (Eriksson et al. 2008). An initial survey included all men and women in the targeted age group in Stockholm County; for men in four municipalities (Värmdö, Upplands Bro, Tyresö and Sigtuna), and for women these four plus a fifth municipality (Upplands Väsby). All were screened by a questionnaire regarding presence of own diabetes and diabetes in relatives. Subjects with family history of diabetes (FHD) and randomly selected subjects without FHD, all without previously diagnosed diabetes, were invited to a health examination. This baseline study, 1992-1994 for men and 1996-1998 for women, comprised 7949 subjects, aged 35-56 years, and about 50% had FHD. In the follow-up study 8-10 years later, 2383 men (2002-2004) and 3329 women (2004-2006) participated. At the health examinations, both at baseline and follow-up, an extensive questionnaire (information on lifestyle factors, such as physical activity, dietary habits, tobacco use, alcohol consumption, health status, socioeconomic status and psychosocial conditions) was completed. Diabetes heredity was confirmed and measurements of weight, height, hip and waist circumference as well as blood pressure were performed. In addition, an oral glucose tolerance test (OGTT) was made, and blood was sampled at fasting state and 2 hour after glucose intake. Outcomes based on the Swedish nationwide health registries (such as the myocardial infarction and stroke registries, the cause-of-death register, and the national patient register) have been used.

Danish Diet Cancer and Health study (DCH), Denmark

The primary aim of the DCH study is to investigate diet and lifestyle in relation to incidence of cancer and other chronic diseases (Tjønneland et al. 2007). The study combines the collection of

questionnaire data with storing of biological specimen in order to investigate genetic susceptibility and gene-environment interactions with regard to diet, dietary compounds, and the risk of cancer, and indeogenous markers of nutritional, metabolic, and hormonal characteristics of study participants. Historical residential history of the study participants is available, which facilitate studies of air pollution and noise. The study enrolled participants in two areas, Copenhagen and Aarhus, Denmark. 160 725 individuals aged 50-64 years were invited to participate between December 1993 and May 1997. All participants were Danish-born, living in the Copenhagen or Aarhus areas and without medical history of cancer diagnosis registered in the Danish Cancer Registry at the time of invitation. Out of the 160 725 people invited, which were a random sample of all eligible individuals in the specified areas, 57 053 were enrolled. Due to the geographical limitations of the land use regression, only the almost 40 000 participants from the Copenhagen area were included in the ESCAPE analyses. On enrolment, each participant completed self-administered questionnaires (in Danish) that included questions on dietary habits, health status, family history of cancer, social factors, reproductive factors, smoking, environmental smoking, and lifestyle habits. Anthropometric measurements including blood pressure and blood samples were also obtained. The DCH cohort is followed up regularly by use of complete nationwide registers hence the loss to follow-up is virtually nil. Data on cancer incidence from the Danish Cancer Registry and data on cause-specific mortality from the Danish Mortality Registry were used.

Study on the influence of Air pollution on Lung function, Inflammation and Aging (SALIA), Germany

The SALIA study was initiated in 1985 as part of Environmental Health surveys to monitor health effects of outdoor air pollution in the heavily polluted Ruhr Area (Heinrich et al. 2012; Gehring et al. 2006). It was an element of the Clean Air Plan initiated by the Government of

North-Rhine Westphalia in Germany. The geographic regions covered were parts of Dortmund (1985, 1990), Duisburg (1990), Essen (1990), Gelsenkirchen (1986,1990) and Herne (1986). They were chosen to represent a range of polluted areas with high traffic load and steel and coal industries. Two non-industrial small towns, Dülmen (1985) and Borken (1985, 1986, 1987, 1990, 1993, 1994) were chosen as reference areas. The Research Institute for Environmental Medicine in Düsseldorf (then Medical Institute of Environmental Hygiene) coordinated the studies. The baseline investigations of SALIA were cross-sectional surveys. They were conducted on 4757 women in the local health departments in March and April between 1985 and 1994. Sampling included all women of German nationality aged 54 to 55 residing in the selected areas. Women were selected because men in these areas mainly worked in the mining industry with very high occupational exposure probably masking the effects of air pollution. Postal questionnaires were sent out and included information about airway diseases and covariates. The filled in questionnaires were checked at the day of investigation. Overall questionnaire response was 70%. Specific measurements (lung function, determination of immunological markers, and xenobiotics) were added in subgroups. All investigations were done according to standardized operating procedures.

Height and weight was measured at the day of investigation. These measurements are not available for more than 10% of all women. Therefore BMI was not included in the ESCAPE analysis, after having demonstrated that BMI did influence the results only marginally.

Follow-ups were set up to investigate the effects of outdoor air pollution and changes in pollution on respiratory health and mortality. In 1990, women investigated in 1984/1986 had a first follow-up investigation including a questionnaire and a lung function testing. A mortality follow-up of all women having participated in the baseline investigation was conducted in 2003

and in 2008 by the Institute of Epidemiology Helmholtz Munich. All surviving women were asked to participate in a questionnaire follow-up in 2006 and invited to eventually participate in a follow-up investigation. All women with lung-function available at baseline were invited to a more detailed follow-up investigation, which started in 2007.

The mortality analyses of ESCAPE use questionnaire data from the baseline investigation and the data from the mortality follow-ups. All these data were available to be included in the ESCAPE analysis.

All women with geocoded addresses at baseline were included in the analysis (4,663). Two continuous covariates were used as year of recruitment, early (1985, 1986, and 1987) and late (1990, 1993, and 1994) years. Coding was year of recruitment – 1900, recruitment before 1990 was coded as 90 in the late variable, recruitment after 1990 was coded as 87 in the early variable. No dietary covariates were available, environmental tobacco smoke was a combined variable from home and work place, occupational exposure was extreme temperature and dust. Area SES was defined as income-rate per five-digit postcode-area.

The Cooperative Health Research in the Region of Augsburg (KORA), Germany

KORA is a cohort study based on four cross-sectional surveys of a random sample of inhabitants of the Augsburg region (Holle et al. 2005). Main objectives of the baseline study were to investigate cardiovascular and other chronic diseases regarding: 1) to assess health indicators (morbidity, mortality) and health care (utilization, costs), 2) to quantify the prevalence of risk factors, and 3) to study the impact of lifestyle, metabolic and genetic factors. The follow-up studies aimed to assess also time-trends in risk factors and health over a period of seven to ten years. Two cross-sectional population-representative surveys were conducted in 1994-1995

(survey S3) and 1999-2001 (survey S4) in the city of Augsburg and two adjacent rural counties to include all inhabitants of the Augsburg region with German nationality aged 25 to 74 (n=400 000). Follow-up examinations of survey S3 and S4 participants were carried out seven to ten years later. Baseline examination included standardized interviews, physical examination, and blood sampling. All investigations were done according to standardized operating procedures.

Follow-up investigations were conducted in 2004-2005 for survey S3 and in 2006-2008 for survey S4. 2974 and 3080 of survey S3 and S4 participants attended the follow-up examinations including standardized computer-assisted interview, self-administered questionnaire, physical examination, and blood sampling. Survival was ascertained for S3 participants in 2008 through Population Registry search and is available from the time of recruitment until December 31 2007. Survival of S4 participants was ascertained through a combination of returned questionnaires and subsequent Population Registry search and is available from recruitment until December 31, 2008. Causes of death are abstracted for all deaths from the death certificates. For the ESCAPE analyses a study/baseline indicator was included instead of calendar time.

The Vorarlberg Health Monitoring and Prevention Program (VHM&PP), Austria

The VHM&PP study is a prevention program routinely performed by the Agency of Social and Preventive Medicine and covers all adults of the whole province (Ulmer et al. 2007; Ulmer et al. 1997).[14,15] It has been ongoing since 1972 and data are presently available until 2005. Recruitment and follow-up has been ongoing that means during the whole period new persons were recruited and already recruited persons came for follow-up visits. The total adult population of the state Vorarlberg is covered, with voluntary enrolment. Data are available from 1985 to 2005 at present on 185 330 persons, corresponding to about 65% participation. Their age at recruitment rangew from 18-97 years (mean=42). The screening examination takes place in the

practice of the local physicians; a self-administered questionnaire is also applied. The same protocol was applied at baseline and follow-up examinations.

A total of 132 242 geocodes were assigned exposures. 30 718 geocodes (18.85%) were omitted if: 1) they were entirely outside of the Vorarlberg state, 2) within 300m of the state boundary (lack of GIS data in neighboring countries), and 3) if their elevation was > 600m.

Swiss Cohort Study on Air Pollution and Lung and Heart Diseases in Adults (SAPALDIA), Switzerland

SAPALDIA is a multi-center study performed in eight geographic areas representing the range of environmental, meteorological, and socio-demographic conditions in Switzerland (Downs et al. 2007). A random population sample across eight geographic areas (Aarau, Basel, Davos, Geneva, Lugano, Montana, Payerne, and Wald) was obtained in 1991, with follow-ups in 2002 and 2010. The main aim of the study was to assess the effect of air pollution (outdoor and indoor) on respiratory and cardiovascular health, with a special focus on how the respiratory and cardio-vascular systems interact in this regard, and on the role of lifestyle and genetic background. In 1991, 9651 subjects, aged 18 to 60 years, were recruited via detailed interviews and more than 90% provided valid spirometry results. The follow-up in 2002 obtained health information and anthropometric data from physical re-examination with spirometry and blood sampling, blood pressure measurement, and heart rate variability in a subsample (<50yrs). The most recent follow-up (SAPALDIA 3) was in 2010. In the third assessment, study subjects were also asked in detail about chronic diseases having been diagnosed and treated since the second survey. Questionnaire domains are the following: respiratory health and disease, cardiovascular health and disease, chronic disease and relevant risk factors, women's health, allergies, medications, drug use, exposure to air pollutants, sleep apnea, and health care resources used.

SAPALDIA did not obtain information on physical activity, alcohol intake, and nutrition at baseline in 1991. Within ESCAPE, only the areas of Basel, Geneva, and Lugano were included, with PM measurements in Lugano only.

Italian Studies on Respiratory Disorders in Childhood and Environment (SIDRIA)

The SIDRIA study has been an extension of the ISAAC initiative in Italy (International Study on Asthma and Allergies in Childhood), a worldwide survey to analyse variations in prevalence of symptoms asthma, rhinitis, and atopic eczema (Cesaroni et al. 2008). A cross-sectional survey was carried out between October 1994 and March 1995 in eight centres in northern and central Italy using standardised questionnaires (response rate=94%). Parents of first and second graders from a representative sample of primary schools, and adolescents in the third year of a representative sample of junior high schools answered a self-administered questionnaire on the child's health status, as well as their personal respiratory health status and various risk factors, including education, occupation, housing conditions, smoking habits, and traffic intensity in their area of residence. The data used within ESCAPE are from the subset of parents recruited in two metropolitan areas: Rome and Turin, in the context of a project co-funded by the Ministry of Health (Programma Strategico Ambiente e Salute, Ricerca Finalizzata ex-art.12, 2006). A record linkage has been performed with the Municipal Registry Office Databases to collect the residential history of parents who were living in Rome and Turin with their children at the time of the survey. In the city of Turin the project was performed through a collaboration between SIDRIA and the regional Unit of Epidemiology (ASL TO3), in the context of the Turin Longitudinal Study, a census-based cohort study following up health outcomes of people censused in Turin since 1971. It was possible to identify ~16 000 adults.

European Prospective Investigation into Cancer and Nutrition (EPIC)

The European Prospective Investigation into Cancer and Nutrition (EPIC), which covers a large cohort of half a million men and women from 23 European centers in 10 Western European countries, was designed to study the relationship between diet and the risk of chronic diseases, particularly cancer (Riboli et al. 2002). Five of these centers were included in ESCAPE. The selection of ESCAPE participants was done centrally at Imperial College, UK using the central EPIC database.

EPIC – Monitoring Project on Risk Factors and chronic diseases in the Netherlands (MORGEN), The Netherlands

The MORGEN cohort consists of a general population sample of 10 260 men and 12 394 women aged 20–59 years from three Dutch towns (Amsterdam, Doetinchem and Maastricht) (Beulens et al. 2010). From 1993 to 1997 each year a new random sample, consisting of 6000 subjects, was examined. A total of 50 766 persons were invited to participate in the MORGEN cohort. Those who replied received two questionnaires by mail (a general questionnaire on socio-demographic factors, lifestyle and health indicators, and an FFQ and were invited to visit the local Public Health Service for a medical examination). The EPIC-MORGEN cohort and the EPIC-PROSPECT cohort have been joined to form the EPIC-NL cohort. All members of the EPIC-NL cohort are followed for changes in vital status and the occurrence of diseases by linkage with several registries, including the Municipality registry for vital status, the Dutch National Cancer registry for occurrence of cancer, the Central Bureau of Statistics registry for causes of death, and a National Hospital Discharge Diagnosis registry for occurrence of cardiovascular diseases or type 2 diabetes. Changes in some exposure status are assessed by questionnaires during follow-up. Part of the MORGEN cohort (Doetinchem participants) is re-invited every five years for a physical examination in addition to questionnaire information. The MORGEN cohort of

EPIC-NL is linked to the Dutch Cancer Registry because participants are residing in several geographical areas covered by different regional integral cancer centres.

EPIC – Prospect, the Netherlands

A total of ~17 500 healthy women, living in Utrecht and surroundings, were enrolled (Boker et al. 2001). Women were recruited from breast cancer screening participants, age 50-70 years at enrolment. The purpose of the EPIC-PROSPECT study is to assess the relation between nutrition and cancer and other chronic diseases. Baseline information was collected between 1993-1997 on the basis of two self-administered questionnaires and a medical examination. The general questionnaire contains questions on demographic characteristics, presence of chronic diseases of interest, and risk factors for chronic diseases of interest, i.e. blood pressure, serum cholesterol, reproductive history of women, family history, smoking habits, drinking of alcohol, and physical activity. Dietary intake was assessed using detailed food frequency questionnaires. A medical examination was performed including measurement of blood pressure, anthropometric measurements and taking of blood. All EPIC-PROSPECT participants are followed-up by questionnaire at 3-5 year intervals. The questionnaire collects information on changes in lifestyle habits as well as on health status. All incident and prevalent cancer cases were identified through linkage to the regional cancer registry, IKMN (Integraal Kankercentrum Midden Nederland), then from the National Cancer Registry from 2008 onwards. Vital status and cause-specific mortality information is obtained through linkage to the municipality registries and Central Buro of Statistics.

European Prospective Investigation into Cancer and Nutrition (EPIC) – Oxford, UK

The Oxford cohort was recruited from the nationwide general population in urban and rural areas throughout the United Kingdom, although a large percentage comes from Southern parts of

England and big cities such as London (Davey et al. 2003). The cohort contains 65 429 men and women over 20 years of age recruited through medical general practices or by post between 1993 and 1999, with an emphasis on vegetarians. The questionnaires gathered information on diet (FFQ), social and demographic factors, lifestyle, anthropometrics, medical history of diseases, and prevalent cancers; approximately 20 000 gave a blood sample. Participants who consented were followed-up from recuritment by "flagging" on the NHS Central Registers (NHSCRs) in England and Wales (via the Office for National Statistics), Scotland (via the General Registry Office) and Northern Ireland (via the Northern Ireland Cancer Registry) via automatic notifications. The date of each event and coding of the cancer site or type and the causes of death were recorded according to the 10th revision of the International Classification of Diseases (ICD-10). For incident cancers, tumour morphology is also coded, according to WHO ICD-O. EPIC-Oxford website: http://www.epic-oxford.org. The study population was restricted to ~8500 participants living within 10Km threshold of ESCAPE monitoring sites.

EPIC – Turin

Recruitment took place from 1993 and involved blood donors and other healthy volunteers, accruing 10 604 participants by 1998 (Guarrera et al. 2012). Co-operation with the local cancer registry and the local health authority allows for access to hospital discharge information and all newly diagnosed cancer cases. Follow-up started in 1998, including collaboration with the local cancer registry, the demographic computerized archives of the Torino area and the discharge report database for hospital patients.

EPIC – Greece

Recruitment of volunteers in EPIC-Greece started in 1994, and was completed in 1999 (Riboli et al. 2002). In total, 16 619 women and 11 953 men were recruited from Greece nationwide. Data

collection on medical and reproductive history, socio-demographic and lifestyle factors and habitual diet was performed via interview and a baseline examination that recorded measurement of anthropometric data and collection of blood samples. The follow-up of study participants was initiated in January 1997 and focused on the update of information on lifestyle factors and the health status. Due to the lack of a national cancer registry and the country-wide nature of EPIC-Greece, information is being collected through self-administered questionnaires or telephone interviews. Reported diagnoses of interest were further ascertained through consultation of medical files in hospitals and clinics all over Greece, or through the collection of death certificates from the regional death registries, in case of death. Participants that contribute to the ESCAPE analyses are residents from the Prefecture of Attica (which comprises mainly the Greater Athens Area, and hence called EPIC-Athens in the manuscript). Based on GIS availability, we included only the members if the EPIC cohort who were residents of 16 municipalities, specifically Athens, Agios Ioannis Rentis, Amaroussion, Egaleo, Galatsi, Halandri, Ilioupolis, Kalithea, Moschato, Nea Ionia, Nea Smyrni, Nikaia, Peristeri, Pireaus, Tavros, and Zografou.

Etude Epidémiologique auprès de femmes de la Mutuelle Générale de l'Education Nationale (E3N), France

E3N is a large ongoing prospective cohort consisting of 98 995 French women born between 1925 and 1950, subscribing to the health insurance plan for public education system employees, and who voluntarily enrolled in 1990-1991 (Clavel-Chapelon et al. 1996). The main objective of the study was to investigate the risk factors for breast cancer among women in particular hormonal factors and diet. This study began in 1990 when a baseline questionnaire (Q1) was sent to the 103 089 out of the 494 458 women subscribed to the health insurance plan for public education system employees women aged 40–65 years who agreed to participate. Follow up

questionnaires were sent in January 1992 (Q2) and then approximately every two years thereafter. The most recent update questionnaires was sent in June 2008 (Q9) and another one in 2010. The base population covers the whole country of France and participation was based on voluntary agreement. To date, participants have been followed for 18 years (from 1991 to 2008) with complete data available from 2005. All the questionnaires are self-administered and are sent by mail to participants in French language and returned to the study centre at IGR, Paris. Biological material was collected in 1996 on 25 000 women out of the 68 000 (who lived in communes with at least 1000 participants) invited to participate in the setting up of the biological bank. While the E3N study includes a large population in all France, exposure assessment for the ESCAPE project was available only for 4 cities: Paris, Lyon, Grenoble and Marseille. PM measurements were only done in Paris. E3N is the French component of EPIC.

LUR model results for all study areas

Table 1 shows the areas where no modeled estimates were available. The lack of any model was probably related to small within-area variability (e.g. S), poor precision of the measurements with low concentrations (Ni and V), lack of availability of predictor variables representing the major source of a component, and complexity of the study area.

Tables 2-9 show the model fit for each of the LUR models (LOOCV R^2), and which variables were included in each of the models.

Table S1. Study areas of cohorts where LUR model estimates were not available.

Elements	$PM_{2.5}$	PM_{10}
K	SALIA, Germany	HUBRO, Norway
Ni	SNAC-K, Sweden; SALT/Twin gene, Sweden; 60-y/IMPROVE, Sweden; SDPP, Sweden	SAPALDIA, Switzerland; HUBRO, Norway
S	SAPALDIA, Switzerland	SAPALDIA, Switzerland
Si	SAPALDIA, Switzerland; EPIC-Athens, Greece; HUBRO, Norway	SAPALDIA, Switzerland
V	SAPALDIA, Switzerland; KORA, Germany; VHM&PP, Austria; HUBRO, Norway	SAPALDIA, Switzerland

Table S2. PM_{10} and $PM_{2.5}$ Cu model details: Model fit and included variables.

Type	[a]LOOCV R^2	[b]Traffic (≤100m)	[c]Traffic (>100m)	[d]POP	[e]RES	[f]Industry	[g]Green	[h]Port	[i]Alt	[j]XY
FINRISK, Finland										
PM_{10}	0.61	Yes	No	Yes	No	No	No	No	No	No
$PM_{2.5}$	0.84	Yes	No	Yes	No	No	No	No	No	No
HUBRO, Norway										
PM_{10}	0.87	Yes	No	Yes	No	No	No	No	No	No
$PM_{2.5}$	0.76	Yes	Yes	Yes	No	No	No	No	No	No
SNAC-K, Sweden										
PM_{10}	0.84	Yes	Yes	No	No	No	No	No	No	No
$PM_{2.5}$	0.61	No	Yes	No	No	Yes	No	No	No	No
SALT/Twin gene, Sweden										
PM_{10}	0.84	Yes	Yes	No	No	No	No	No	No	No
$PM_{2.5}$	0.61	No	Yes	No	No	Yes	No	No	No	No
60-y/IMPROVE, Sweden										
PM_{10}	0.84	Yes	Yes	No	No	No	No	No	No	No
$PM_{2.5}$	0.61	No	Yes	No	No	Yes	No	No	No	No
SDPP, Sweden										
PM_{10}	0.84	Yes	Yes	No	No	No	No	No	No	No
$PM_{2.5}$	0.61	No	Yes	No	No	Yes	No	No	No	No
DCH, Denmark										
PM_{10}	0.91	No	Yes	No	No	No	No	No	No	No
$PM_{2.5}$	0.61	Yes	Yes	No	No	No	No	No	No	No
EPIC-MORGEN, Netherlands										
PM_{10}	0.71	Yes	Yes	No	No	No	No	Yes	No	No
$PM_{2.5}$	0.81	Yes	No	No	Yes	No	No	No	No	Yes
EPIC-PROSPECT, Netherlands										
PM_{10}	0.71	Yes	Yes	No	No	No	No	Yes	No	No
$PM_{2.5}$	0.81	Yes	No	No	Yes	No	No	No	No	Yes
SALIA, Germany										
PM_{10}	0.92	Yes	Yes	No	No	Yes	No	No	No	No
$PM_{2.5}$	0.90	Yes	Yes	No	No	Yes	No	No	No	Yes
EPIC-Oxford, UK										
PM_{10}	0.95	Yes	Yes	No	No	No	No	No	No	No
$PM_{2.5}$	0.79	Yes	No	No	Yes	No	No	No	No	No
KORA, Germany										
PM_{10}	0.71	Yes	No	No	No	Yes	No	No	No	No
$PM_{2.5}$	0.76	Yes	Yes	No	No	Yes	No	No	No	No
VHM&PP, Austria										
PM_{10}	0.95	Yes	No	No	No	Yes	Yes	No	No	No
$PM_{2.5}$	0.38	No	Yes	No	No	No	Yes	No	No	No
SAPALDIA, Switzerland										
PM_{10}	0.84	Yes	Yes	No	No	No	No	No	No	No
$PM_{2.5}$	0.83	Yes	Yes	No	No	No	No	No	No	No
E3N, France										
PM_{10}	0.48	No	Yes	No	No	Yes	No	No	No	No
$PM_{2.5}$	0.51	Yes	No	No	No	Yes	Yes	No	No	No
EPIC-Turin, Italy										
PM_{10}	0.88	Yes	No	Yes	No	No	Yes	No	No	No
$PM_{2.5}$	0.85	No	Yes	Yes	No	No	Yes	No	No	No
SIDRIA-Turin, Italy										
PM_{10}	0.88	Yes	No	Yes	No	No	Yes	No	No	No
$PM_{2.5}$	0.85	No	Yes	Yes	No	No	Yes	No	No	No
SIDRIA-Rome, Italy										
PM_{10}	0.87	Yes	Yes	No	No	No	No	No	No	No
$PM_{2.5}$	0.78	Yes	Yes	No	No	No	No	No	No	No
EPIC-Athens, Greece										
PM_{10}	0.70	Yes	Yes	No	No	No	No	No	No	No
$PM_{2.5}$	0.76	Yes	Yes	No	Yes	No	Yes	No	No	No

[a]LOOCV R^2: Leave-one out cross-validation R^2. [b]Traffic (≤100m) includes all traffic intensity and road length variables within 100m. [c]Traffic (>100m) includes all traffic intensity and road length variables beyond 100m. [d]POP indicates the number of inhabitants. [e]RES includes low and high density residential. [f]Industry indicates industry area. [g]Green includes natural green and urban green variables. [h]Port indicates port area. [i]Alt: altitude of measured sites. [j]XY: coordinate variables which indicates the trends of concentrations.

Table S3. PM_{10} and $PM_{2.5}$ Fe model details: Model fit and included variables.

Type	[a]LOOCV R^2	[b]Traffic (≤100m)	[c]Traffic (>100m)	[d]POP	[e]RES	[f]Industry	[g]Green	[h]Port	[i]Alt	[j]XY
FINRISK, Finland										
PM_{10}	0.47	Yes	Yes	Yes	No	No	No	No	No	No
$PM_{2.5}$	0.63	Yes	No	Yes	No	No	Yes	No	No	No
HUBRO, Norway										
PM_{10}	0.92	Yes	No	Yes	No	No	No	No	No	No
$PM_{2.5}$	0.82	Yes	No	No	No	No	No	No	Yes	No
SNAC-K, Sweden										
PM_{10}	0.68	Yes	Yes	No	No	No	No	No	No	No
$PM_{2.5}$	0.90	Yes	No	Yes	Yes	Yes	No	No	No	No
SALT/Twin gene, Sweden										
PM_{10}	0.68	Yes	Yes	No	No	No	No	No	No	No
$PM_{2.5}$	0.90	Yes	No	Yes	Yes	Yes	No	No	No	No
60-y/IMPROVE, Sweden										
PM_{10}	0.68	Yes	Yes	No	No	No	No	No	No	No
$PM_{2.5}$	0.90	Yes	No	Yes	Yes	Yes	No	No	No	No
SDPP, Sweden										
PM_{10}	0.68	Yes	Yes	No	No	No	No	No	No	No
$PM_{2.5}$	0.90	Yes	No	Yes	Yes	Yes	No	No	No	No
DCH, Denmark										
PM_{10}	0.92	No	Yes	No	No	No	Yes	No	No	No
$PM_{2.5}$	0.91	Yes	Yes	No	No	Yes	Yes	No	No	No
EPIC-MORGEN, Netherlands										
PM_{10}	0.70	Yes	Yes	Yes	No	No	No	No	No	No
$PM_{2.5}$	0.73	Yes	No	No	Yes	No	No	No	No	Yes
EPIC-PROSPECT, Netherlands										
PM_{10}	0.70	Yes	Yes	Yes	No	No	No	No	No	No
$PM_{2.5}$	0.73	Yes	No	No	Yes	No	No	No	No	Yes
SALIA, Germany										
PM_{10}	0.85	Yes	Yes	Yes	No	Yes	No	No	No	No
$PM_{2.5}$	0.62	Yes	Yes	No	No	Yes	No	No	No	No
EPIC-Oxford, UK										
PM_{10}	0.95	Yes	No	Yes	No	No	No	No	No	No
$PM_{2.5}$	0.92	Yes	No	Yes	No	No	No	No	No	No
KORA, Germany										
PM_{10}	0.82	Yes	Yes	No	Yes	No	No	No	No	No
$PM_{2.5}$	0.62	Yes	Yes	No	No	Yes	No	No	No	No
VHM&PP, Austria										
PM_{10}	0.62	Yes	No	No	Yes	No	No	No	No	No
$PM_{2.5}$	0.46	Yes	No	No	Yes	No	No	No	No	No
SAPALDIA, Switzerland										
PM_{10}	0.85	Yes	Yes	No	No	No	No	No	No	No
$PM_{2.5}$	0.83	Yes	Yes	No	No	No	No	No	No	No
E3N, France										
PM_{10}	0.50	No	Yes	No	No	Yes	No	No	No	No
$PM_{2.5}$	0.79	Yes	Yes	No	Yes	No	No	No	No	No
EPIC-Turin, Italy										
PM_{10}	0.86	Yes	No	Yes	No	No	Yes	No	No	No
$PM_{2.5}$	0.83	Yes	No	Yes	Yes	No	Yes	No	No	No
SIDRIA-Turin, Italy										
PM_{10}	0.86	Yes	No	Yes	No	No	Yes	No	No	No
$PM_{2.5}$	0.83	Yes	No	Yes	Yes	No	Yes	No	No	No
SIDRIA-Rome, Italy										
PM_{10}	0.82	Yes	Yes	No	No	No	No	No	No	Yes
$PM_{2.5}$	0.67	Yes	No	No	Yes	No	No	No	No	No
EPIC-Athens, Greece										
PM_{10}	0.75	Yes	Yes	No	No	Yes	Yes	No	No	No
$PM_{2.5}$	0.11	Yes	Yes	No	No	No	No	No	No	No

[a]LOOCV R^2: Leave-one out cross-validation R^2. [b]Traffic (≤100m) includes all traffic intensity and road length variables within 100m. [c]Traffic (>100m) includes all traffic intensity and road length variables beyond 100m. [d]POP indicates the number of inhabitants. [e]RES includes low and high density residential. [f]Industry indicates industry area. [g]Green includes natural green and urban green variables. [h]Port indicates port area. [i]Alt: altitude of measured sites. [j]XY: coordinate variables which indicates the trends of concentrations.

Table S4. PM_{10} and $PM_{2.5}$ K model details: Model fit and included variables.

Type	[a]LOOCV R^2	[b]Traffic (≤100m)	[c]Traffic (>100m)	[d]POP	[e]RES	[f]Industry	[g]Green	[h]Port	[i]Alt	[j]XY
FINRISK, Finland										
PM_{10}	0.24	Yes	No	No	No	No	Yes	No	No	No
$PM_{2.5}$	0.09	No	No	No	No	No	Yes	No	No	No
HUBRO, Norway										
PM_{10}	NA	No	No	No	No	No	No	No	No	No
$PM_{2.5}$	0.32	No	No	Yes	No	No	No	No	Yes	No
SNAC-K, Sweden										
PM_{10}	0.76	Yes	No	No	Yes	No	No	No	No	No
$PM_{2.5}$	0.55	Yes	No	No	Yes	No	No	No	No	No
SALT/Twin gene, Sweden										
PM_{10}	0.76	Yes	No	No	Yes	No	No	No	No	No
$PM_{2.5}$	0.55	Yes	No	No	Yes	No	No	No	No	No
60-y/IMPROVE, Sweden										
PM_{10}	0.76	Yes	No	No	Yes	No	No	No	No	No
$PM_{2.5}$	0.55	Yes	No	No	Yes	No	No	No	No	No
SDPP, Sweden										
PM_{10}	0.76	Yes	No	No	Yes	No	No	No	No	No
$PM_{2.5}$	0.55	Yes	No	No	Yes	No	No	No	No	No
DCH, Denmark										
PM_{10}	0.15	No	No	No	No	No	Yes	No	No	No
$PM_{2.5}$	0.53	No	No	No	No	Yes	Yes	No	No	No
EPIC-MORGEN, Netherlands										
PM_{10}	0.45	No	Yes	No	Yes	No	No	No	No	No
$PM_{2.5}$	0.25	No	Yes	No	No	No	No	No	No	Yes
EPIC-PROSPECT, Netherlands										
PM_{10}	0.45	No	Yes	No	Yes	No	No	No	No	No
$PM_{2.5}$	0.25	No	Yes	No	No	No	No	No	No	Yes
SALIA, Germany										
PM_{10}	0.14	No	No	No	No	Yes	No	No	No	No
$PM_{2.5}$	NA	NA	NA	NA	NA	NA	NA	NA	NA	NA
EPIC-Oxford, UK										
PM_{10}	0.56	No	No	No	Yes	No	No	No	No	No
$PM_{2.5}$	0.14	No	No	No	Yes	No	No	No	No	No
KORA, Germany										
PM_{10}	0.63	Yes	No	No	No	No	Yes	No	No	No
$PM_{2.5}$	0.38	Yes	No	No	No	No	Yes	No	Yes	No
VHM&PP, Austria										
PM_{10}	0.60	No	No	Yes	No	Yes	Yes	No	No	No
$PM_{2.5}$	0.69	No	No	Yes	No	No	Yes	No	Yes	No
SAPALDIA, Switzerland										
PM_{10}	0.67	Yes	No	No	No	No	Yes	No	No	No
$PM_{2.5}$	0.78	Yes	No	No	No	No	Yes	No	Yes	No
E3N, France										
PM_{10}	0.52	Yes	No	No	No	No	Yes	No	No	No
$PM_{2.5}$	0.31	Yes	No	No	Yes	Yes	No	No	No	No
EPIC-Turin, Italy										
PM_{10}	0.48	No	No	No	Yes	No	Yes	No	Yes	No
$PM_{2.5}$	0.11	Yes	No	No	Yes	No	No	No	No	No
SIDRIA-Turin, Italy										
PM_{10}	0.48	No	No	No	Yes	No	Yes	No	Yes	No
$PM_{2.5}$	0.11	Yes	No	No	Yes	No	No	No	No	No
SIDRIA-Rome, Italy										
PM_{10}	0.57	Yes	No	No	No	No	No	No	No	Yes
$PM_{2.5}$	0.41	Yes	No	No	No	No	No	No	No	Yes
EPIC-Athens, Greece										
PM_{10}	0.13	Yes	No	No	No	No	No	No	No	No
$PM_{2.5}$	0.51	No	Yes	No	Yes	No	Yes	No	No	No

[a]LOOCV R^2: Leave-one out cross-validation R^2. [b]Traffic (≤100m) includes all traffic intensity and road length variables within 100m. [c]Traffic (>100m) includes all traffic intensity and road length variables beyond 100m. [d]POP indicates the number of inhabitants. [e]RES includes low and high density residential. [f]Industry indicates industry area. [g]Green includes natural green and urban green variables. [h]Port indicates port area. [i]Alt: altitude of measured sites. [j]XY: coordinate variables which indicates the trends of concentrations.

Table S5. PM_{10} and $PM_{2.5}$ Ni model details: Model fit and included variables.

Type	[a]LOOCV R^2	[b]Traffic (≤100m)	[c]Traffic (>100m)	[d]POP	[e]RES	[f]Industry	[g]Green	[h]Port	[i]Alt	[j]XY
FINRISK, Finland										
PM_{10}	0.18	No	No	Yes	No	No	No	No	No	No
$PM_{2.5}$	0.08	No	No	Yes	No	No	No	No	No	No
HUBRO, Norway										
PM_{10}	NA	No	No	No	No	No	No	No	No	No
$PM_{2.5}$	0.48	No	No	No	Yes	No	No	No	No	No
SNAC-K, Sweden										
PM_{10}	0.81	Yes	Yes	No	No	Yes	Yes	No	No	No
$PM_{2.5}$	NA	No	No	No	No	No	No	No	No	No
SALT/Twin gene, Sweden										
PM_{10}	0.81	Yes	Yes	No	No	Yes	Yes	No	No	No
$PM_{2.5}$	NA	NA	NA	NA	NA	NA	NA	NA	NA	NA
60-y/IMPROVE, Sweden										
PM_{10}	0.81	Yes	Yes	No	No	Yes	Yes	No	No	No
$PM_{2.5}$	NA	NA	NA	NA	NA	NA	NA	NA	NA	NA
SDPP, Sweden										
PM_{10}	0.81	Yes	Yes	No	No	Yes	Yes	No	No	No
$PM_{2.5}$	NA	NA	NA	NA	NA	NA	NA	NA	NA	NA
DCH, Denmark										
PM_{10}	0.55	No	No	No	No	No	Yes	Yes	No	No
$PM_{2.5}$	0.05	No	Yes	No	No	No	No	No	No	No
EPIC-MORGEN, Netherlands										
PM_{10}	0.73	No	Yes	No	No	No	No	Yes	No	Yes
$PM_{2.5}$	0.72	No	No	No	No	No	No	Yes	No	Yes
EPIC-PROSPECT, Netherlands										
PM_{10}	0.73	No	Yes	No	No	No	No	Yes	No	Yes
$PM_{2.5}$	0.72	No	No	No	No	No	No	Yes	No	Yes
SALIA, Germany										
PM_{10}	0.11	No	Yes	No	No	No	No	No	No	No
$PM_{2.5}$	0.20	No	Yes	No	No	No	No	No	No	No
EPIC-Oxford, UK										
PM_{10}	0.43	Yes	No	Yes	No	No	No	No	No	No
$PM_{2.5}$	0.09	No	No	Yes	No	No	No	No	No	No
KORA, Germany										
PM_{10}	0.49	Yes	Yes	No	No	No	No	No	No	No
$PM_{2.5}$	0.32	Yes	Yes	No	No	No	No	No	Yes	No
VHM&PP, Austria										
PM_{10}	0.11	Yes	No	No	No	No	No	No	No	No
$PM_{2.5}$	0.1	No	No	No	Yes	No	No	No	No	No
SAPALDIA, Switzerland										
PM_{10}	NA	NA	NA	NA	NA	NA	NA	NA	NA	NA
$PM_{2.5}$	NA	NA	NA	NA	NA	NA	NA	NA	NA	NA
E3N, France										
PM_{10}	0.54	No	Yes	No	No	No	No	No	No	No
$PM_{2.5}$	0.12	No	Yes	No	No	No	No	No	No	No
EPIC-Turin, Italy										
PM_{10}	0.79	Yes	No	Yes	Yes	Yes	No	No	No	No
$PM_{2.5}$	0.38	No	Yes	No	No	No	No	No	No	No
SIDRIA-Turin, Italy										
PM_{10}	0.79	Yes	No	Yes	Yes	Yes	No	No	No	No
$PM_{2.5}$	0.38	No	Yes	No	No	No	No	No	No	No
SIDRIA-Rome, Italy										
PM_{10}	0.86	No	No	Yes	Yes	No	No	No	No	No
$PM_{2.5}$	0.18	No	No	Yes	No	No	No	No	No	No
EPIC-Athens, Greece										
PM_{10}	0.47	Yes	Yes	No	No	No	No	Yes	No	No
$PM_{2.5}$	0.83	No	No	Yes	No	No	No	Yes	No	No

[a]LOOCV R^2: Leave-one out cross-validation R^2. [b]Traffic (≤100m) includes all traffic intensity and road length variables within 100m. [c]Traffic (>100m) includes all traffic intensity and road length variables beyond 100m. [d]POP indicates the number of inhabitants. [e]RES includes low and high density residential.[f]Industry indicates industry area. [g]Green includes natural green and urban green variables. [h]Port indicates port area. [i]Alt: altitude of measured sites. [j]XY: coordinate variables which indicates the trends of concentrations.

Table S6. PM_{10} and $PM_{2.5}$ S model details: Model fit and included variables.

Type	[a]LOOCV R^2	[b]Traffic (≤100m)	[c]Traffic (>100m)	[d]POP	[e]RES	[f]Industry	[g]Green	[h]Port	[i]Alt	[j]XY
FINRISK, Finland										
PM_{10}	NA	NA	NA	NA	NA	NA	NA	NA	NA	NA
$PM_{2.5}$	0.23	No	No	No	No	No	Yes	No	No	No
HUBRO, Norway										
PM_{10}	0.48	Yes	No	No	Yes	No	Yes	No	No	No
$PM_{2.5}$	0.07	No	No	No	No	No	Yes	No	No	No
SNAC-K, Sweden										
PM_{10}	0.18	No	Yes	No	No	No	No	No	No	No
$PM_{2.5}$	0.24	Yes	No	No	No	No	No	No	No	No
SALT/Twin gene, Sweden										
PM_{10}	0.18	No	Yes	No	No	No	No	No	No	No
$PM_{2.5}$	0.24	Yes	No	No	No	No	No	No	No	No
60-y/IMPROVE, Sweden										
PM_{10}	0.18	No	Yes	No	No	No	No	No	No	No
$PM_{2.5}$	0.24	Yes	No	No	No	No	No	No	No	No
SDPP, Sweden										
PM_{10}	0.18	No	Yes	No	No	No	No	No	No	No
$PM_{2.5}$	0.24	Yes	No	No	No	No	No	No	No	No
DCH, Denmark										
PM_{10}	0.46	No	Yes	No	No	No	Yes	No	No	No
$PM_{2.5}$	0.61	No	No	No	No	Yes	No	Yes	No	No
EPIC-MORGEN, Netherlands										
PM_{10}	0.39	Yes	No	No	No	No	No	Yes	No	Yes
$PM_{2.5}$	0.27	No	No	Yes	No	No	No	No	No	Yes
EPIC-PROSPECT, Netherlands										
PM_{10}	0.39	Yes	No	No	No	No	No	Yes	No	Yes
$PM_{2.5}$	0.27	No	No	Yes	No	No	No	No	No	Yes
SALIA, Germany										
PM_{10}	0.55	No	No	Yes	Yes	No	No	Yes	No	No
$PM_{2.5}$	0.50	Yes	No	Yes	No	No	No	No	No	No
EPIC-Oxford, UK										
PM_{10}	0.05	No	No	No	No	No	Yes	No	No	No
$PM_{2.5}$	0.02	No	No	No	No	No	Yes	No	No	No
KORA, Germany										
PM_{10}	0.37	Yes	No	Yes	No	No	Yes	No	No	No
$PM_{2.5}$	0.70	Yes	Yes	No	No	No	Yes	No	Yes	No
VHM&PP, Austria										
PM_{10}	0.55	Yes	No	Yes	Yes	No	No	No	No	No
$PM_{2.5}$	0.53	Yes	No	No	No	No	No	No	Yes	No
SAPALDIA, Switzerland										
PM_{10}	NA	NA	NA	NA	NA	NA	NA	NA	NA	NA
$PM_{2.5}$	NA	NA	NA	NA	NA	NA	NA	NA	NA	NA
E3N, France										
PM_{10}	0.78	Yes	Yes	No	No	Yes	Yes	No	No	No
$PM_{2.5}$	0.29	Yes	Yes	No	No	No	Yes	No	No	No
EPIC-Turin, Italy										
PM_{10}	0.57	Yes	No	Yes	No	No	No	No	Yes	No
$PM_{2.5}$	0.39	No	Yes	No	Yes	No	No	No	No	No
SIDRIA-Turin, Italy										
PM_{10}	0.57	Yes	No	Yes	No	No	No	No	Yes	No
$PM_{2.5}$	0.39	No	Yes	No	Yes	No	No	No	No	No
SIDRIA-Rome, Italy										
PM_{10}	0.38	No	No	Yes	Yes	No	No	No	No	No
$PM_{2.5}$	0.33	Yes	No	Yes	No	No	No	No	No	No
EPIC-Athens, Greece										
PM_{10}	0.46	No	No	No	No	No	No	No	Yes	No
$PM_{2.5}$	0.67	No	No	Yes	Yes	No	No	No	Yes	No

[a]LOOCV R^2: Leave-one out cross-validation R^2. [b]Traffic (≤100m) includes all traffic intensity and road length variables within 100m. [c]Traffic (>100m) includes all traffic intensity and road length variables beyond 100m. [d]POP indicates the number of inhabitants. [e]RES includes low and high density residential. [f]Industry indicates industry area. [g]Green includes natural green and urban green variables. [h]Port indicates port area. [i]Alt: altitude of measured sites. [j]XY: coordinate variables which indicates the trends of concentrations.

Table S7. PM_{10} and $PM_{2.5}$ Si model details: Model fit and included variables.

Type	[a]LOOCV R^2	[b]Traffic (≤100m)	[c]Traffic (>100m)	[d]POP	[e]RES	[f]Industry	[g]Green	[h]Port	[i]Alt	[j]XY
FINRISK, Finland										
PM_{10}	0.57	Yes	No	Yes	No	No	No	No	No	No
$PM_{2.5}$	0.72	No	Yes	Yes	No	No	Yes	No	No	No
HUBRO, Norway										
PM_{10}	0.82	Yes	No	Yes	No	No	No	No	No	No
$PM_{2.5}$	NA	No	No	No	No	No	No	No	No	No
SNAC-K, Sweden										
PM_{10}	0.77	Yes	No	No	Yes	No	No	No	No	No
$PM_{2.5}$	0.65	Yes	No	No	Yes	No	No	No	No	No
SALT/Twin gene, Sweden										
PM_{10}	0.77	Yes	No	No	Yes	No	No	No	No	No
$PM_{2.5}$	0.65	Yes	No	No	Yes	No	No	No	No	No
60-y/IMPROVE, Sweden										
PM_{10}	0.77	Yes	No	No	Yes	No	No	No	No	No
$PM_{2.5}$	0.65	Yes	No	No	Yes	No	No	No	No	No
SDPP, Sweden										
PM_{10}	0.77	Yes	No	No	Yes	No	No	No	No	No
$PM_{2.5}$	0.65	Yes	No	No	Yes	No	No	No	No	No
DCH, Denmark										
PM_{10}	0.60	No	No	No	No	No	Yes	Yes	No	No
$PM_{2.5}$	0.17	No	Yes	No	No	No	No	No	No	No
EPIC-MORGEN, Netherlands										
PM_{10}	0.26	No	Yes	Yes	No	No	No	No	No	No
$PM_{2.5}$	0.39	Yes	No	No	No	No	No	No	No	Yes
EPIC-PROSPECT, Netherlands										
PM_{10}	0.26	No	Yes	Yes	No	No	No	No	No	No
$PM_{2.5}$	0.39	Yes	No	No	No	No	No	No	No	Yes
SALIA, Germany										
PM_{10}	0.70	Yes	No	Yes	No	No	No	Yes	No	No
$PM_{2.5}$	0.40	No	No	Yes	No	No	No	No	No	No
EPIC-Oxford, UK										
PM_{10}	0.49	No	No	No	Yes	No	No	No	No	No
$PM_{2.5}$	0.17	No	No	No	Yes	No	No	No	No	No
KORA, Germany										
PM_{10}	0.64	Yes	Yes	No	Yes	No	No	No	No	No
$PM_{2.5}$	0.55	Yes	Yes	No	Yes	No	No	No	No	No
VHM&PP, Austria										
PM_{10}	0.48	Yes	Yes	No	No	No	No	No	No	No
$PM_{2.5}$	0.15	Yes	No	No	No	No	Yes	No	No	No
SAPALDIA, Switzerland										
PM_{10}	NA	NA	NA	NA	NA	NA	NA	NA	NA	NA
$PM_{2.5}$	NA	NA	NA	NA	NA	NA	NA	NA	NA	NA
E3N, France										
PM_{10}	0.61	Yes	No	No	No	No	Yes	No	No	No
$PM_{2.5}$	0.50	Yes	No	Yes	No	Yes	No	No	No	No
EPIC-Turin, Italy										
PM_{10}	0.61	Yes	No	Yes	No	No	Yes	No	No	No
$PM_{2.5}$	0.54	Yes	No	Yes	No	Yes	No	No	No	No
SIDRIA-Turin, Italy										
PM_{10}	0.61	Yes	No	Yes	No	No	Yes	No	No	No
$PM_{2.5}$	0.54	Yes	No	Yes	No	Yes	No	No	No	No
SIDRIA-Rome, Italy										
PM_{10}	0.60	Yes	No	Yes	Yes	No	No	No	No	No
$PM_{2.5}$	0.43	Yes	No	Yes	No	No	No	No	No	No
EPIC-Athens, Greece										
PM_{10}	0.39	Yes	No	No	No	No	Yes	No	No	No
$PM_{2.5}$	NA	NA	NA	NA	NA	NA	NA	NA	NA	NA

[a]LOOCV R^2: Leave-one out cross-validation R^2. [b]Traffic (≤100m) includes all traffic intensity and road length variables within 100m. [c]Traffic (>100m) includes all traffic intensity and road length variables beyond 100m. [d]POP indicates the number of inhabitants. [e]RES includes low and high density residential. [f]Industry indicates industry area. [g]Green includes natural green and urban green variables. [h]Port indicates port area. [i]Alt: altitude of measured sites. [j]XY: coordinate variables which indicates the trends of concentrations.

Table S8. PM_{10} and $PM_{2.5}$ V model details: Model fit and included variables.

Type	[a]LOOCV R^2	[b]Traffic (≤100m)	[c]Traffic (>100m)	[d]POP	[e]RES	[f]Industry	[g]Green	[h]Port	[i]Alt	[j]XY
FINRISK, Finland										
PM_{10}	0.16	No	No	No	No	No	Yes	Yes	No	No
$PM_{2.5}$	0.30	No	No	Yes	No	No	No	No	No	No
HUBRO, Norway										
PM_{10}	0.83	Yes	No	Yes	Yes	No	No	No	No	No
$PM_{2.5}$	NA	No	No	No	No	No	No	No	No	No
SNAC-K, Sweden										
PM_{10}	0.70	Yes	No	No	Yes	Yes	No	No	No	No
$PM_{2.5}$	0.07	No	No	No	Yes	No	No	No	No	No
SALT/Twin gene, Sweden										
PM_{10}	0.70	Yes	No	No	Yes	Yes	No	No	No	No
$PM_{2.5}$	0.07	No	No	No	Yes	No	No	No	No	No
60-y/IMPROVE, Sweden										
PM_{10}	0.70	Yes	No	No	Yes	Yes	No	No	No	No
$PM_{2.5}$	0.07	No	No	No	Yes	No	No	No	No	No
SDPP, Sweden										
PM_{10}	0.70	Yes	No	No	Yes	Yes	No	No	No	No
$PM_{2.5}$	0.07	No	No	No	Yes	No	No	No	No	No
DCH, Denmark										
PM_{10}	0.59	No	No	No	No	No	No	Yes	No	Yes
$PM_{2.5}$	0.38	No	No	No	No	No	No	Yes	No	No
EPIC-MORGEN, Netherlands										
PM_{10}	0.67	No	No	No	No	No	No	Yes	No	Yes
$PM_{2.5}$	0.63	No	No	No	No	No	No	Yes	No	Yes
EPIC-PROSPECT, Netherlands										
PM_{10}	0.67	No	No	No	No	No	No	Yes	No	Yes
$PM_{2.5}$	0.63	No	No	No	No	No	No	Yes	No	Yes
SALIA, Germany										
PM_{10}	0.52	No	No	No	Yes	No	No	Yes	No	Yes
$PM_{2.5}$	0.48	Yes	No	No	No	No	No	Yes	No	No
EPIC-Oxford, UK										
PM_{10}	0.29	No	No	Yes	No	No	No	No	No	No
$PM_{2.5}$	0.24	No	No	No	Yes	No	No	No	No	No
KORA, Germany										
PM_{10}	0.04	No	No	No	No	No	Yes	No	No	No
$PM_{2.5}$	NA	NA	NA	NA	NA	NA	NA	NA	NA	NA
VHM&PP, Austria										
PM_{10}	0.00	No	No	No	No	No	Yes	No	No	No
$PM_{2.5}$	NA	NA	NA	NA	NA	NA	NA	NA	NA	NA
SAPALDIA, Switzerland										
PM_{10}	NA	NA	NA	NA	NA	NA	NA	NA	NA	NA
$PM_{2.5}$	NA	NA	NA	NA	NA	NA	NA	NA	NA	NA
E3N, France										
PM_{10}	0.59	Yes	No	No	No	No	Yes	No	No	No
$PM_{2.5}$	0.28	No	No	No	Yes	No	No	No	No	No
EPIC-Turin, Italy										
PM_{10}	0.62	Yes	No	No	Yes	No	No	No	No	No
$PM_{2.5}$	0.36	No	Yes	No	No	No	No	No	No	No
SIDRIA-Turin, Italy										
PM_{10}	0.62	Yes	No	No	Yes	No	No	No	No	No
$PM_{2.5}$	0.36	No	Yes	No	No	No	No	No	No	No
SIDRIA-Rome, Italy										
PM_{10}	0.68	Yes	No	No	Yes	No	No	No	Yes	No
$PM_{2.5}$	0.09	No	No	No	Yes	No	No	No	No	No
EPIC-Athens, Greece										
PM_{10}	0.72	No	No	No	No	Yes	No	Yes	No	No
$PM_{2.5}$	0.92	No	No	No	Yes	No	Yes	Yes	No	No

[a]LOOCV R^2: Leave-one out cross-validation R^2. [b]Traffic (≤100m) includes all traffic intensity and road length variables within 100m. [c]Traffic (>100m) includes all traffic intensity and road length variables beyond 100m. [d]POP indicates the number of inhabitants. [e]RES includes low and high density residential. [f]Industry indicates industry area. [g]Green includes natural green and urban green variables. [h]Port indicates port area. [i]Alt: altitude of measured sites. [j]XY: coordinate variables which indicates the trends of concentrations.

Table S9. PM_{10} and $PM_{2.5}$ Zn model details: Model fit and included variables.

Type	[a]LOOCV R^2	[b]Traffic (≤100m)	[c]Traffic (>100m)	[d]POP	[e]RES	[f]Industry	[g]Green	[h]Port	[i]Alt	[j]XY
FINRISK, Finland										
PM_{10}	NA	NA	NA	NA	NA	NA	NA	NA	NA	NA
$PM_{2.5}$	NA	NA	NA	NA	NA	NA	NA	NA	NA	NA
HUBRO, Norway										
PM_{10}	0.75	Yes	No	No	No	No	Yes	No	No	No
$PM_{2.5}$	0.70	Yes	Yes	Yes	No	No	No	No	Yes	No
SNAC-K, Sweden										
PM_{10}	0.87	Yes	No	No	No	Yes	No	No	No	No
$PM_{2.5}$	0.24	Yes	No	No	No	No	No	No	No	No
SALT/Twin gene, Sweden										
PM_{10}	0.87	Yes	No	No	No	Yes	No	No	No	No
$PM_{2.5}$	0.24	Yes	No	No	No	No	No	No	No	No
60-y/IMPROVE, Sweden										
PM_{10}	0.87	Yes	No	No	No	Yes	No	No	No	No
$PM_{2.5}$	0.24	Yes	No	No	No	No	No	No	No	No
SDPP, Sweden										
PM_{10}	0.87	Yes	No	No	No	Yes	No	No	No	No
$PM_{2.5}$	0.24	Yes	No	No	No	No	No	No	No	No
DCH, Denmark										
PM_{10}	0.72	Yes	No	No	No	No	Yes	No	No	No
$PM_{2.5}$	0.11	No	No	Yes	No	No	No	No	No	No
EPIC-MORGEN, Netherlands										
PM_{10}	0.57	No	Yes	No	No	No	No	No	No	Yes
$PM_{2.5}$	0.58	No	Yes	No	No	No	No	No	No	Yes
EPIC-PROSPECT, Netherlands										
PM_{10}	0.57	No	Yes	No	No	No	No	No	No	Yes
$PM_{2.5}$	0.58	No	Yes	No	No	No	No	No	No	Yes
SALIA, Germany										
PM_{10}	0.50	Yes	No	Yes	No	No	No	No	No	No
$PM_{2.5}$	0.58	Yes	No	No	Yes	Yes	Yes	Yes	No	No
EPIC-Oxford, UK										
PM_{10}	0.77	Yes	Yes	No	Yes	No	No	No	No	No
$PM_{2.5}$	0.63	Yes	No	Yes	No	No	No	No	No	No
KORA, Germany										
PM_{10}	0.65	Yes	Yes	No	No	No	No	No	No	No
$PM_{2.5}$	0.53	Yes	Yes	No	No	Yes	Yes	No	No	No
VHM&PP, Austria										
PM_{10}	0.79	Yes	No	Yes	Yes	No	Yes	No	No	No
$PM_{2.5}$	0.44	No	Yes	No	Yes	No	Yes	No	No	No
SAPALDIA, Switzerland										
PM_{10}	0.86	Yes	Yes	No	No	No	No	No	Yes	No
$PM_{2.5}$	0.75	Yes	No	No	Yes	No	No	No	No	No
E3N, France										
PM_{10}	0.73	Yes	Yes	No	No	No	No	No	No	No
$PM_{2.5}$	0.76	Yes	Yes	No	Yes	No	Yes	No	No	No
EPIC-Turin, Italy										
PM_{10}	0.91	Yes	No	Yes	Yes	No	Yes	No	No	No
$PM_{2.5}$	0.78	Yes	Yes	No	Yes	No	Yes	No	No	No
SIDRIA-Turin, Italy										
PM_{10}	0.91	Yes	No	Yes	Yes	No	Yes	No	No	No
$PM_{2.5}$	0.78	Yes	Yes	No	Yes	No	Yes	No	No	No
SIDRIA-Rome, Italy										
PM_{10}	0.81	Yes	No	No	Yes	No	No	No	No	No
$PM_{2.5}$	0.75	Yes	Yes	No	No	No	No	No	No	Yes
EPIC-Athens, Greece										
PM_{10}	0.73	Yes	Yes	Yes	No	No	Yes	No	Yes	No
$PM_{2.5}$	0.90	No	Yes	No	No	Yes	Yes	Yes	No	No

[a]Traffic (≤100m) includes all traffic intensity and road length variables within 100m. [b]Traffic (>100m) includes all traffic intensity and road length variables beyond 100m. [c]POP indicates the number of inhabitants. [d]RES includes low and high density residential. [e]Industry indicates industry area. [f]Green includes natural green and urban green variables. [g]Port indicates port area. [h]Alt: altitude of measured sites. [i]XY: coordinate variables which indicates the trends of concentrations.

NA: Not available.

Table S10. Study population characteristics at baseline for FINRISK with complete confounder information in main model 3 (N = 10,224).

Variable	Mean ± SD or N (%)
Age at baseline	47.9 ± 13.2
Number of cigarette equivalents/day (current)	3.8 ± 7.8
Years of regular smoking	8.6 ± 12.2)
Alcohol consumption[a]	0.9 ± 1.3
BMI (kg/m^2)	26.4 ± 4.6
Average income (3km) (in euros)	22,954 ± 5459
Gender	
Women	5501 (53.8%)
Men	4723 (46.2%)
Calendar year	
1992	2783 (27.2%)
1997	2941 (28.8%)
2002	2418 (23.7%)
2007	2082 (20.4%)
Smoking status	
Current	2638 (25.8%)
Former	2947 (28.8%)
Never	4639 (45.4%)
Marital status	
Single	1611 (15.8%)
Married/living with partner	7170 (70.1%)
Divorced/separated	1100 (10.8%)
Widowed	343 (3.4%)
Educational level	
Low	3167 (31.0%)
Medium	5291 (51.8%)
High	1766 (17.3%)
Environmental tobacco smoke at work and/or home	
No	8322 (81.4%)
Yes	1902 (18.6%)
Intake of fruit	
Daily	6783 (66.3%)
Weekly	2639 (25.8%)
Seldom	592 (5.8%)
Never	210 (2.1%)
Intake of vegetables	
Daily	6973 (68.2%)
Weekly	2550 (24.9%)
Seldom	488 (4.8%)
Never	213 (2.1%)
Occupational class	
Blue collar	1528 (14.9%)
White collar	5435 (53.2%)
Students/housewives/retired/ unemployed	3261 (31.9%)
Employment status	
Employed/Self-employed	7073 (69.2%)
Unemployed	621 (6.1%)
Homemaker/housewife	347 (3.4%)
Retired	2183 (21.4%)
Area indicator	
Helsinki and Vantaa	4935 (48.3%)
Turku area	5289 (51.7%)

[a]Number of glasses of alcoholic drink during last week.

Table S11. Study population characteristics at baseline for HUBRO with complete confounder information in main model 3 (N = 18,102).

Variable	Mean ± SD or N (%)
Age at baseline	48.3 ± 15.2
Number of cigarette equivalents/day (lifetime average)	6.8 ± 8.4
Years of regular smoking	11.6 ± 14.4
BMI (kg/m^2)	25.7 ± 4.1
Unemployment rate (municipality level) (%)	1.8 ± 0.7
Gender	
Women	10,150 (56.1%)
Men	7952 (43.9%)
Calendar year	
2000	7870 (43.5%)
2001	10,232 (56.5%)
Smoking status	
Current	4720 (26.1%)
Former	5072 (28.0%)
Never	8310 (45.9%)
Alcohol consumption	
Weekly	9195 (50.8%)
Occasionally	7313 (40.4%)
Never/not past year	1594 (8.8%)
Intake of fruit	
Daily	7228 (39.9%)
Weekly	8825 (48.8%)
Rarely	2049 (11.3%)
Intake of vegetables	
Daily	2620 (14.5%)
Weekly	12,424 (68.6%)
Rarely	3058 (16.9%)
Marital status	
Single	5620 (31.0%)
Married/living with partner	9010 (49.8%)
Divorced/separated	2452 (13.5%)
Widowed	1020 (5.6%)
Educational level	
Low	3177 (17.6%)
Medium	6555 (36.2%)
High	8370 (46.2%)
Employment status	
Employed	11,976 (66.2%)
Self-employed	1308 (7.2%)
Unemployed	2063 (11.4%)
Retired or stay at home	2755 (15.2%)

Table S12. Study population characteristics at baseline for SNAC-K with complete confounder information in main model 3 (N = 2401).

Variable	Mean ± SD or N (%)
Age at baseline	70.3 ± 8.1
Number of cigarette equivalents/day (lifetime average)	7.1 ± 9.5
Years of regular smoking	9.8 ± 15.2
BMI (kg/m^2)	26.0 ± 4.1
Average income (neighborhood) (in Swedish kronor)	352,638 ± 26,928
Gender	
Women	1441 (60.0%)
Men	960 (40.0%)
Calendar year	
2001	512 (21.3%)
2002	691 (28.8%)
2003	798 (33.2%)
2004	400 (16.7%)
Smoking status	
Current	378 (15.7%)
Former	960 (40.0%)
Never	1063 (44.3%)
Marital status	
Single	305 (12.7%)
Married/living with partner	1301 (54.2%)
Divorced/separated	364 (15.2%)
Widowed	431 (18.0%)
Educational level	
Low	509 (21.2%)
Medium	1039 (43.3%)
High	853 (35.5%)
Environmental tobacco smoke at work	
No	810 (33.7%)
Yes	1591 (66.3%)
Environmental tobacco smoke at home	
No	1094 (45.6%)
Yes	1307 (54.4%)
Occupation class	
Blue collar	387 (16.1%)
White collar	2014 (83.9%)
Employment status	
Other	1714 (71.4%)
Employed	687 (28.6%)
Alcohol consumption	
Daily	524 (21.8%)
Weekly	643 (26.8%)
Seldom	1060 (44.1%)
Never	174 (7.2%)

Table S13. Study population characteristics at baseline for SALT / Twin gene with complete confounder information in main model 3 (N = 5473).

Variable	Mean ± SD or N (%)
Age at baseline	58.0 ± 9.9
Number of cigarette equivalents/day (lifetime average)	8.5 ± 9.7
Years of regular smoking	16.7 ± 17.3
BMI (kg/m^2)	28.6 ± 4.1
Gender	
Women	3050 (55.7%)
Men	2423 (44.3%)
Calendar year	
1998	262 (4.8%)
1999	1467 (26.8%)
2000	1410 (25.8%)
2001	1177 (21.5%)
2002	1157 (21.1%)
Smoking status	
Current	1295 (23.7%)
Former	2059 (37.6%)
Never	2119 (38.7%)
Marital status	
Single	784 (14.3%)
Married/living with partner	3723 (68.0%)
Divorced/separated	612 (11.2%)
Widowed	354 (6.5%)
Educational level	
Low	1179 (21.5%)
Medium	2360 (43.1%)
High	1934 (35.3%)
Individual level socioeconomic status	
Low	1643 (30.0%)
Medium	2842 (51.9%)
High	988 (18.1%)
Mean income (municipality level) (in Swedish kronor)	
Quartile 1	1528 (27.9%)
Quartile 2	2366 (43.2%)
Quartile 3	221 (4.0%)
Quartile 4	1358 (24.8%)

Table S14. Study population characteristics at baseline for 60-yr/IMPROVE with complete confounder information in main model 3 (N = 3612).

Variable	Mean ± SD or N (%)
Age at baseline	60.4 ± 0.1
Number of cigarette equivalents/day (lifetime average)	8.0 ± 9.1
Years of regular smoking	15.2 ± 16.4
Alcohol consumption (g/day)	8.9 ± 9.7
BMI (kg/m^2)	26.8 ± 4.2
Average income (municipality) (in Swedish kronor)	290,838 ± 46,103
Gender	
Women	1897 (52.5%)
Men	1715 (47.5%)
Calendar year	
1997	757 (21.0%)
1998	2772 (76.7%)
1999	83 (2.3%)
Smoking status	
Current	761 (21.0%)
Former	1371 (38.0%)
Never	1480 (41.0%)
Environmental tobacco smoke at work and/or home	
No	1898 (52.5%)
Yes	1714 (47.5%)
Marital status	
Single	161 (4.5%)
Married/living with partner	2587 (71.6%)
Divorced/separated	617 (17.1%)
Widowed	247 (6.8%)
Educational level	
Low	995 (27.5%)
Medium	1596 (44.2%)
High	1021 (28.3%)
Occupation class	
Blue collar	820 (22.7%)
Low white collar	1977 (54.7%)
High white	815 (22.6%)
Employment status	
Employed/Self-employed	1857 (51.4%)
Unemployed	351 (9.7%)
Homemaker/housewife	276 (7.6%)
Retired	1128 (31.2%)
Intake of fruit	
Daily	2318 (64.2%)
Weekly	1015 (28.1%)
Seldom/never	279 (1.7%)
Intake of vegetables	
Daily	476 (13.2%)
Weekly	3085 (85.4%)
Seldom/never	51 (1.4%)

Table S15. Study population characteristics at baseline for SDPP with complete confounder information in main model 3 (N = 7408).

Variable	Mean ± SD or N (%)
Age at baseline	47.1 ± 5.0
Number of cigarette equivalents/day (lifetime average)	8.5 ± 8.8
Years of regular smoking	12.3 ± 12.4
Alcohol consumption[a]	1.3 ± 1.9
BMI (kg/m^2)	25.6 ± 4.0
Average income (municipality) (in Swedish kronor)	277,069 ± 18,711
Gender	
Women	4570 (61.7%)
Men	2838 (38.3%)
Calendar year	
1992	292 (3.9%)
1993	1741 (23.5%)
1994	805 (10.9%)
1995	1815 (24.5%)
1996	2378 (32.1%)
1997	377 (5.1%)
Smoking status	
Current	1928 (26.0%)
Former	2711 (36.6%)
Never	2769 (37.4%)
Marital status	
Single/living alone	1217 (16.4%)
Married/living with partner	6191 (83.6%)
Educational level	
Low	1892 (25.5%)
Medium	3321 (44.8%)
High	2195 (29.6%)
Occupation class	
Worker/blue collar	2451 (33.1%)
White collar	4957 (66.9%)
Employment status	
Not employed	606 (8.2%)
Employed	6802 (91.8%)
Intake of fruit	
Daily/weekly	6845 (92.4%)
Seldom	482 (6.5%)
Never	81 (1.1%)

[a]Number of glasses of alcoholic drink per day.

Table S16. Study population characteristics at baseline for DCH with complete confounder information in main model 3 (N = 35,458).

Variable	Mean ± SD or N (%)
Age at baseline	56.7 ± 4.4
Number of cigarette equivalents/day (current)	6.3 ± 10.4
Years of regular smoking	18.7 ± 17.1
Intake of fruit (g/day)	183.2 ± 151.2
Intake of vegetables (g/day)	175.9 ± 99.2
Alcohol consumption (g/day)	21.7 ± 22.8
BMI (kg/m^2)	26.0 ± 4.1
Average income (municipality) (in 100,000 Danish kroner)	1.9 ± 0.4
Gender	
Women	19,171 (54.1%)
Men	16,287 (45.9%)
Calendar year	
1993	86 (0.2%)
1994	3712 (10.5%)
1995	11,034 (31.1%)
1996	14,726 (41.5%)
1997	5900 (16.6%)
Smoking status	
Current	12,737 (35.9%)
Former	9851 (27.8%)
Never	12,870 (36.3%)
Marital status	
Single	2317 (6.5%)
Married/living with partner	24,544 (69.2%)
Divorced/separated	6539 (18.4%)
Widowed	2058 (5.8%)
Educational level	
Low	10,490 (29.6%)
Medium	16,844 (47.5%)
High	8124 (22.9%)
Environmental tobacco smoke at work and/or home	
No	12,654 (35.7%)
Yes	22,804 (64.3%)
Employment status	
Not employed	7073 (19.9%)
Employed	28,385 (80.1%)

Table S17. Study population characteristics at baseline for EPIC-MORGEN with complete confounder information in main model 3 (N = 16,446).

**Variable**	**Mean ± SD or N (%)**
Age at baseline	43.9 ± 10.9
Number of cigarette equivalents/day (lifetime average)	10.4 ± 11.1
Years of regular smoking	14.3 ± 13.7
Intake of fruit (g/day)	171.9 ± 129.2
Intake of vegetables (g/day)	126.6 ± 51.8
Alcohol consumption (g/day)	12.7 ± 18.0
BMI (kg/m^2)	25.2 ± 4.0
Percentage of people with low income (neighborhood)	41.6 ± 7.4
Gender	
Women	8946 (54.4%)
Men	7500 (45.6%)
Calendar year	
1993	3566 (21.7%)
1994	2948 (17.9%)
1995	3568 (21.7%)
1996	3365 (20.5%)
1997	2999 (18.2%)
Smoking status	
Current	5923 (36.0%)
Former	4762 (29.0%)
Never	5761 (35.0%)
Marital status	
Single	3669 (22.3%)
Married/living with partner	11,118 (67.6%)
Divorced/separated	1311 (8.0%)
Widowed	348 (2.1%)
Educational level	
Low	1954 (11.9%)
Medium	10,752 (65.4%)
High	3740 (22.7%)

Table S18. Study population characteristics at baseline for EPIC-PROSPECT with complete confounder information in main model 3 (N = 15,670).

Variable	Mean ± SD or N (%)
Age at baseline	57.7 ± 6.0
Number of cigarette equivalents/day (lifetime average)	5.7 ± 7.4
Years of regular smoking	15.2 ± 16.5
Intake of fruit (g/day)	231.6 ± 139.2
Intake of vegetables (g/day)	136.3 ± 52.5
Alcohol consumption (g/day)	9.0 ± 12.4
BMI (kg/m^2)	25.5 ± 4.1
Percentage of people with low income (municipality)	35.9 ± 2.7
Percentage of people with low income (neighborhood)	35.8 ± 7.2
Gender	
Women	15,670 (100%)
Men	0 (0%)
Calendar year	
1993	1354 (8.6%)
1994	4071 (26.0%)
1995	4023 (25.7%)
1996	4102 (26.2%)
1997	2120 (13.5%)
Smoking status	
Current	3454 (22.0%)
Former	5166 (33.0%)
Never	7050 (45.0%)
Marital status	
Single	888 (5.7%)
Married/living with partner	12,046 (76.9%)
Divorced/separated	1252 (8.0%)
Widowed	1484 (9.5%)
Educational level	
Low	3478 (22.2%)
Medium	9685 (61.8%)
High	2507 (16.0%)

Table S19. Study population characteristics at baseline for SALIA with complete confounder information in main model 3 (N = 4352).

Variable	Mean ± SD or N (%)
Age at baseline	54.5 ± 0.6
Number of cigarette equivalents/day (current)	2.6 ± 6.6
Years of regular smoking	4.4 ± 10.5
Average income (postal code area) (EUR)	973.6 ± 69.1
Gender	
Women	4352 (100%)
Men	0 (0%)
Calendar year	
1985-1987	1667 (38.3%)
1990-1994	2685 (61.7%)
Smoking status	
Current	729 (16.7%)
Former	379 (8.7%)
Never	3244 (74.5%)
Educational level	
Low	1255 (28.8%)
Medium	2094 (48.1%)
High	1003 (23.0%)
Environmental tobacco smoke at work and/or home	
No	2141 (49.2%)
Yes	2211 (50.8%)
Occupational exposure to dust	
No	3923 (90.1%)
Yes	429 (9.9%)

Table S20. Study population characteristics at baseline for EPIC-Oxford with complete confounder information in main model 3 (N = 8598).

Variable	Mean ± SD or N (%)
Age at baseline	46.0 ± 13.1
Number of cigarette equivalents/day (lifetime average)	5.5 ± 8.8
Years of regular smoking	7.3 ± 11.5
Intake of fruit (g/day)	253.6 ± 216.5
Intake of vegetables (g/day)	270.4 ± 156.2
Alcohol consumption (g/day)	10.0 ± 12.3
BMI (kg/m^2)	24.3 ± 4.3
Carstairs index 2001 (continuous)[a]	-0.9 ± 2.8
Gender	
Women	6,463 (75.2%)
Men	2,135 (24.8%)
Calendar year	
1993	14 (0.2%)
1994	1566 (18.2%)
1995	1906 (22.2%)
1996	2574 (29.9%)
1997	1636 (19.0%)
1998-2001	902 (10.4%)
Smoking status	
Current	1041 (12.1%)
Former	2369 (27.6%)
Never	5188 (60.3%)
Marital status	
Single	1721 (20.0%)
Married/living with partner	5781 (67.2%)
Divorced/separated	809 (8.4%)
Widowed	287 (3.3%)
Educational level	
Low	2916 (33.9%)
Medium	1959 (22.8%)
High	3723 (43.3%)
Employment status	
Employed/self-employed	6575 (76.5%)
Unemployed	222 (2.6%)
Stay at home	809 (9.4%)
Retired	992 (11.5%)

[a]The Carstairs index is an index of deprivation and based on four census indicators: low social class, lack of car ownership, overcrowding and male unemployment.

Table S21. Study population characteristics at baseline for KORA with complete confounder information in main model 3 (N = 8399).

Variable	Mean ± SD or N (%)
Age at baseline	49.5 ± 13.8
Number of cigarette equivalents/day (lifetime average)	9.2 ± 13.3
Years of regular smoking	12.0 ± 14.2
Alcohol consumption (g/day)	16.3 ± 22.3
BMI (kg/m^2)	27.2 ± 4.6
Percentage of people with low income (5km grid)	28.2 ± 18.4
Gender	
Women	4270 (50.8%)
Men	4129 (49.2%)
Calendar year	
1994-1995	4299 (51.2%)
1999-2001	4100 (48.8%)
Smoking status	
Current	2183 (26.0%)
Former	2546 (30.3%)
Never	3670 (43.7%)
Marital status	
Single	872 (10.4%)
Married/living with partner	6356 (75.7%)
Divorced/separated	635 (7.6%)
Widowed	536 (6.4%)
Educational level	
Low	1059 (12.6%)
Medium	6270 (74.7%)
High	1070 (12.7%)
Environmental tobacco smoke at home	
No	6390 (76.1%)
Yes	2009 (23.9%)
Environmental tobacco smoke at work	
No	6328 (75.3%)
Yes	2071 (24.7%)
Employment status	
Employed/self-employed	4894 (58.3%)
Unemployed	273 (3.3%)
Stay at home	1170 (13.9%)
Retired	2062 (24.6%)
Intake of fruit	
Daily	4995 (59.5%)
Weekly	2547 (30.3%)
Seldom/never	857 (10.2%)
Intake of vegetables	
Daily	3953 (47.1%)
Weekly	3821 (45.5%)
Seldom/never	625 (7.4%)

Table S22. Study population characteristics at baseline for VHM&PP with complete confounder information in main model 3 (N = 117,824).

Variable	Mean ± SD or N (%)
Age at baseline	41.9 ± 14.9
BMI (kg/m^2)	24.8 ± 4.3
Average income (municipality) (EUR)	25,119 ± 1273
Gender	
Women	66,042 (56.1%)
Men	51,782 (43.9%)
Calendar year	
1985-1989	58,490 (49.6%)
1990-1994	26,393 (22.4%)
1995-1999	18,414 (15.6%)
2000-2005	14,527 (12.3%)
Smoking status	
Current	28,255 (24.0%)
Former	7233 (6.1%)
Never	82,336 (69.9%)
Marital status	
Single	20,134 (17.1%)
Married/living with partner	80,572 (68.4%)
Divorced/separated	8962 (7.6%)
Widowed	8156 (6.9%)
Occupational class	
White collar	66,348 (56.3%)
Blue collar	40,961 (34.8%)
Others (mainly self-employed)	10,515 (8.9%)
Employment status	
Employed/self-employed	81,705 (69.3%)
Unemployed	4126 (3.5%)
Retired	31,993 (27.2%)

Table S23. Study population characteristics at baseline for SAPALDIA with complete confounder information in main model 3 (N = 1250).[a]

Variable	Mean ± SD or N (%)
Age at baseline	42.0 ± 11.9
Number of cigarette equivalents/day (lifetime average)	11.1 ± 14.4
Years of regular smoking	11.1 ± 13.0
BMI (kg/m^2)	23.8 ± 3.9
Average educational level (neighborhood)[a]	3.1 ± 0.2
Gender	
Women	696 (55.7%)
Men	554 (44.3%)
Calendar year	
1991	1250 (100%)
Smoking status	
Current	463 (37.0%)
Former	229 (18.3%)
Never	558 (44.6%)
Marital status	
Single	404 (32.3%)
Married/living with partner	726 (58.1%)
Divorced/separated	89 (7.1%)
Widowed	31 (2.5%)
Educational level	
Low	141 (11.3%)
Medium	909 (72.7%)
High	200 (16.0%)
Environmental tobacco smoke at home	
No	1057 (84.6%)
Yes	193 (15.4%)
Environmental tobacco smoke at work	
No	1116 (89.3%)
Yes	134 (10.7%)
Employment status	
Employed	1010 (80.8%)
Unemployed	31 (2.5%)
Stay at home or retired	206 (16.5%)

[a]Average of a 7-categories (1-7) level of education variable, calculated for participants living within the same neighborhood zone.

Table S24. Study population characteristics at baseline for E3N with complete confounder information in main model 3 (N = 10,915).

Variable	Mean ± SD or N (%)
Age at baseline	53.0 ± 6.8
Intake of fruit (g/day)	236.2 ± 162.5
Intake of vegetables (g/day)	238.6 ± 126.5
Alcohol consumption (g/day)	12.4 ± 15.4
BMI (kg/m^2)	22.8 ± 3.3
Unemployment rate (regional scale)	9.0 ± 0.4
Gender	
Women	10,915 (100%)
Men	0 (0%)
Calendar year	
1993	8201 (75.1%)
1994	1743 (16.0%)
1995	676 (6.2%)
1996	295 (2.7%)
Smoking status	
Current	1840 (16.9%)
Former	3807 (34.9%)
Never	5268 (48.3%)
Educational level	
Low	520 (4.8%)
Medium	610 (5.6%)
High	9785 (89.6%)

Table S25. Study population characteristics at baseline for EPIC-Turin with complete confounder information in main model 3 (N = 7261).

Variable	Mean ± SD or N (%)
Age at baseline	50.4 ± 7.5
Number of cigarette equivalents/day (lifetime average)	7.2 ± 8.2
Years of regular smoking	17.6 ± 16.3
Intake of fruit (g/day)	318.2 ± 182.2
Intake of vegetables (g/day)	181.8 ± 100.2
Alcohol consumption (g/day)	18.1 ± 20.3
BMI (kg/m^2)	25.3 ± 3.8
Gender	
Women	3,461 (47.7%)
Men	3,800 (52.3%)
Calendar year	
1993	457 (6.3%)
1994	1264 (17.4%)
1995	2318 (31.9%)
1996	1541 (21.2%)
1997	1432 (19.7%)
1998	251 (3.5%)
Smoking status	
Current	1830 (25.2%)
Former	2339 (32.2%)
Never	3092 (42.6%)
Marital status	
Not married (single, widowed, separated, divorced)	1045 (14.4%)
Married	6216 (85.6%)
Educational level	
Low	3168 (43.6%)
Medium	3104 (42.7%)
High	989 (13.6%)
Deprivation index (quintiles) (census block)	
I (less deprived)	1876 (25.8%)
II	1659 (22.8%)
III	1350 (18.6%)
IV	1411 (19.4%)
V (more deprived)	965 (13.3%)

Table S26. Study population characteristics at baseline for SIDRIA-Turin with complete confounder information in main model 3 (N = 5054).

Variable	Mean ± SD or N (%)
Age at baseline	44.2 ± 6.2
Number of cigarette equivalents/day (current)	9.3 ± 10.2
Years of regular smoking	11.3 ± 10.6
Gender	
Women	2620 (51.8%)
Men	2434 (48.2%)
Calendar year	
1999	5054 (100%)
Smoking status	
Current	2110 (41.7%)
Former	1047 (20.7%)
Never	1897 (37.5%)
Marital status	
Married/living with	4820 (95.4%)
Single/divorced/separated/ widowed	234 (4.6%)
Educational level	
Low	884 (17.5%)
Medium	3604 (71.3%)
High	566 (11.2%)
Environmental tobacco smoke at home	
No	4389 (86.8%)
Yes	665 (13.2%)
Occupational class	
Blue collar	2120 (41.9%)
White collar	1529 (30.3%)
Other	1405 (27.8%)
Employment status	
Employed	3649 (72.2%)
Unemployed	351 (6.9%)
Homemaker/housewife/retired	1054 (20.9%)
Deprivation index (quintiles) (census block)	
I (less deprived)	878 (17.4%)
II	1049 (20.8%)
III	931 (18.4%)
IV	1097 (21.7%)
V (more deprived)	1099 (21.7%)

Table S27. Study population characteristics at baseline for SIDRIA-Rome with complete confounder information in main model 3 (N = 9177).

Variable	Mean ± SD or N (%)
Age at baseline	44.3 ± 6.0
Number of cigarette equivalents/day (current)	10.1 ± 10.5
Years of regular smoking	11.7 ± 10.4
Gender	
Women	4848 (52.8%)
Men	4329 (47.2%)
Calendar year	
1999	9177 (100%)
Smoking status	
Current	3898 (42.5%)
Former	2106 (22.9%)
Never	3173 (34.6%)
Marital status	
Married/living with partner	9177 (100%)
Educational level	
Low	4121 (44.9%)
Medium	3681 (40.1%)
High	1375 (15.0%)
Occupation class	
Non-manual	4783 (52.1%)
Manual	1179 (12.8%)
Worker unspecified	521 (5.7%)
Unemployed	392 (4.3%)
Housewife	2302 (25.1%)
Index of socioeconomic position (census block)	
1 (=High)	1703 (18.6%)
2	1684 (18.4%)
3	1667 (18.2%)
4	1797 (19.6%)
5 (=Low)	2326 (25.3%)

Table S28. Study population characteristics at baseline for EPIC-Athens with complete confounder information in main model 3 (N = 4192).

Variable	Mean ± SD or N (%)
Age at baseline	49.4 ± 11.7
Number of cigarette equivalents/day (lifetime average)	1.7 ± 15.0
Years of regular smoking	10.8 ± 13.1
Intake of fruit (g/day)	402.6 ± 258.2
Intake of vegetables (g/day)	609.5 ± 288.6
Alcohol consumption (g/day)	9.2 ± 14.5
BMI (kg/m^2)	27.5 ± 4.5
Gender	
Women	2306 (55.0%)
Men	1886 (45.0%)
Calendar year	
1994	1582 (37.7%)
1995	1100 (26.2%)
1996	367 (8.8%)
1997	457 (10.9%)
1998	278 (6.6%)
1999	408 (9.7%)
Smoking status	
Current	1707 (40.7%)
Former	830 (19.8%)
Never	1655 (39.5%)
Marital status	
Single	394 (9.4%)
Married/living with partner	3270 (78.0%)
Divorced/separated	266 (6.3%)
Widowed	262 (6.3%)
Educational level	
Low	990 (23.6%)
Medium	1753 (41.8%)
High	1449 (34.6%)
Occupation class	
Blue collar	493 (11.8%)
White collar	1990 (47.5%)
Other	1709 (40.8%)
Employment status	
Employed/self-employed	2804 (66.9%)
Unemployed	28 (0.7%)
Homemaker/housewife	669 (16.0%)
Retired	691 (16.5%)
Educational level (municipality level)	
1: Low (primary)	214 (5.1%)
2: Medium (secondary)	3277 (78.2%)
3: High (higher)	701 (16.7%)

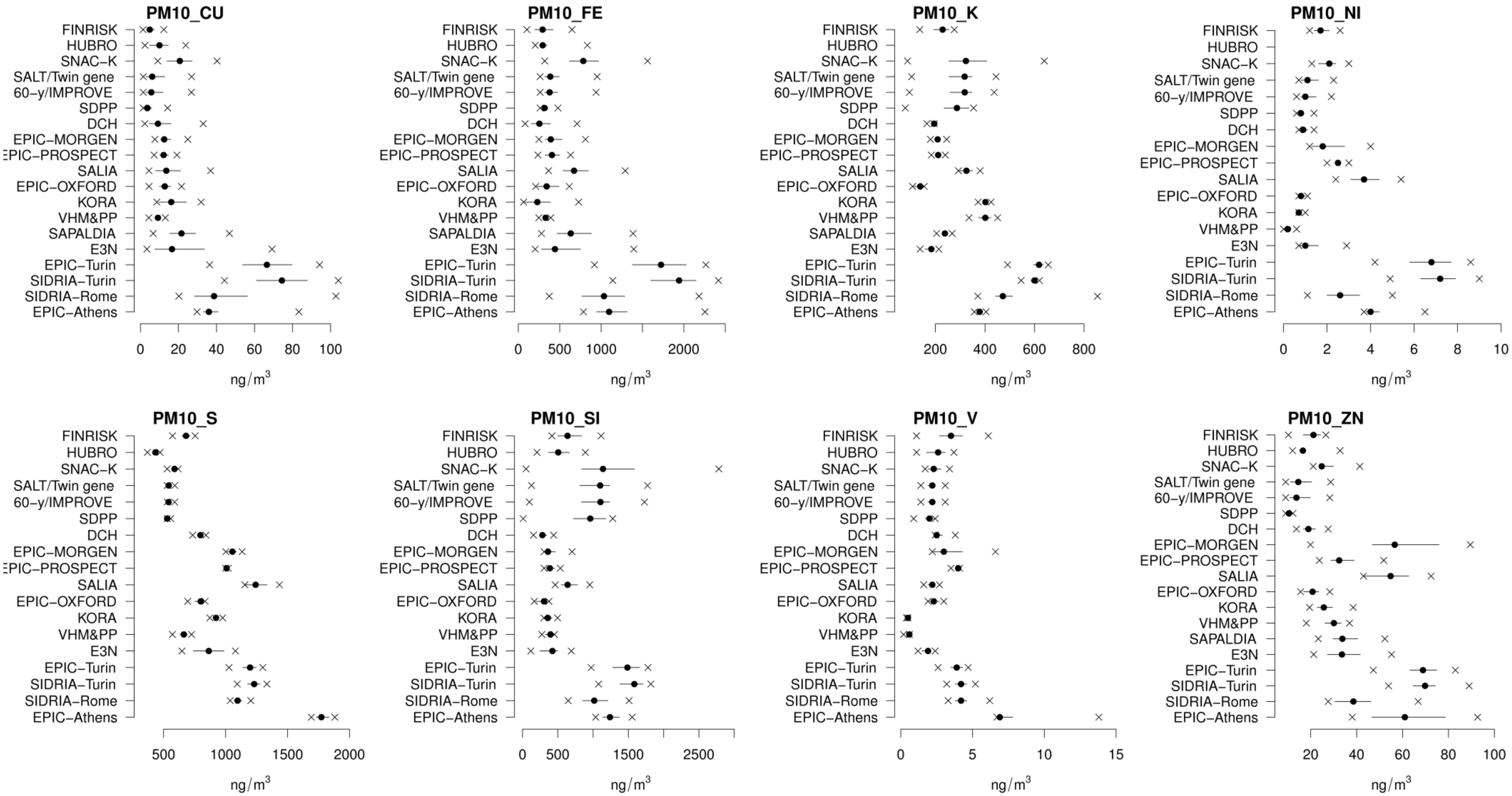

Figure S1. Description of estimated annual mean PM_{10} elemental composition concentrations (ng/μg^3) at participant addresses in each cohort. The solid circle and bars shows the median and 25%, 75% percentile of elemental composition concentrations; the x shows the 5% and 95% percentile values.

Table S29. Correlations between PM constituents and corresponding $PM_{2.5}$ or PM_{10} mass concentrations.

Cohort	Cu: $PM_{2.5}$	Cu: PM_{10}	Fe: $PM_{2.5}$	Fe: PM_{10}	K: $PM_{2.5}$	K: PM_{10}	Ni: $PM_{2.5}$	Ni: PM_{10}	S: $PM_{2.5}$	S: PM_{10}	Si: $PM_{2.5}$	Si: PM_{10}	V: $PM_{2.5}$	V: PM_{10}	Zn: $PM_{2.5}$	Zn: PM_{10}
FINRISK, Finland	0.31	0.74	0.48	0.71	0.97	0.71	0.17	0.74	0.86	0.79	0.19	0.81	0.09	0.61	0.81	0.60
HUBRO, Norway	0.67	0.55	0.28	0.43	0.37	0.80	0.35	NA	0.56	0.52	NA	0.83	NA	0.63	NA	0.62
SNAC-K, Sweden	0.57	0.12	0.51	0.85	0.44	1.00	NA	-0.18	0.65	0.28	0.59	1.00	0.41	0.70	0.59	0.83
SALT/Twin gene, Sweden	0.48	0.22	0.45	0.71	0.36	1.00	NA	0.00	0.51	0.30	0.50	1.00	0.34	0.73	0.45	0.64
60-yr/IMPROVE, Sweden	0.54	0.31	0.51	0.76	0.41	1.00	NA	0.11	0.53	0.37	0.56	1.00	0.36	0.76	0.49	0.68
SDPP, Sweden	0.41	0.17	0.45	0.45	0.26	1.00	NA	0.17	0.26	0.23	0.22	1.00	0.32	0.93	0.27	0.45
DCH, Denmark	0.54	0.72	0.56	0.75	0.43	0.30	0.58	0.72	0.52	0.68	0.58	0.76	0.57	0.67	0.66	0.69
EPIC-MORGEN, Netherlands	0.69	0.76	0.48	0.92	0.48	0.78	0.02	0.85	0.55	0.41	0.54	0.94	0.00	0.79	0.40	-0.37
EPIC-PROSPECT, Netherlands	0.72	0.47	0.47	0.81	0.44	0.74	-0.08	0.56	0.27	0.29	0.32	0.84	-0.06	0.20	0.42	0.60
SALIA, Germany	0.86	0.79	0.72	0.93	NA	0.49	0.76	0.89	0.70	0.72	0.32	0.47	0.45	0.11	0.61	0.43
EPIC-Oxford, UK	0.67	0.53	0.68	0.45	0.61	0.37	0.77	0.45	0.37	0.16	0.61	0.37	0.61	0.39	0.69	0.53
KORA, Germany	0.31	0.47	0.44	0.57	0.42	0.35	0.41	0.38	0.39	0.46	0.50	0.47	NA	0.00	0.64	0.66
VHM&PP, Austria	0.58	0.61	0.31	0.47	0.87	0.63	0.39	0.57	0.58	0.83	0.05	0.37	NA	0.03	0.33	0.69
SAPALDIA, Switzerland	0.73	0.67	0.85	0.91	0.89	0.95	NA	NA	NA	NA	NA	NA	NA	NA	0.57	0.91
E3N, France	0.76	0.56	0.65	0.56	0.43	0.50	0.66	0.61	0.51	0.48	0.11	0.58	0.24	0.31	0.65	0.74
EPIC-Turin, Italy	0.57	0.86	0.73	0.99	0.11	0.31	0.51	0.87	0.60	0.73	0.37	0.83	0.48	0.47	0.65	0.76
SIDRIA-Turin, Italy	0.32	0.69	0.59	0.82	-0.03	0.30	0.30	0.73	0.33	0.69	0.19	0.65	0.33	0.48	0.40	0.65
SIDRIA-Rome, Italy	0.71	0.74	0.70	0.76	0.50	0.60	0.24	0.14	0.67	0.18	0.53	0.38	0.08	0.56	0.63	0.72
EPIC-Athens, Greece	0.36	0.23	0.56	0.22	0.27	0.30	0.20	0.03	0.36	0.17	NA	0.09	0.20	-0.02	0.31	0.09
Median	0.57	0.56	0.51	0.75	0.43	0.63	0.37	0.56	0.53	0.44	0.44	0.79	0.33	0.52	0.58	0.65
IQR	0.26	0.34	0.21	0.32	0.13	0.52	0.35	0.59	0.22	0.41	0.33	0.45	0.29	0.47	0.24	0.14

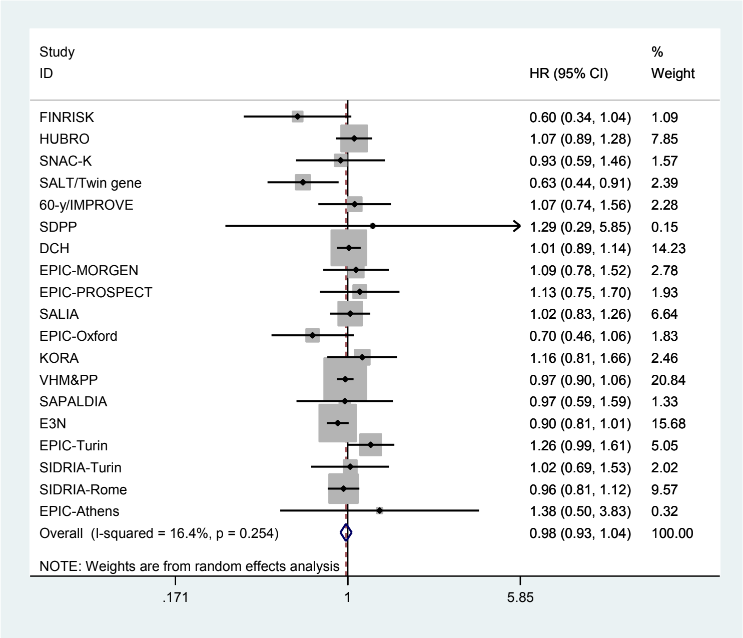

Figure S2. Adjusted association between natural cause mortality and exposure to $PM_{2.5}$ Cu (per 5 ng/m³) (using main model 3): Results from cohort-specific analyses and from random-effects meta-analyses (grey boxes indicate weights for each cohort specific estimate).

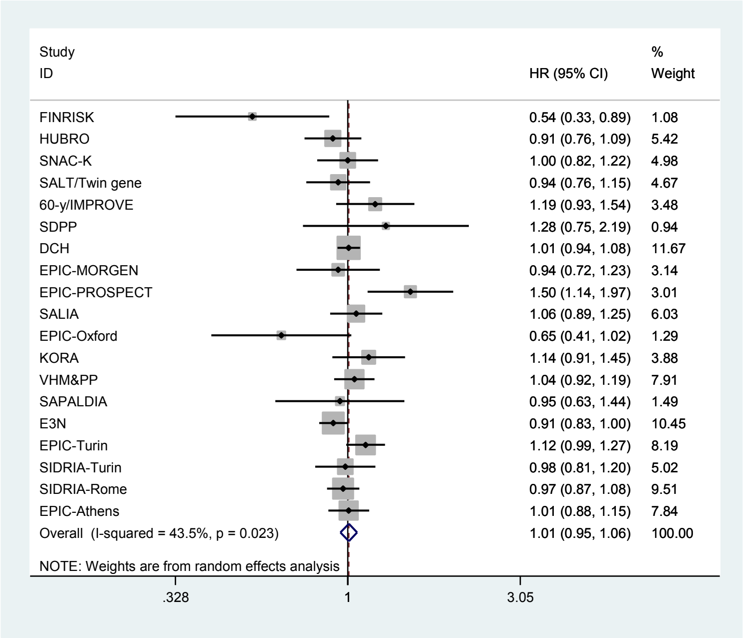

Figure S3. Adjusted association between natural cause mortality and exposure to PM_{10} Cu (per 20 ng/m³) (using main model 3): Results from cohort-specific analyses and from random-effects meta-analyses (grey boxes indicate weights for each cohort specific estimate).

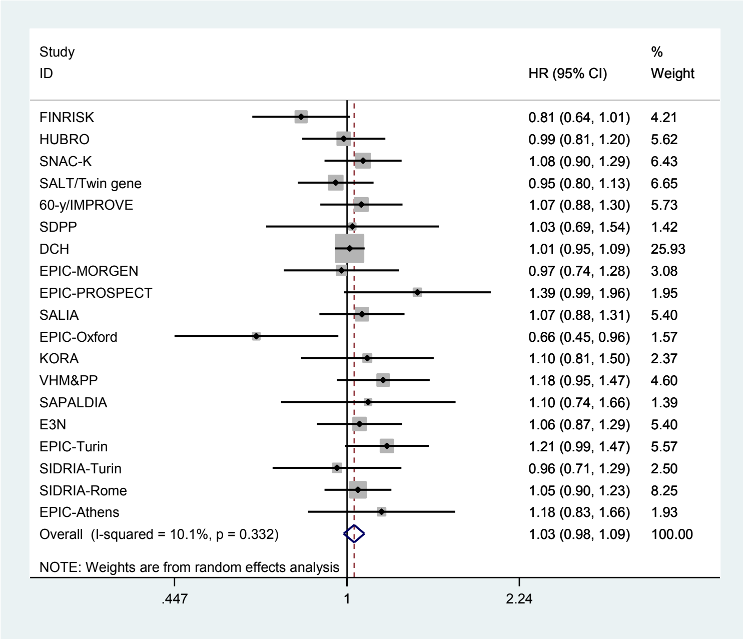

Figure S4. Adjusted association between natural cause mortality and exposure to $PM_{2.5}$ Fe (per 100 ng/m³) (using main model 3): Results from cohort-specific analyses and from random-effects meta-analyses (grey boxes indicate weights for each cohort specific estimate).

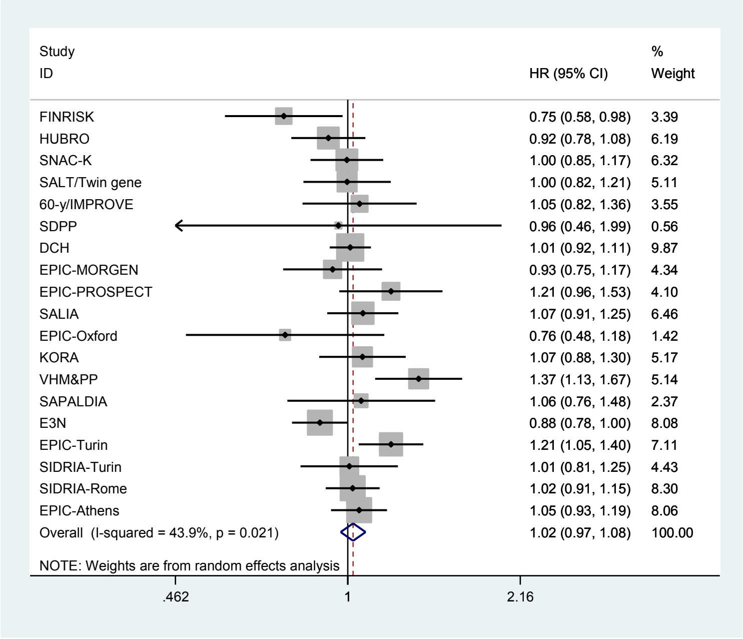

Figure S5. Adjusted association between natural cause mortality and exposure to PM_{10} Fe (per 500 ng/m^3) (using main model 3): Results from cohort-specific analyses and from random-effects meta-analyses (grey boxes indicate weights for each cohort specific estimate).

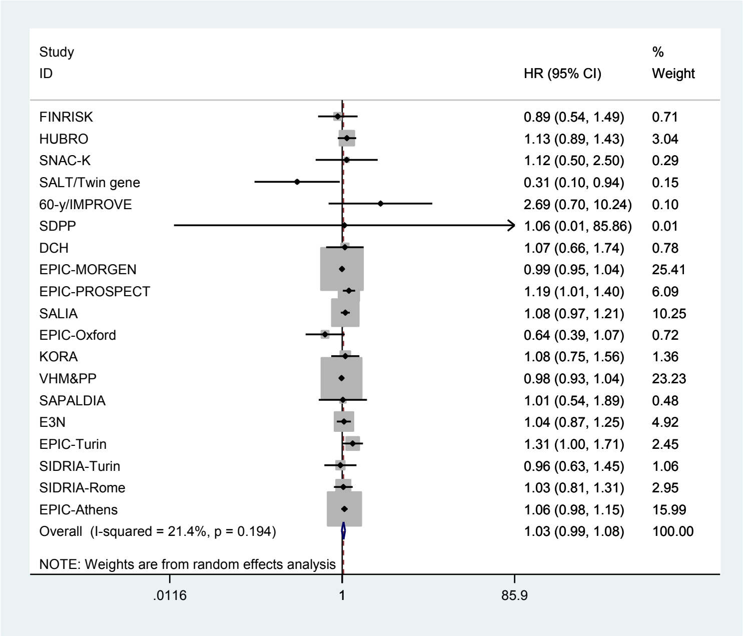

Figure S6. Adjusted association between natural cause mortality and exposure to $PM_{2.5}$ Zn (per 10 ng/m³) (using main model 3): Results from cohort-specific analyses and from random-effects meta-analyses (grey boxes indicate weights for each cohort specific estimate).

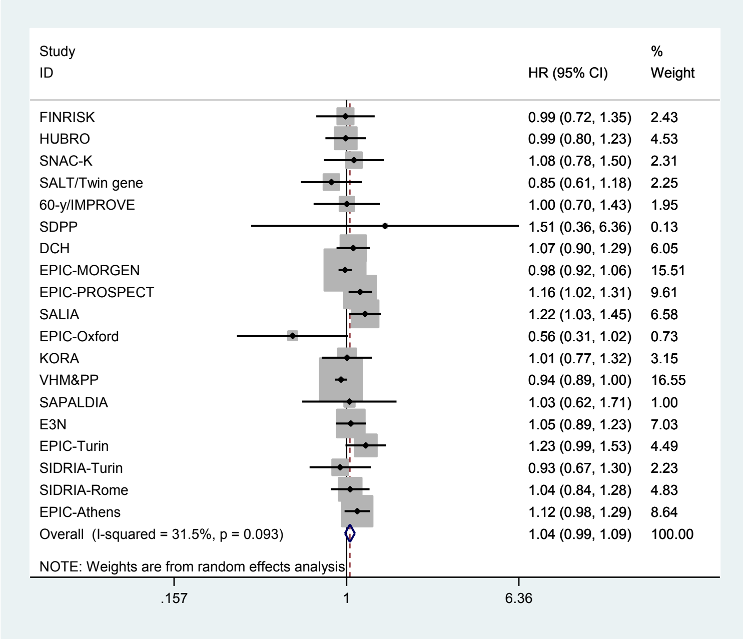

Figure S7. Adjusted association between natural cause mortality and exposure to PM_{10} Zn (per 20 ng/m³) (using main model 3): Results from cohort-specific analyses and from random-effects meta-analyses (grey boxes indicate weights for each cohort specific estimate).

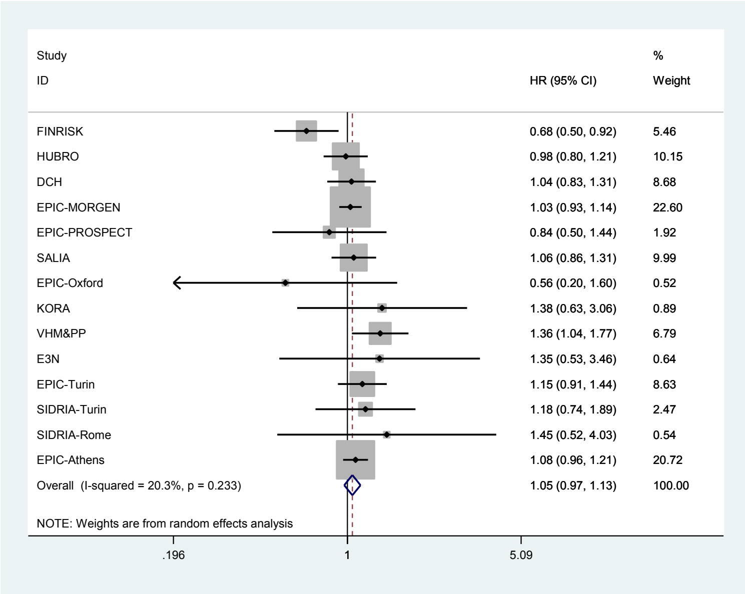

Figure S8. Adjusted association between natural cause mortality and exposure to $PM_{2.5}$ Ni (per 1 ng/m³) (using main model 3): Results from cohort-specific analyses and from random-effects meta-analyses (grey boxes indicate weights for each cohort specific estimate).

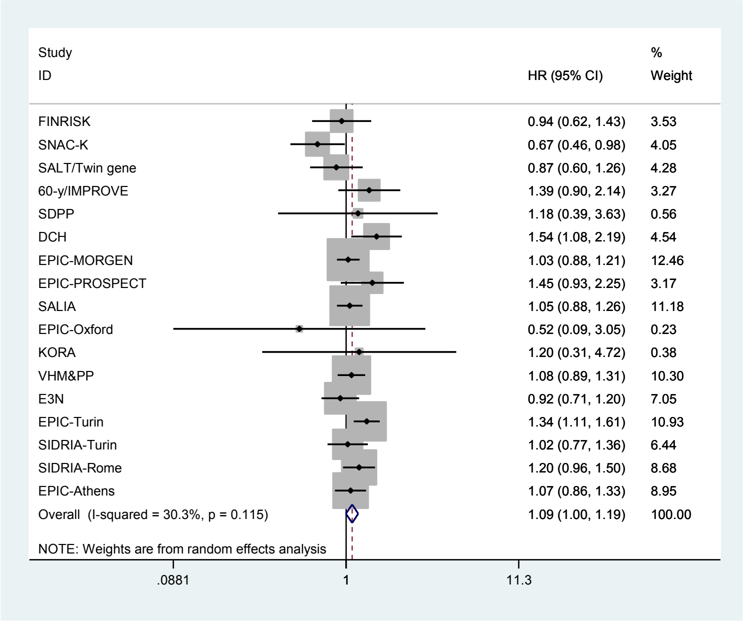

Figure S9. Adjusted association between natural cause mortality and exposure to PM_{10} Ni (per 2 ng/m³) (using main model 3): Results from cohort-specific analyses and from random-effects meta-analyses (grey boxes indicate weights for each cohort specific estimate).

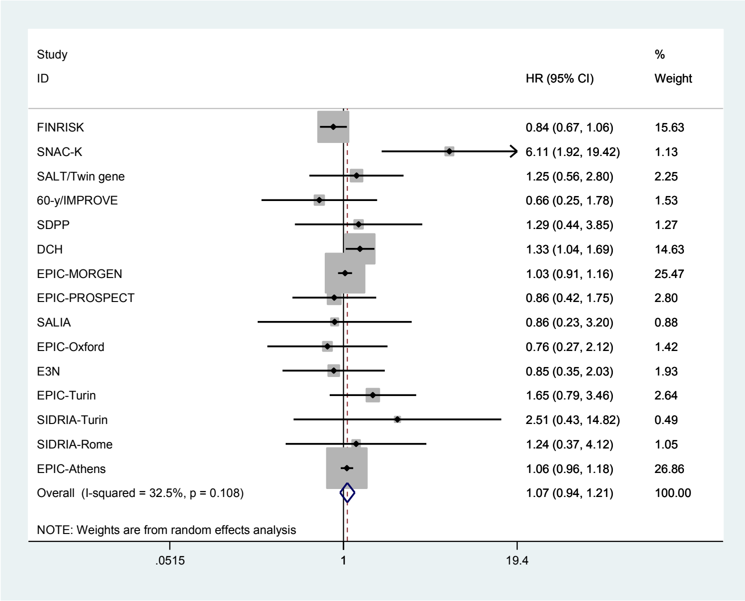

Figure S10. Adjusted association between natural cause mortality and exposure to $PM_{2.5}$ V (per 2 ng/m³) (using main model 3): Results from cohort-specific analyses and from random-effects meta-analyses (grey boxes indicate weights for each cohort specific estimate).

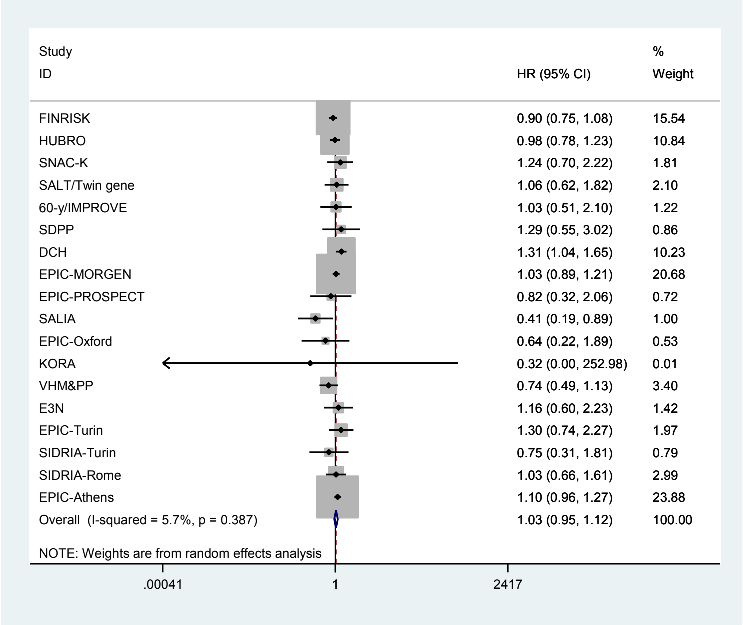

Figure S11. Adjusted association between natural cause mortality and exposure to PM_{10} V (per 3 ng/m³) (using main model 3): Results from cohort-specific analyses and from random-effects meta-analyses (grey boxes indicate weights for each cohort specific estimate).

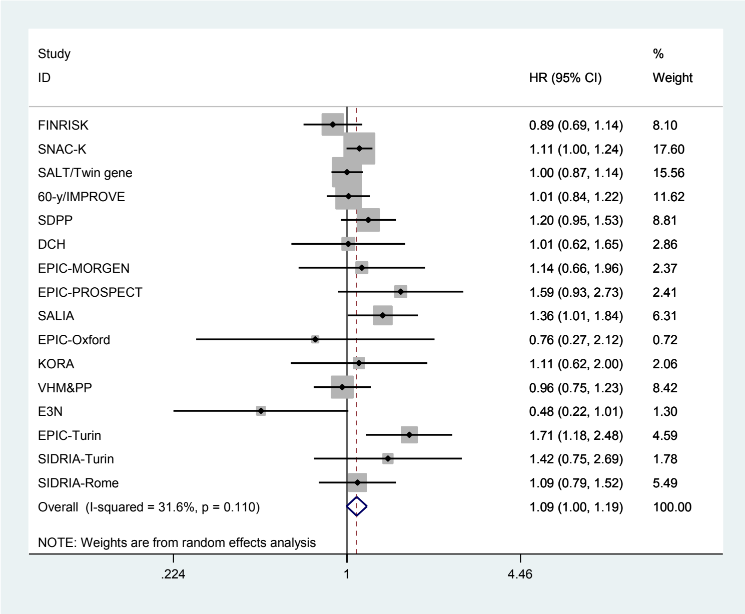

Figure S12. Adjusted association between natural cause mortality and exposure to $PM_{2.5}$ Si (per 100 ng/m³) (using main model 3): Results from cohort-specific analyses and from random-effects meta-analyses (grey boxes indicate weights for each cohort specific estimate).

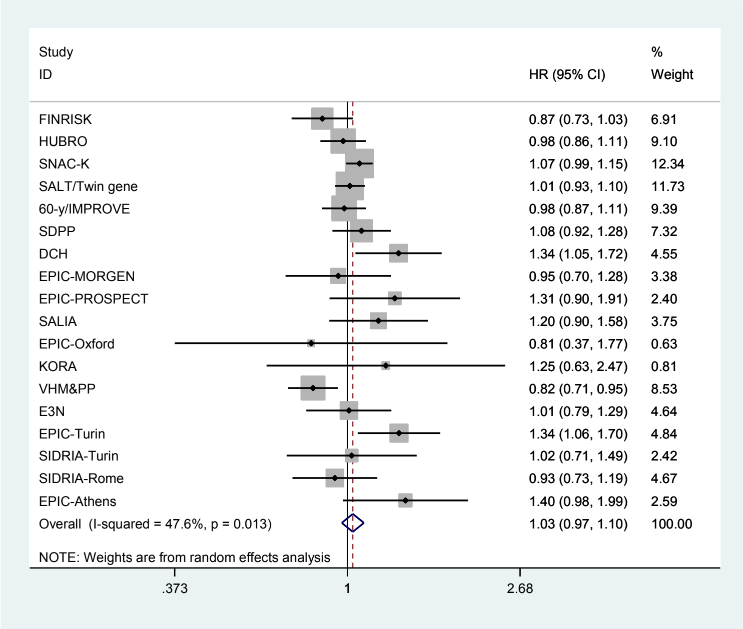

Figure S13. Adjusted association between natural cause mortality and exposure to PM_{10} Si (per 500 ng/m³) (using main model 3): Results from cohort-specific analyses and from random-effects meta-analyses (grey boxes indicate weights for each cohort specific estimate).

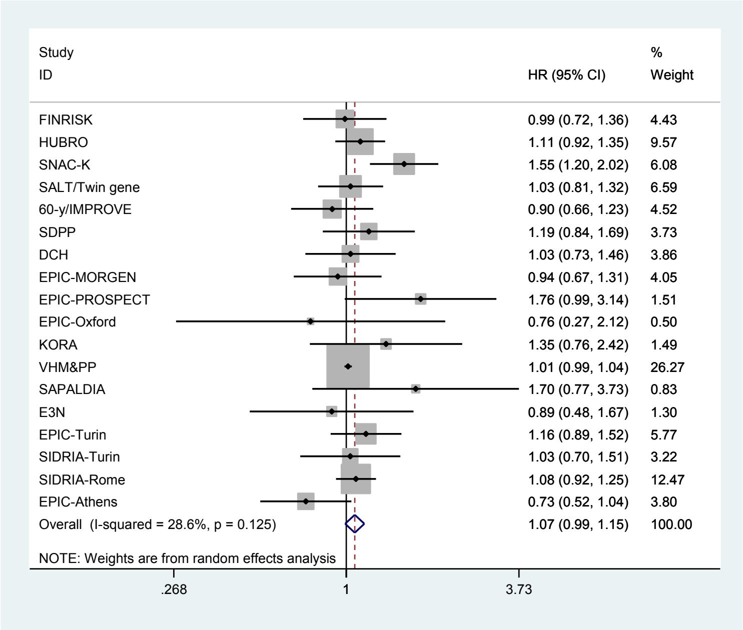

Figure S14. Adjusted association between natural cause mortality and exposure to $PM_{2.5}$ K (per 50 ng/m³) (using main model 3): Results from cohort-specific analyses and from random-effects meta-analyses (grey boxes indicate weights for each cohort specific estimate).

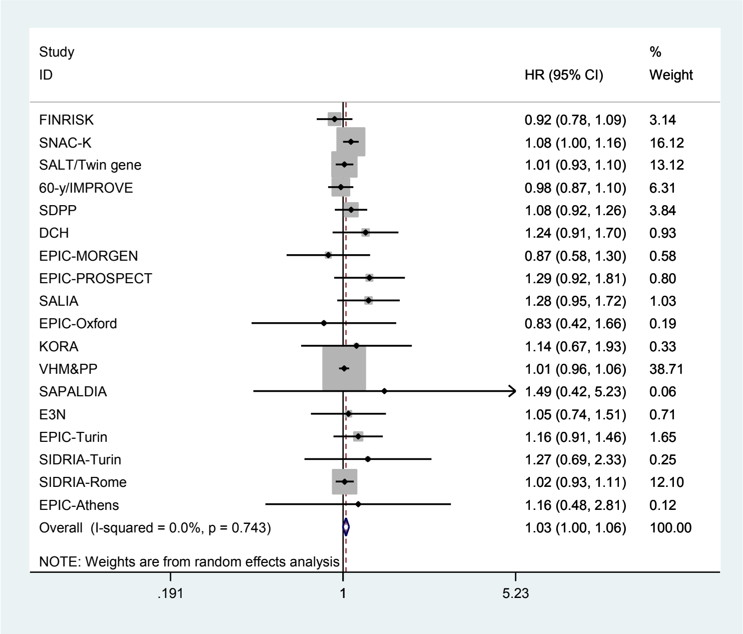

Figure S15. Adjusted association between natural cause mortality and exposure to PM_{10} K (per 100 ng/m³) (using main model 3): Results from cohort-specific analyses and from random-effects meta-analyses (grey boxes indicate weights for each cohort specific estimate).

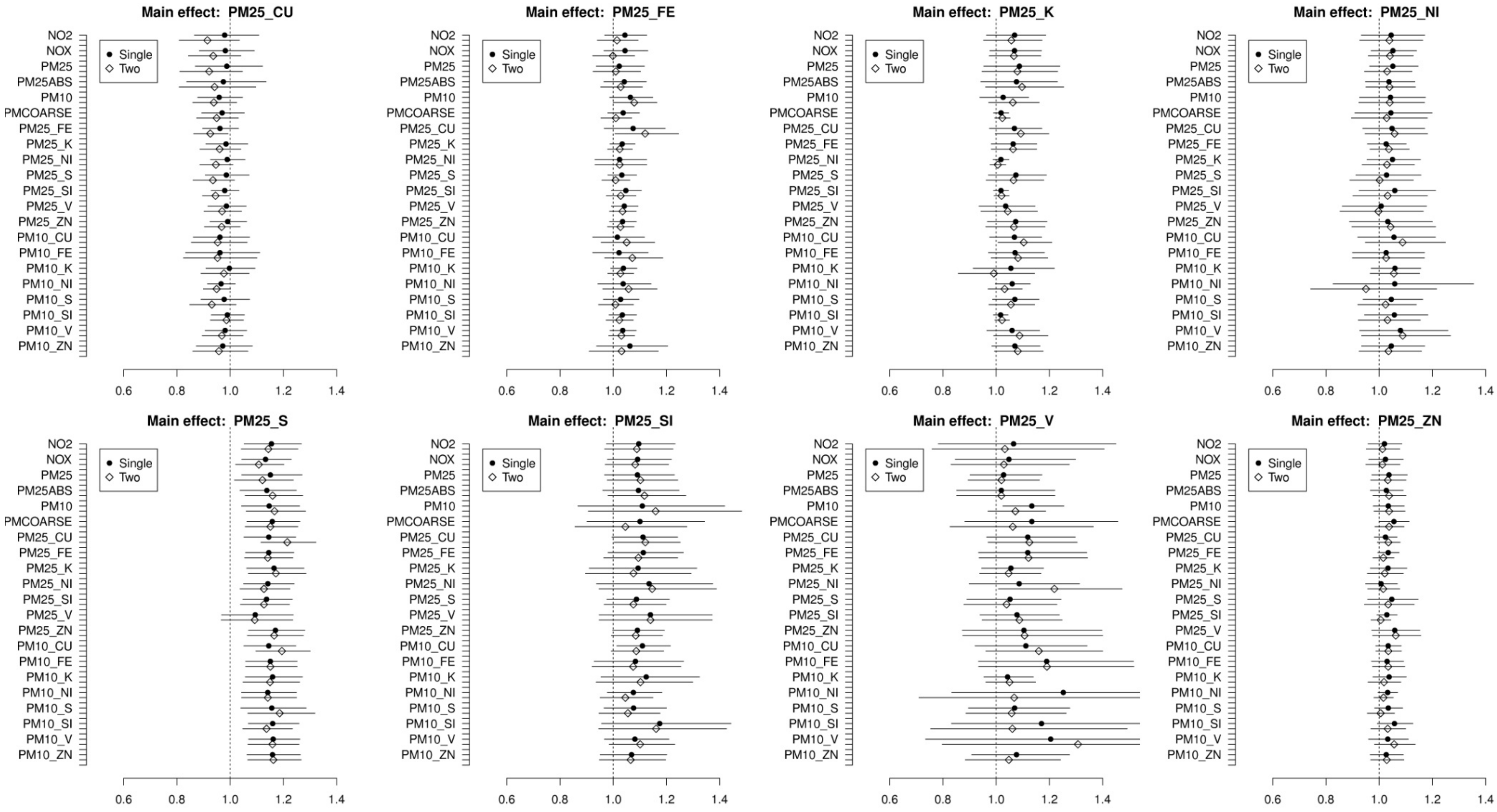

Figure S16. Two-pollutant model results. All estimates shown in an individual panel are HRs for the association between the element listed as the "Main effect" at the top of the panel and natural cause mortality. Estimates from single pollutant models for the same element vary because each single pollutant model is restricted to data from cohorts in which the correlation coefficient between the main effect element and the second pollutant is <0.7, such that the single pollutant HR for $PM_{2.5}Cu$ differs between the HR that is paired with NO_2 and the HR paired with $PM_{2.5}$ because of differences in the data included in each single-pollutant model.

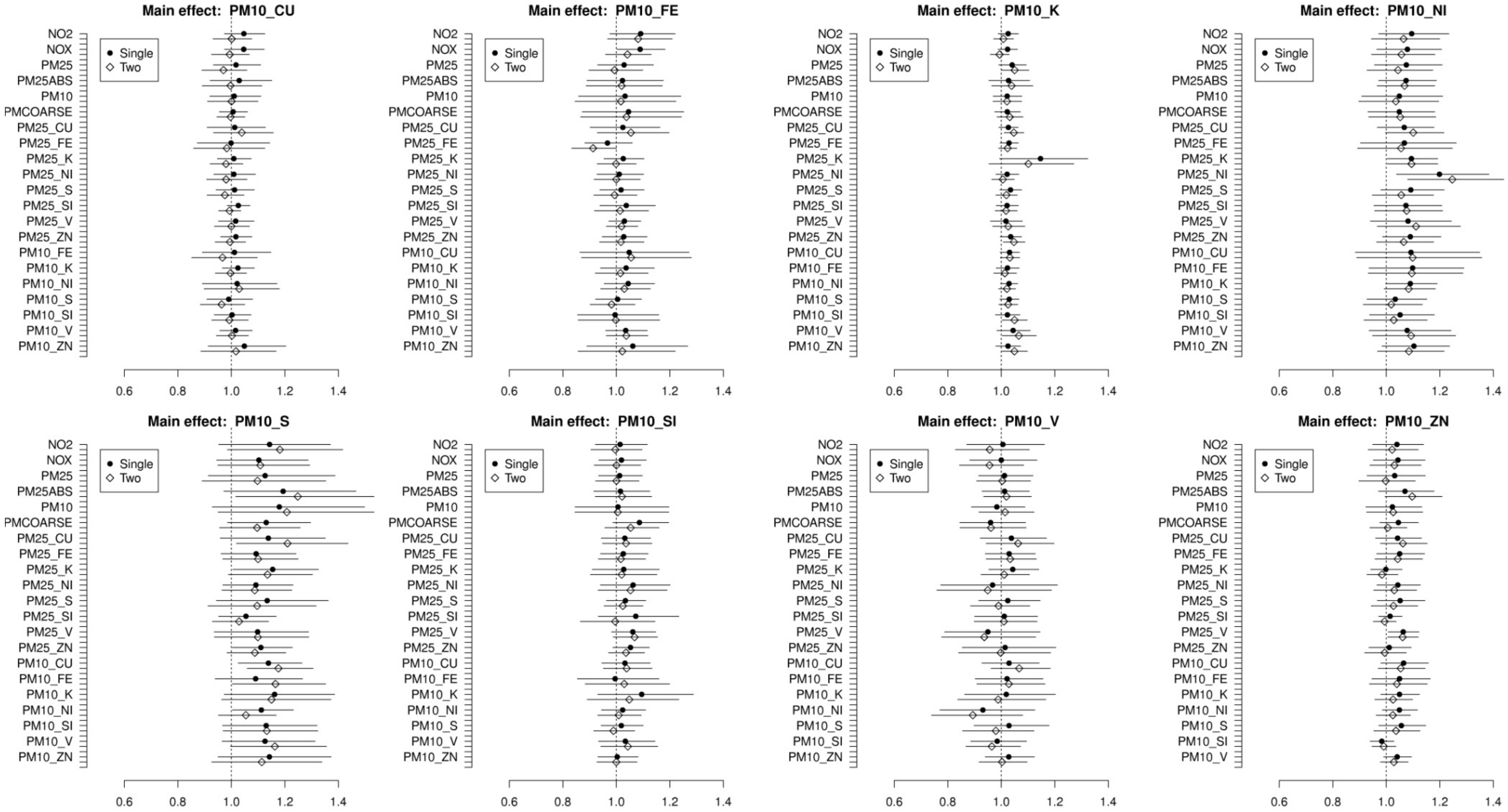

Figure S17. Two-pollutants model results. All estimates shown in an individual panel are HRs for the association between the element listed as the "Main effect" at the top of the panel and natural cause mortality. Estimates from single pollutant models for the same element vary because each single pollutant model is restricted to data from cohorts in which the correlation coefficient between the main effect element and the second pollutant is <0.7, such that the single pollutant HR for $PM_{10}Cu$ differs between the HR that is paired with NO_2 and the HR paired with $PM_{2.5}$ because of differences in the data included in each single-pollutant model.

Table S30. Association between natural cause mortality and exposure to particle composition: Results from random-effects meta-analyses (HRs and 95%-CIs) for the extended confounder models. Results for main model 3 and extended confounder models are based on same number of cohorts for each exposure measure.[a]

Exposure	Model 3	Model 3 + prevalent hypertension and physical activity[b]	Model 3 + prevalent hypertension, physical activity, prevalent diabetes, and cholesterol level[c]
$PM_{2.5}$ Cu	0.98 (0.92, 1.05)	0.98 (0.91, 1.06)	0.98 (0.90, 1.07)
$PM_{2.5}$ Fe	1.03 (0.98, 1.09)	1.03 (0.97, 1.10)	1.03 (0.97, 1.10)
$PM_{2.5}$ Zn	1.03 (0.99, 1.08)	1.04 (0.97, 1.12)	1.05 (0.97, 1.12)
$PM_{2.5}$ S	1.14 (1.06, 1.23)	1.13 (1.04, 1.21)	1.13 (1.04, 1.22)
$PM_{2.5}$ Ni	1.05 (0.97, 1.13)	1.04 (0.93, 1.17)	1.05 (0.93, 1.18)
$PM_{2.5}$ V	1.07 (0.93, 1.23)	1.06 (0.85, 1.32)	1.07 (0.87, 1.31)
$PM_{2.5}$ Si	1.09 (0.99, 1.19)	1.08 (0.96, 1.23)	1.10 (1.00, 1.22)
$PM_{2.5}$ K	1.06 (0.98, 1.14)	1.06 (0.96, 1.17)	1.07 (0.97, 1.17)
PM_{10} Cu	1.01 (0.95, 1.07)	1.00 (0.94, 1.07)	1.00 (0.93, 1.07)
PM_{10} Fe	1.02 (0.96, 1.09)	1.02 (0.96, 1.09)	1.02 (0.95, 1.10)
PM_{10} Zn	1.04 (0.98, 1.10)	1.04 (0.97, 1.10)	1.03 (0.97, 1.10)
PM_{10} S	1.09 (0.99, 1.19)	1.06 (0.97, 1.16)	1.06 (0.96, 1.18)
PM_{10} Ni	1.09 (1.00, 1.19)	1.09 (0.98, 1.21)	1.08 (0.96, 1.21)
PM_{10} V	1.03 (0.95, 1.12)	1.01 (0.89, 1.15)	1.01 (0.89, 1.15)
PM_{10} Si	1.03 (0.97, 1.10)	1.04 (0.96, 1.12)	1.03 (0.95, 1.12)
PM_{10} K	1.03 (1.00, 1.06)	1.03 (0.99, 1.08)	1.03 (0.99, 1.07)

[a]HRs are presented for the following increments: 5 ng/m³ $PM_{2.5}$ Cu, 100 ng/m³ $PM_{2.5}$ Fe, 10 ng/m³ $PM_{2.5}$ Zn, 200 ng/m³ $PM_{2.5}$ S, 1 ng/m³ $PM_{2.5}$ Ni, 2 ng/m³ $PM_{2.5}$ V, 100 ng/m³ $PM_{2.5}$ Si, 50 ng/m³ $PM_{2.5}$ K, 20 ng/m³ PM_{10} Cu, 500 ng/m³ PM_{10} Fe, 20 ng/m³ PM_{10} Zn, 200 ng/m³ PM_{10} S, 2 ng/m³ PM_{10} Ni, 3 ng/m³ PM_{10} V, 500 ng/m³ PM_{10} Si, and 100 ng/m³ PM_{10} K. [b]All cohorts included, except SAPALDIA (no info available). SALIA, VHM&PP, SIDRIA-Turin and SIDRIA-Rome have only hypertension information available. [c]All cohorts included, except SAPALDIA (no info available). SALIA and VHM&PP have no diabetes info available. Cholesterol level are available for FINRISK, HUBRO, KORA, VHM&PP, and E3N.

References

Beulens JWJ, Monninkhof EM, Verschuren WM, et al. 2010. Cohort profile: The EPIC-NL study. Int J Epidemiol 39:1170–8.

Boker LK, Van Noord PAH, Van Der Schouw YT, et al. 2001. Prospect-EPIC Utrecht: Study design and characteristics of the cohort population. Eur J Epidemiol 17:1047–53.

Cesaroni G, Badaloni C, Porta D, Forastiere F, Perucci CA. 2008. Comparison between various indices of exposure to traffic-related air pollution and their impact on respiratory health in adults. Occup Environ Med 65:683–90.

Clavel-Chapelon F, Jadand C, Goulard H, et al. 1996. E3N, a cohort study on cancer risk factors. Bull Cancer 83:1008–13.

Davey GK, Spencer EA, Appleby PN, Allen NE, Knox KH, Key TJ. 2003. EPIC-Oxford: Lifestyle characteristics and nutrient intakes in a cohort of 33 883 meat-eaters and 31 546 non meat-eaters in the UK. Public Health Nutr 6:259–68.

Downs SH, Schindler C, Liu L-S, et al. 2007. Reduced exposure to PM10 and attenuated age-related decline in lung function. N Engl J Med 357:2338–47.

Eriksson A, Ekbom A, Granath F, Hilding A, Efendic S, Östenson C. 2008. Psychological distress and risk of pre-diabetes and type 2 diabetes in a prospective study of swedish middle-aged men and women. Diabetic Med 25:834–42.

Gehring U, Heinrich J, Kramer U, et al. 2006. Long-term exposure to ambient air pollution and cardiopulmonary mortality in women. Epidemiology 17:545–51.

Guarrera S, Ricceri F, Polidoro S, et al. 2012. Association between total number of deaths, diabetes mellitus, incident cancers, and haplotypes in chromosomal region 8q24 in a prospective study. Am J Epidemiol 175:479–87.

Heinrich J, Thiering E, Rzehak P, et al. 2012. Long-term exposure to NO2 and PM10 and all-cause and cause-specific mortality in a prospective cohort of women. Occup Environ Med 70:179-86.

Holle R, Happich M, Löwel H, Wichmann HE. 2005. KORA - A research platform for population based health research. Gesundheitswesen 67(Suppl 1):S19–S25.

Lagergren M, Fratiglioni L, Hallberg IR, et al. 2004. A longitudinal study integrating population, care and social services data. The Swedish National study on Aging and Care (SNAC). Aging Clin Exp Res 16:158–68.

Lichtenstein P, Sullivan PF, Cnattingius S, et al. 2006. The Swedish Twin Registry in the third millennium: An update. Twin Res Hum Genet 9:875–82.

Magnusson PKE, Almqvist C, Rahman I, et al. 2013. The Swedish twin registry: Establishment of a biobank and other recent developments. Twin Res Hum Genet 16:317–29.

Pajunen P, Koukkunen H, Ketonen M, et al. 2005. The validity of the Finnish Hospital Discharge Register and Causes of Death Register data on coronary heart disease. Eur J Cardiovasc Prev Rehabil 12:132–7.

Riboli E, Hunt KJ, Slimani N, et al. 2002. European Prospective Investigation into Cancer and Nutrition (EPIC): Study populations and data collection. Public Health Nutr 5:1113–24.

Søgaard AJ, Selmer R, Bjertness E, Thelle D. 2004. The Oslo Health Study: The impact of self-selection in a large, population-based survey. Int J Equity Health 3:3.

Tjønneland A, Olsen A, Boll K, et al. 2007. Study design, exposure variables, and socioeconomic determinants of participation in Diet, Cancer and Health: A population-based prospective cohort study of 57,053 men and women in Denmark. Scand J Public Health 35:432–41.

Tolonen H, Salomaa V, Torppa J, Sivenius J, Immonen-Räihä P, Lehtonen A. 2007. The validation of the Finnish Hospital Discharge Register and Causes of Death Register data on stroke diagnoses. Eur J Cardiovasc Prev Rehabil 14:380–5.

Ulmer H, Bachmann J, Huber K, Concin H, Bischof H. 1997. Longitudinal observation of general health screening in Vorarlberg from 1986 to 1994. Wien Klin Wochenschr 109:160–4.

Ulmer H, Kelleher CC, Fitz-Simon N, Diem G, Concin H. 2007. Secular trends in cardiovascular risk factors: An age-period cohort analysis of 6 98 954 health examinations in 1 81 350 Austrian men and women. J Intern Med 261:566–76.

Vartiainen E, Laatikainen T, Peltonen M, et al. 2010. Thirty-five-year trends in cardiovascular risk factors in Finland. Int J Epidemiol 39:504–18.

Wändell P, Wajngot A, de Faire U, Hellénius M. 2007. Increased prevalence of diabetes among immigrants from non-European countries in 60-year-old men and women in Sweden. Diabetes Metab 33:30–6.
